# Engineered yeast genomes accurately assembled from pure and mixed samples

**DOI:** 10.1038/s41467-021-21656-9

**Published:** 2021-03-05

**Authors:** Joseph H. Collins, Kevin W. Keating, Trent R. Jones, Shravani Balaji, Celeste B. Marsan, Marina Çomo, Zachary J. Newlon, Tom Mitchell, Bryan Bartley, Aaron Adler, Nicholas Roehner, Eric M. Young

**Affiliations:** 1 Department of Chemical Engineering, Worcester Polytechnic Institute, Worcester, MA USA; 2 Synthetic Biology, Raytheon BBN Technologies, Cambridge, MA USA

**Keywords:** Next-generation sequencing, Genome assembly algorithms, Metabolic engineering, Synthetic biology

## Abstract

Yeast whole genome sequencing (WGS) lacks end-to-end workflows that identify genetic engineering. Here we present Prymetime, a tool that assembles yeast plasmids and chromosomes and annotates genetic engineering sequences. It is a hybrid workflow—it uses short and long reads as inputs to perform separate linear and circular assembly steps. This structure is necessary to accurately resolve genetic engineering sequences in plasmids and the genome. We show this by assembling diverse engineered yeasts, in some cases revealing unintended deletions and integrations. Furthermore, the resulting whole genomes are high quality, although the underlying assembly software does not consistently resolve highly repetitive genome features. Finally, we assemble plasmids and genome integrations from metagenomic sequencing, even with 1 engineered cell in 1000. This work is a blueprint for building WGS workflows and establishes WGS-based identification of yeast genetic engineering.

## Abstract (web summary)

The cost and complexity of whole genome sequencing limits its use in identifying and validating sequences used for genetic engineering and synthetic biology. Here the authors present Prymetime, an integrated workflow to sequence engineered strains and identify engineering in metagenomes.

## Introduction

Complete and accurate detection of genetic engineering is needed to validate strain engineering, protect intellectual property, monitor for release events, and detect engineered organisms in unknown samples. Whole genome sequencing (WGS) is an attractive detection method because it does not depend on specific sequence features and captures all sequences – including intended and unintended modifications. Yet, precise resolution of genetic engineering places strict requirements on a WGS workflow – genetic engineering signatures must be clearly identified within accurate, complete, and contiguous sequences.

Thus, a WGS workflow is needed for engineered organisms. In this work, we focus particularly on engineered yeasts. Yeasts are a crucial testbed for genome-scale design^1,2^, and accurate WGS will be necessary for validating synthesized eukaryotic genomes. Yeast are also cell factories for medicines^3,4^, fuels^5,6^, materials^7,8^, and chemicals^9,10^. These are derived from several species of baker’s yeast *Saccharomyces cerevisiae*^11–13^ and nonconventional yeasts like *Yarrowia lipolytica*^14–16^ and *Komagataella phaffii* (formerly *Pichia pastoris*)^17,18^. Given the economic importance and increasing use of engineered yeast cell factories, it is crucial that WGS methods are developed that can efficiently validate the presence of intended engineering and confirm the absence of unintended variation. Without WGS, the majority of yeast strains are currently validated with less comprehensive methods like PCR and targeted sequencing. These methods do not capture the unintended secondary mutations common in engineered organisms^19–23^. There are also many unpublished accounts of WGS revealing unexpected sequences and genome structures in engineered industrial strains. Taken together, this evidence challenges the assumption that an observed phenotype is the direct result of intended engineering, illuminating a possible explanation for variation between replicates and irreproducible findings – a common problem for biology-related disciplines^24^. Clearly, WGS must be used more broadly to detect and validate genetic engineering in yeasts.

Yeast engineering leaves many predictable sequence features in the genome, like standard plasmid sets with known replication origins and expression parts^25–28^, integrations^29–32^, gene knockouts^33^, and genome edits using RNA-guided endonucleases^34–39^. Many of these can be identified in a genome sequence with a tool such as BLAST^40^. Yet, engineered yeast present several obstacles to complete, accurate genome assembly. The high sequence identity in many engineered constructs, such as common plasmid elements or parts derived from the host genome, can cause identical sequences to be omitted^41,42^. Engineered yeast also have complex genome features like multiple deletions^13^, multiple plasmids with varying copy numbers^30^, many insertions^36^, and SCRaMbLEd chromosomes^43,44^. Furthermore, the scale of yeast engineering is increasing both in the fraction of a genome that may be rewritten^45,46^, and in the numbers of engineered strains created through adaptive laboratory evolution^47–49^ and combinatorial pathway engineering^50–54^. These iterative approaches result in many strains that are costly to sequence. These obstacles are in addition to typical complexities like naturally repetitive regions (telomeres and ribosomal DNA), rearrangements, and polyploidy. Each make accurate, complete, and contiguous yeast genomes difficult to attain without a significant allocation of resources.

A WGS workflow consists of five steps: DNA isolation, library preparation, sequencing, assembly, and annotation. First, genomic DNA is purified using one of a variety of methods, including phenol-choloroform, bead beating, or enzymatic lysis^55^. Second, the sequencing library is prepared by attaching adapters and barcodes. This can be done via ligation, which involves shearing the DNA to create free ends for DNA ligase to attach adapters, or tagmentation, which randomly inserts adapter attachment points without shearing^56^. Third, the library is sequenced with a next-generation sequencing (NGS) platform that either generates short reads (150–300 base pairs long) with high nucleotide accuracy^56^ or long reads (1.5 kilobases to megabases long) with lower accuracy^57^. The average read length and the number of reads (genome coverage) output by the NGS platform is dependent on sequencing technology and the preceding DNA isolation and adapter attachment steps^58^. Fourth, the reads are computationally assembled into a final genome sequence with software that uses either an overlap-layout-consensus (OLC) or De Bruijn graph (DBG) algorithm^59^. OLC and DBG assemblers are further classified into short read only, hybrid (both short and long reads), or long read with error correction. Both hybrid and long read with error correction assembly approaches currently hold the most promise to achieve accurate genome sequence and structure at low read depths, primarily because two independent technologies validate basecalls. However, this entails the use of two sequencing technologies, thereby increasing costs and time. Fifth, an annotation is performed. Eukaryotic annotation involves first predicting genes in the genome sequence, followed by functional annotation^60^. However, features like genetic engineering parts, telomeres, centromeres, mitochondrial DNA, and natural plasmids are often not annotated, and several are by convention not included in the final assembly.

In this work, we develop an inexpensive WGS workflow designed to detect genetic engineering in pure and mixed samples of engineered yeast. To accomplish this, we optimized each of the five steps in the WGS workflow in order to correctly resolve all engineering sequences in a heavily engineered yeast strain. We first improved DNA isolation and sequencing library preparation to increase representation of reads from circular DNA molecules. We then used a combination of long- and short-read sequencing from inexpensive sequencing platforms to achieve high coverage at low cost. We integrated two different assemblers to resolve both circular plasmids and linear chromosomes accurately. We developed an annotation approach based on a user-input list of genetic parts to clearly identify common signatures of engineering. Using this approach, we also annotated centromeres, telomeres, origins of replication, and mitochondrial DNA in order to put observed genetic engineering in context with the rest of the genome. The resulting workflow is named Prymetime, "Pipeline for Recombinant Yeast genoMEs That Identifies Markers of Engineering." Through a variety of demonstrations, we show that Prymetime can validate genetic engineering, produce high quality whole genome sequences, and detect engineering in metagenomic samples. This tool is broadly useful for strain validation, release monitoring, protecting intellectual property, and investigating engineering in unknown samples.

## Results

### Optimizing nanopore sequencing library preparation for engineered yeasts

**Fig. 1:**
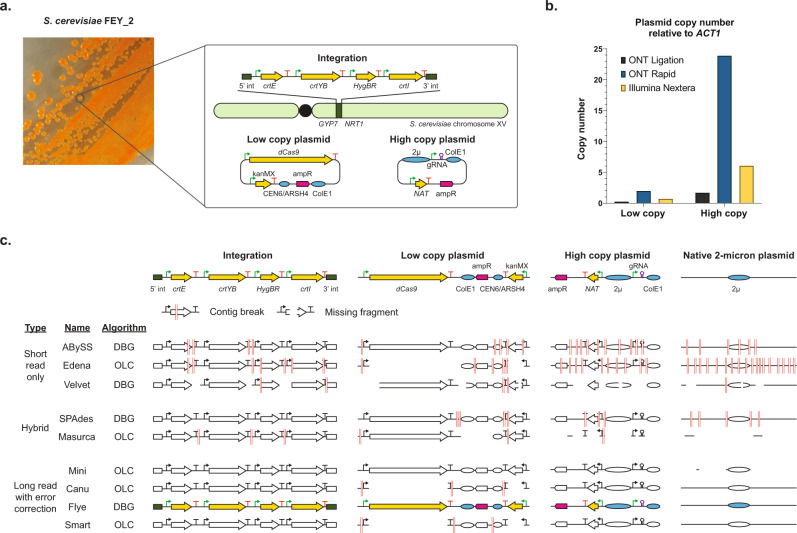
Detection of engineering signatures in *S. cerevisiae* FEY_2. **a** Photograph of FEY_2 streaked onto an agar plate, showing a functional carotenoid pathway. The illustration shows the engineering signatures comprising FEY_2, which included a carotenoid pathway chromosomal integration, a low copy plasmid expressing dCas9, and a high copy plasmid expressing gRNA. **b** Approximate copy number from genomic DNA libraries prepared by Oxford Nanopore Technologies’ (ONT) Ligation and Rapid kit and Illumina’s Nextera kit for the low copy and high copy plasmids in FEY_2. **c** BLASTN results from querying known engineering signatures against assemblies. The genome assemblers were categorized as short-read only, hybrid, or long read with error correction. The underlying algorithm type of each assembler, De Bruijn graph (DBG) or overlap-layour-consensus (OLC), is also shown. Failure modes of genome assemblies were shown as red lines (contig break) and white spaces (missing fragment). The colored pathways and plasmids represent assemblies where all engineering signatures were found in contiguous sequences.

At the beginning, we set a standard that a genome assembly workflow must be able to resolve chromosomal integrations and multiple plasmids used in yeast engineering. Therefore, we built a *S. cerevisiae* CEN.PK113 strain, FEY_2, containing an integrated carotenoid pathway, the native 2μ plasmid, a dCas9 plasmid, and a gRNA plasmid, shown in Fig. 1a. Initially, we prepared sequencing libraries of FEY_2 with the Oxford Nanopore Technologies (ONT) ligation kit. Sequencing these initial libraries had low average read length that varied from run to run, possibly because of differential DNA shearing during isolation. To limit this, we developed a gentle genomic DNA isolation protocol which increased average nanopore read length and reduced variance (Supplementary Figure S1). However, the sequencing results contained few reads from plasmids, as determined by comparing the average normalized mapped reads of the plasmid antibiotic selection markers to those of the *ACT1* genomic locus using Minimap2^61^. We could isolate plasmids from FEY_2 using a yeast miniprep kit, so we reasoned that the sequencing library preparation step was so gentle that it was not linearizing circular plasmids for adapter ligation. Thus, we turned to a tagmentation library preparation method, the ONT Rapid kit. The improvement in average normalized mapped plasmid reads is shown in Fig. 1b. We were reassured that the 2:1 and 20:1 marker to *ACT1* read coverage ratios for each plasmid are equivalent to the approximate plasmid copy number in yeast for each origin^12,62^. Furthermore, tagmentation also increased the representation of other circular elements like the native 2μ plasmid and mitochondrial DNA. These results indicate that tagmentation is a key to achieving long average read lengths while also generating linear molecules from small circular DNA so that they can pass through the nanopore flow cell. Whereas tagmentation may have a slight AT sequence bias and perform poorly in extreme GC genomes, this is not the case with our yeasts. Thus, with gentle isolation and tagmentation, nanopore sequencing of FEY_2 resulted in adequate representation of plasmid reads.

### Developing a de novo assembly workflow for complete, contiguous plasmids and integrations

Once we achieved appropriate read representation, we evaluated nine assembly algorithms with the stringent requirement that all chromosomes and plasmids must be complete, accurate, and contiguous. This is particularly stringent for the three plasmids in FEY_2 because they each have significant sequence identity between each other and the genome. The assemblers tested included the short-read only OLC assembler Edena^63^, the short-read only DBG assemblers ABySS^64^ and Velvet^65^, the hybrid OLC assembler Masurca^66^, hybrid DBG assembler HybridSPAdes^67^, the long-read OLC assemblers MiniASM^68^, Canu^69^, and SMARTdenovo^70^, and the long-read DBG assembler Flye^71^. The long-read assemblers, because of higher error rates^57^, only provide a "skeleton" for mapping additional reads^72–76^. Therefore, all long-read assemblies were polished with Medaka^77^, Racon^78^, and Pilon^79^.

We used the optimized library preparation to obtain long reads at 60X genome coverage from the ONT MinION and short reads at 125X genome coverage from the Illumina iSeq 100. This common set of reads was used by each assembler, and the resulting genome assembly was analyzed using BLASTN for the presence of the integrated pathway, both plasmids, and the native 2μ plasmid. A visual representation of the BLASTN results is shown in Fig. 1a. The engineering features were rarely complete or contiguous. The short-read only de novo assemblers ABySS, Edena, and Velvet returned a fragmented, incomplete pathway and plasmids. The hybrid assemblers SPAdes and Masurca produced more complete sequences than the short-read only assemblers, but the genome integration was fragmented, and Masurca also omitted portions of the three plasmids. The long-read de novo assemblers MiniASM, Canu, Flye, and SMARTdenovo each returned a single contiguous sequence for the genome integration, yet, MiniASM, Canu, and SMARTdenovo omitted sections of the three plasmids. Only Flye eventually returned the genome integration and each plasmid correctly in contiguous sequences.

Of the assemblies missing large portions of at least one of the three plasmids, almost all were generated with an OLC assembler. These algorithms use an All-versus-All consensus step that may discard highly identical sequences in order to reach consensus. To investigate this further, we used BLASTN at each step in the OLC-based Canu pipeline to determine when sequences were omitted. We noted that the complete low-copy plasmid was initially present before the consensus step, and was then lost in the final assembly. It seems that Canu discarded the plasmid at a certain threshold during the consensus step, likely because of high sequence identity with the other plasmids. In contrast, the DBG assemblers Flye, ABySS, and SPAdes did not omit sections of plasmids. DBG algorithms split reads into shorter k-mers followed by a Eulerian walk approach to construct contigs, thus DBG may be less prone to discarding highly identical sequences^80^. Though complete, the plasmid sequences from ABySS and SPAdes were fragmented, while Flye assembled each plasmid into a single contiguous sequence. This is possibly because ABySS and SPAdes are hybrid assemblers that assemble short reads first, then use long reads to piece together contigs. This makes them subject to the same pitfalls that plague short-read assemblers, in that the reads do not effectively span sequences with high identity. Thus, Flye, as a DBG assembler that assembles long reads first, produces higher contiguity and better resolution of sequences with high identity. These findings reinforce that genome assembly quality is dependent on high-quality long-read data and a de novo assembly approach.

While the plasmid contigs from Flye were complete and contiguous, they were longer than expected. Further inspection revealed that the contigs consisted of several repeats of the expected plasmid sequence. This is a common problem for long-read assemblers, as they use linear logic to merge contigs^69,81^. To obtain structurally representative plasmid contigs, we chose to re-assemble them with Unicycler, software that was built to assemble circular contigs from bacterial sequencing data^82^. To do this, we sent contigs either identified by Flye as circular or identified by mummer as repetitive to Unicycler. Reassembly of plasmids with Unicycler improved the accuracy as measured by BLASTN and length of the contigs for the three plasmids in FEY_2 (Supplementary Fig. S2).

### Resolving engineering signatures in a collection of engineered yeasts

**Fig. 2:**
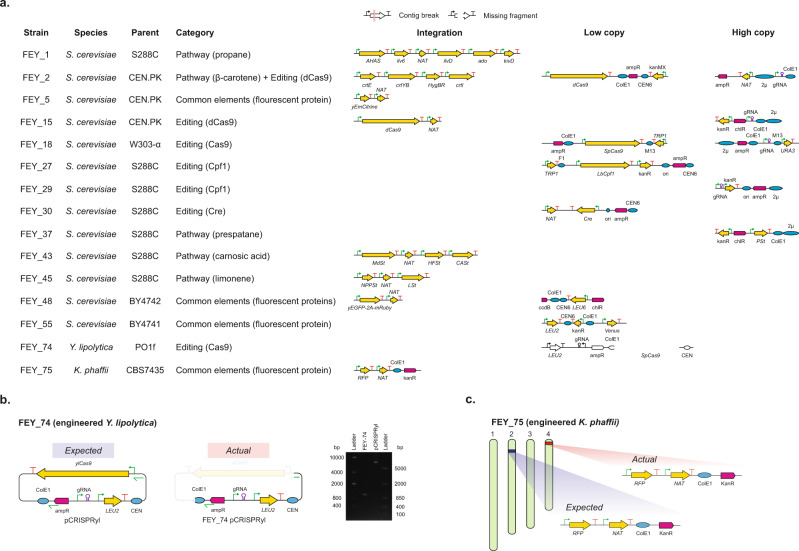
Resolving signatures of engineering from the panel of engineered yeast strains. **a** Visual representation of the BLASTN results from querying known engineering signatures against Prymetime-assembled genome assemblies of the 15 engineered strains. Failure modes of genome assemblies were shown as red lines (contig break) and white spaces (missing fragment). The colored pathways and plasmids represent assemblies where all engineering signatures were found in contiguous sequences. **b** The expected CRISPR-Cas9 expression vector for FEY_74, an engineered *Y. lipolytica* strain, and the actual plasmid from the Prymetime genome assembly. The DNA agarose gel confirms the missing Cas9 cassette from the FEY_74 strain in comparison to the original pCRISPRyl plasmid. The agarose gel represents one experiment, where the PCR products of the pCRISPRyl plasmid and FEY_74 plasmid were processed in parallel. **c** Illustration showing the expected location of the RFP integration cassette into chromosome II of FEY_75, an engineered *K. phaffii* strain, and the actual location of the cassette into chromosome IV.

We next validated our assembly approach on a collection of engineered laboratory and nonconventional yeast. We constructed 15 strains from *S. cerevisiae* S288C, CEN.PK113-7D, W303-*α*, BY4741, BY4742, and *K. phaffii* ATCC 76273 (CBS 7435)^83,84^ and *Y. lipolytica* ATCC MYA-2613 (Po1f)^85^. Plasmids designed to construct transcriptional units for this study are described in Supplementary Fig. S3. A description of each strain is shown in Fig. 2a, with more details in Supplementary Table S1 and Supplementary Fig. S4. Engineering signatures were inserted into the genome or maintained on episomal plasmids. *S. cerevisiae* integrations were targeted to the HO locus^26^ or between *NRT1* and *GYP7* in chromosome XV^38,51^. *S. cerevisiae* plasmids consisted of custom TypeIIS-compatible yeast shuttle vectors with either *S. cerevisiae* replicon (2μ or CEN6/ARSH4). Engineering was broadly categorized into biosynthetic pathways, gene editing components, deletions, and synthetic biology elements. Biosynthetic pathways included propane^86^, 2β-carotene^87^, prespatane^88^, carnosic acid^89^, and limonene^90,91^. Genome editing associated tools included SpCas9^34^, dCas9^35^, LbCpf1^38^, FnCpf1^37^, and Cre recombinase^33^. Deletions included the synthetic auxotrophies already present in *S. cerevisiae* W303-*α*, BY4741, BY4742, and *Y. lipolytica* Po1f. Synthetic biology elements included fluorescent proteins^92,93^ and the 2A sequence^94^. The engineered *Y. lipolytica* strain "FEY_74" contained the CRISPR-Cas9 plasmid pCRISPRyl^39^. The engineered *K. phaffii* strain "FEY_75" contained a recombinase-integrated red fluorescent protein (RFP) cassette^28^.

We sequenced this collection with the ONT MinION and the Illumina iSeq 100 systems using our optimized library preparation protocols. The combined assembly approach using Flye and re-assembly of circular contigs with Unicycler captured each engineering signature in each *S. cerevisiae* genetic background as measured by BLASTN of the reference sequence against the assembly. Shown in Fig. 2a, the approach resolved seven different genome integrations in two genome loci and eleven different plasmids. The BLASTN metrics are in Supplementary Table S2. To further demonstrate the necessity of a combined assembly approach, we repeated assembly with Flye alone. The additional Unicycler step improves plasmid length and accuracy in every strain, not just FEY_2 (Supplementary Fig. S5). No sequence complexities, like the type of gene (metabolic, selective, editing, or reporter), parts identical to the genome (Ptef1, Pgal10), or plasmid copy number, affected the accuracy or structural completeness of the assemblies.

The genome assemblies from the two engineered nonconventional yeasts – *Y. lipolytica* strain FEY_74 and *K. phaffii* strain FEY_75 – revealed unintentional edits. FEY_74 was intended to contain the pCRISPR-yl plasmid^39^, yet the contig from the genome assembly was missing the entire Cas9 transcription unit and a portion of the *E. coli* origin of replication, shown in Fig. 2b. Inspection of the raw reads failed to identify a single read with the missing sequence. We performed a genomic DNA isolation and a yeast plasmid miniprep on FEY_74 and transformed the resulting DNA back into *E. coli*, yet did not observe any colonies. This indicates that the disrupted origin of replication in the assembly reflects an actual unintended loss rather than an assembly error. This was further confirmed by PCR of DNA isolated from FEY_74 with primers spanning the missing region of the plasmid. The length of the PCR product indicated that the sequence was indeed missing (Fig. 2b). Similarly, FEY_75 was designed to have an RFP transcription unit integrated into chromosome II (Fig. 2c). The entire pathway was found by BLASTN in the FEY_75 genome, but analysis revealed that the pathway was actually integrated into chromosome IV. This was further confirmed by PCR of the integration site in chromosome II, which was negative, yet the strain remained nourseothricin resistant and RFP positive. These results indicate that a combined assembly approach can be used to find and accurately reproduce engineering, which is useful for both strain quality control and identification of engineering in unknown samples.

### Whole genome assembly quality

**Fig. 3:**
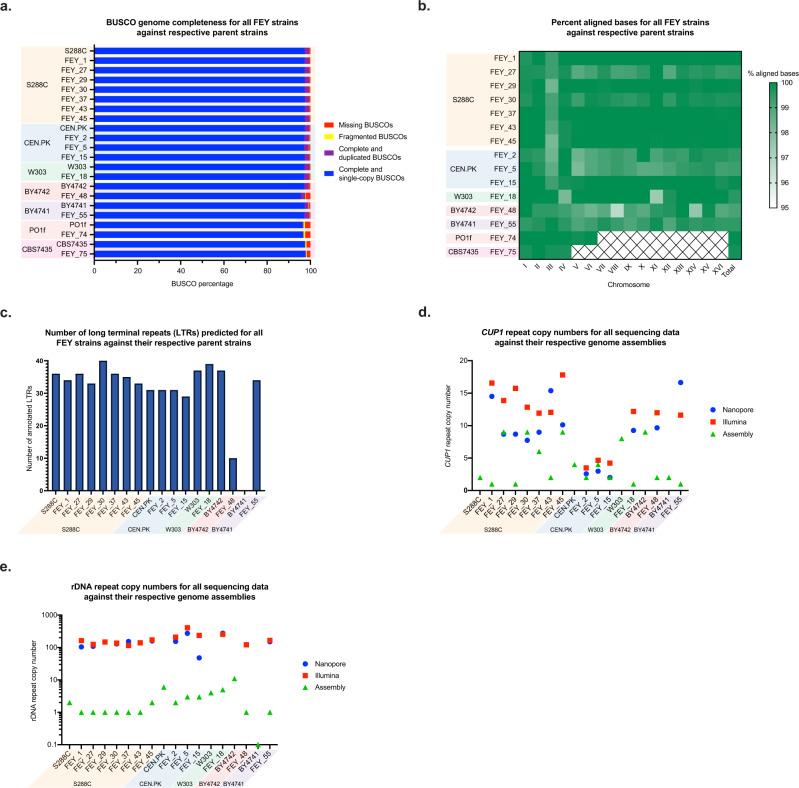
Whole genome assembly quality for the panel of engineered yeast strains. **a** BUSCO genome completeness score for all engineered yeast genome assemblies and their respective reference parent strain genome assemblies. **b** Percentage of aligned bases for each chromosome of the reference parent strain assemblies captured by the engineered yeast assemblies. We could not determine the 16 chromosomes for the reference BY4741 assembly due to its discontiguity, so the FEY_55 assembly was compared to the BY4742 reference assembly. **c** The number of long terminal repeats (LTRs) predicted by LTRpred for all engineered *S. cerevisiae* genome assemblies and their respective reference parent strain genome assemblies. **d** The approximate copy number of *CUP1* repeats in the genome assembly, raw Nanopore reads, and raw Illumina reads for all engineered *S. cerevisiae* strains, along with the *CUP1* copy number in the respective reference parent strain assemblies. **e** The approximate copy number of rDNA repeats in the genome assembly, raw Nanopore reads, and raw Illumina reads for all engineered *S. cerevisiae* strains, along with the rDNA copy number in the respective reference parent strain assemblies. These are also tabulated in Supplementary Table S4.

After achieving assembly of all engineering sequences, we then assessed whole genome quality of the 15 engineered assemblies and genomes from the parent nonconventional yeasts *Y. lipolyitica* Po1f and *K. phaffii* CBS7435. Each genome had high contiguity, sequence accuracy, and genome completeness as measured by Benchmarking Universal Single-Copy Orthologs (BUSCO) score^95^, calculated using the Saccharomycetales dataset (Fig. 3a) and percent aligned reads to the parent genome (Fig. 3b). Percent unmapped reads for each genome are provided in Supplementary Table S3. Whole genome alignments for each genome compared to the parent with Mauve^96^ are presented in Supplementary Figs. S6 and S7. The resequenced *Y. lipolytica* Po1f and *K. phaffii* CBS7435 strains were improved compared to the publicly available genomes^16,84^ by several metrics (Supplementary Fig. S8). Notably, there are 6 more essential genes recovered in the resequenced *K. phaffii* assembly and 13 more essential genes recovered in the resequenced *Y. lipolytica* assembly.

The final test of completeness is whether a chromosome is resolved from a telomere, through the centromere, to the other telomere. We compared each engineered *S. cerevisiae* assembly to its respective reference assembly to quantify the number of complete telomere-to-telomere contigs (Supplementary Fig. S9). We found that 76% of chromosomes are complete, except the telomeres. Analysis of several smaller contigs in the assemblies reveals them to be telomeric or ribosomal DNA (rDNA) sequences. This result shows that the genomes are essentially complete, save misassembly of highly repetitive genomic sequences and telomeres.

Next, we further assessed repetitive DNA elements in each *S. cerevisiae* genome, finding that repetitive elements like long terminal repeats (LTRs), *CUP1* repeats, and rDNA are resolved with comparable copy number to the reference genomes (Fig. 3c, d, e, respectively). However, the *CUP1* and rDNA repeat copy numbers were underrepresented in both our assemblies and the reference assemblies when compared to the approximate copy number of the raw Nanopore and Illumina reads. The *S. cerevisiaeCUP1* copy number is highly variable, ranging from zero to 79^97^, while the rDNA copy number is known to range between 100 and 200^98^. Tandem repeats such as *CUP1* and rDNA are a common problem for all de novo assemblers and are often collapsed during assembly^99^.

**Fig. 4:**
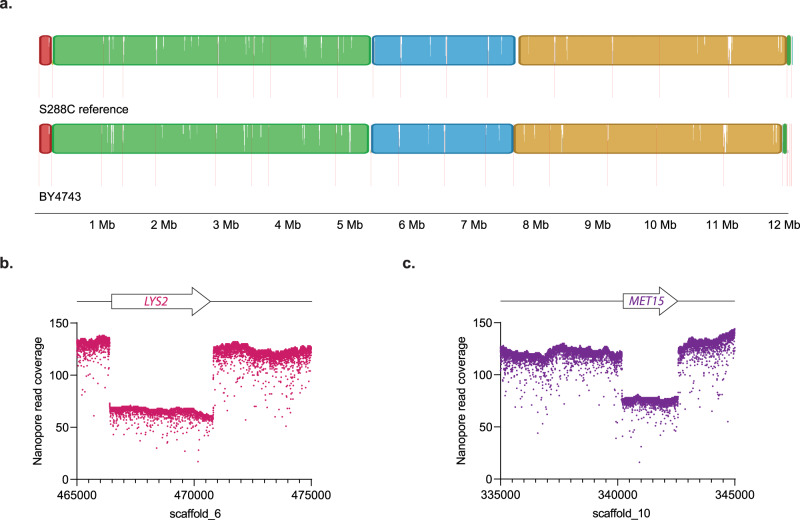
Genome assembly analysis of the heterozygous diploid *S. cerevisiae* strain BY4743. **a** Mauve genome alignment between the *S. cerevisiae* S288C reference assembly and the *S. cerevisiae* BY4743 genome assembly. The colored blocks represent regions of the genomes that align, while the vertical red lines indicate a new contig. **b** Nanopore read coverage around the heterozygous *LYS2* gene. **c** Nanopore read coverage around the heterozygous *MET15* gene.

Every strain investigated in the above collection is haploid. Therefore, we sequenced the heterozygous diploid strain *S. cerevisiae* BY4743. The resulting assembly is similar to *S. cerevisiae* S288C (Fig. 4a). Thus, this assembly approach cannot resolve ploidy. However, the *LYS2* and *MET15* heterozygous deletions can be clearly resolved by mapping average read count (Fig. 4b, c, respectively).

Taken together, these results indicate that the genome assemblies generated by the combined assembly approach are structurally correct, accurate, and complete, although telomeres, repeat elements, and ploidy remain a challenge to accurately reproduce. This is currently a challenge in the field of de novo genome assembly.

### Annotating and visualizing engineering and genome features

The last step in WGS, annotation, does not usually identify genetic engineering sequences. Therefore, we developed an engineering annotation step and applied it to the collection of 15 engineered yeasts. We first wrote an automated BLASTN script to find standard yeast genetic engineering parts and genome features. Standard parts include the CEN6/ARSH4 and 2μ replication origins, selection markers, promoters, and terminators. Genome features include centromeres, telomeres, and mitochondrial DNA, which were sourced for each parent strain from the Saccharomyces Genome Database^100^. This list of parts and features is simply a FASTA file, which can be easily modified and updated to find any sequence of interest in genome assemblies.

**Fig. 5:**
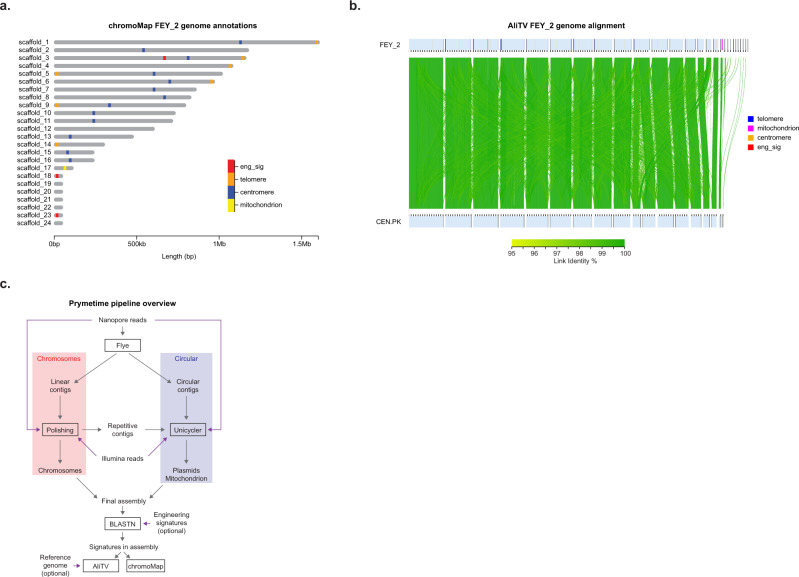
Visualizing engineering and genome features, and the Prymetime pipeline. **a** chromoMap interactive visualization displaying engineering signatures and structural elements identified in the FEY_2 genome assembly. **b** AliTV interactive visualization of the FEY_2 genome assembly aligned against its parent CEN.PK113-7D genome assembly. Engineering signatures and structural elements are also annotated. **c** Overview of Prymetime genome assembly pipeline.

We then fed the BLASTN results to two interactive genome viewers – chromoMap^101^ and AliTV^102^. ChromoMap highlights the parts and features within each contig in the assembly. AliTV does the same, but also aligns the assembly to the parent strain using lastz^103^. This can highlight potential unintended changes like chromosomal rearrangements. The chromoMap visualization for FEY_2 (Fig. 5a) shows the integration in scaffold_3, and the two engineered plasmids in scaffold_18 and scaffold_23. The output is interactive, so hovering over the engineering blocks will display which parts were identified. Using this approach, the plasmids can be differentiated from other small contigs by the presence of the origins of replication and other engineering sequences. In the AliTV visualization, the high sequence identity and contiguity of the engineered as compared to unengineered *S. cerevisiae* CEN.PK is apparent. The AliTV visualization is also interactive and customizable, and is particularly useful to determine how contigs from the assembly align to the reference assembly.

### Creating an automated pipeline

Optimization of each of the five steps of genome assembly led to a final set of methods and software that can accurately reproduce and visualize genetic engineering in highly accurate yeast genomes. We integrated each of the software steps into a single Dockerized tool that we call Prymetime, "Pipeline for Recombinant Yeast genoMEs That Identifies Markers of Engineering." The final pipeline is depicted in Fig. 5c. The software accepts long reads and short reads, and optionally accepts a list of sequences of interest and a reference genome. It outputs two interactive visualizations of the genome. Each visualization of the 15 engineered strains is depicted in Supplementary Figs. S10–S17.

As a final demonstration, we tested each step in the Prymetime workflow with a set of publicly available raw reads for *S. cerevisiae* CEN.PK113-7D^74,104^, assessing the quality at each step (Supplementary Fig. S18). First, we evaluated the contigs from Flye step, determining that 40X long-read genome coverage is sufficient to match the reference assembly. Then, we evaluated the polishing step, which demonstrated that at least 40X short-read genome coverage is needed to achieve high identity to the reference, BUSCO, and percentage of *S. cerevisiae* S288C CDSs (Supplementary Tables S5–S8). Using the chromoMap visualization output from Prymetime, the CEN.PK113-7D assembly correctly captures the centromeric sequences, but not the telomeric sequences (Supplementary Fig. S19). This corroborates the observations from the engineered genomes. Using different de novo assemblers still does not solve this problem (Supplementary Table S9), thus Flye remains the best underlying assembly software for assembly of accurate, complete, and contiguous genetic engineering sequences. A detailed illustration of the full Prymetime workflow is shown in Supplementary Fig. S20. These results confirm the Prymetime software workflow is as accurate as possible and show that at least 40X genome coverage for both long- and short-read sequencing data is needed to achieve the highest quality genomes.

### Resolving signatures of engineering in an in silico metagenome assembly

**Fig. 6:**
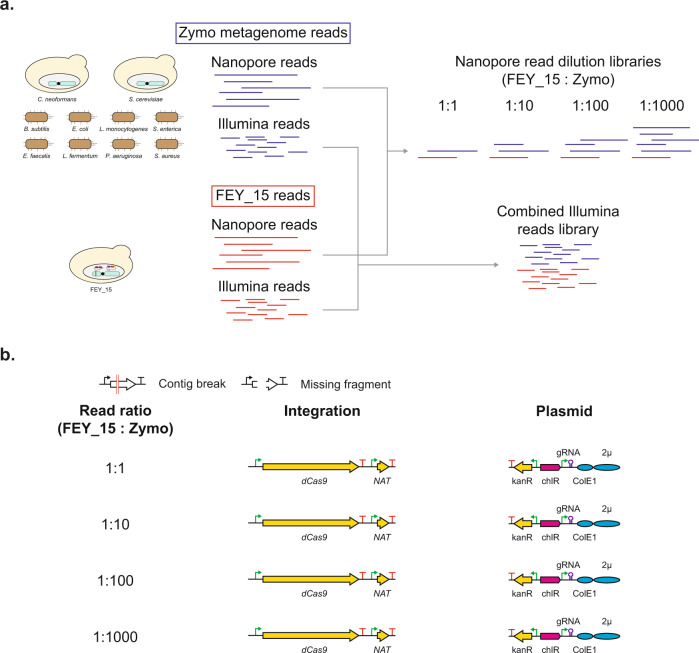
Resolving signatures of engineering in an in silico metagenome assembly. **a** Publicly available reads from Zymo’s mock metagenome were combined with reads from the engineered *S. cerevisiae* strain FEY_15. **b** Visual representation of the BLASTN results for the in silico metagenome. Failure modes of genome assemblies were shown as red lines (contig break) and white spaces (missing fragment). The colored pathways and plasmids represent assemblies where all engineering signatures were found in contiguous sequences.

To demonstrate a use case for Prymetime, we attempted to resolve engineering signatures in a metagenome. Publicly available reads from the Zymo mock metagenome were combined with reads from the FEY_15 strain to simulate detection of an engineered strain in a mixed sample. The mock metagenome consists of eight bacteria species – *Bacillus subtilis*, *Enterococcus faecalis*, *Escherichia coli*, *Lactobacillus fermentum*, *Listeria monocytogenes*, *Pseudomonas aeruginosa*, *Salmonella enterica*, and *Staphylococcus aureus* – and two yeast species – *Cryptococcus neoformans* and *Saccharomyces cerevisiae*^105^. To simulate different abundance levels, the FEY_15 nanopore reads were diluted with increasing numbers of Zymo metagenome reads at approximate ratios of 1:1, 1:10, 1:100, and 1:1000 based on number of base pairs. All of the FEY_15 and Zymo metagenome Illumina reads were combined together at an approximate ratio of 1:20 (Fig. 6a). These read sets were then used for Prymetime assembly. In each read set, the integration and plasmid of FEY_15 were completely resolved (Fig. 6b). This shows that synthetic biology parts, and their context, can be resolved in mixed samples by Prymetime.

## Discussion

This work develops an integrated workflow for WGS of engineered yeasts which may be extensible to all eukaryotes with a mixture of linear and circular sequences. The workflow consists of gentle gDNA isolation, tagmentation, long- and short-read NGS, accurate de novo assembly of both linear and circular elements, and annotation of genetic engineering parts and genome features. Using this, diverse engineering signatures can be resolved in complete, contiguous sequences even with multiple similar plasmids in one strain. The resulting whole genome quality is comparable to high-quality reference assemblies, therefore, it is possible to generate accurate genome assemblies both before and after engineering. This permits verification of genetic engineering in yeasts with WGS to validate strain engineering. Further, the workflow performs well using metagenomic data, permitting detection of yeast engineering in mixed samples.

This work demonstrates the challenges in making effective WGS-based workflows. Interestingly, we found that only the Flye assembly algorithm supported accurate resolution of genetic engineering in complete, contiguous sequences. We observed that sequence omission commonly occurred with assemblers built around OLC algorithms, which struggle to reproduce the expected representation and resolution of repeats^68,69,106^. Furthermore, we observed that short-read and hybrid assemblers commonly produced fragmented sequences. Thus, Flye, as the only long-read DBG assembler, was consistently the best at resolving genetic engineering signatures. These observations highlight the difficulty of applying otherwise effective genome assembly software to engineered yeasts, which have highly identical genetic engineering signatures and repetitive genome features. Furthermore, all assemblers collapsed repetitive genome features and struggled to resolve telomeres. This limits the ability of the tool to detect variations in rDNA, SNPs, and rare variants. Based on our results, assemblers aiming to improve these areas should be benchmarked against the overall performance of Flye.

To date, WGS has rarely been used in strain engineering cycles due to the barriers of cost, time, and required bioinformatics expertise. The WGS workflow we developed with the inexpensive ONT MinION and Illumina iSeq 100 platforms and the integrated, dockerized Prymetime software package overcomes these barriers. With Prymetime, we were able to achieve high-quality genomes at relatively low read depth, finding that 40X for both long and short reads was sufficient for high accuracy, completeness, and contiguity of genetic engineering sequences, and quality whole genomes. With 40X read depth, up to 30 *S. cerevisiae* genomes can be sequenced on one MinION flow cell and up to 4 genomes can be sequenced on one Illumina iSeq flow cell. This is because approximately 0.5 Gb is needed for 40X read depth of the 13.4 Mb *S. cerevisiae* genome (factoring in collapsed rDNA repeats^107^) and our typical yield is approximately 15 Gb from the MinION and 2.4 Gb from the iSeq 100. Not accounting for labor, this level of multiplexing would cost around $200 per genome. The entire workflow is fast – it takes under a week to start from a single colony and acquire a genome assembly, requiring only 15 h of hands-on time. Our workflow requires only a few coding steps – future users can simply load NGS reads and run the Prymetime script to detect and validate genetic engineering.

## Methods

### Strains and media

Parent strains for all engineered strains are shown in Supplementary Table S1. All yeast strains were grown in yeast extract-peptone-dextrose (YPD) or synthetic complete (SC)+glucose media. YPD consisted of 30 g/L YEP (10 g/L yeast extract + 20 g/L peptone, Sunrise Science, 1877-1KG) and 20 g/L glucose (Alfa Aesar, A16828). SC + glucose media consisted of 6.71 g/L of YNB+Nitrogen (1.71 g/L yeast nitrogen base + 5 g/L ammonium sulfate, Sunrise Science, 1501-250), 20 g/L glucose, and a formulation of complete synthetic media (CSM). CSM formulations were (1) CSM-Leu: 0.65 g/L CSM-His-Leu-Ura (Sunrise Science, 1015-010) + 0.02 g/L Histidine (Sunrise Science, 1978-010) + 0.02 g/L Uracil (Sunrise Science, 1906-010) and (2) CSM-Trp-Ura: 0.62 g/L CSM-Leu-Trp-Ura (Sunrise Science, 1017-010) + 0.1 g/L Leucine (Sunrise Science, 1980-010). For the *K. phaffii* transformation, 2xYPD was prepared with 75 g/L YEP plus 20 g/L glucose, and YPDS plates were prepared by supplementing YPD agar with 1M sorbitol (Acros, 132730010). If appropriate, antibiotic selection was performed with nourseothricin at 0.1 g/L for *S. cerevisiae* and *K. phaffii* (Jena Bioscience, AB-101-10ML), geneticin at 0.2 g/L for *S. cerevisiae* and 0.3 g/L for *K. phaffii* (Life Technologies Gibco, 10131-035), and/or hygromycin B at 300 mg/L for *S. cerevisiae* (Thermo Fisher, 10687010). Routine growth conditions were as follows: inoculation in 5 mL media in a 14 mL Falcon tube (Corning, 352059), incubation at 30^∘^, and shaking at 220 rpm or agitation on a rotating drum.

Chemically competent *E. coli* DH5α (NEB, C2987H) was used as a cloning strain and grown in 25 g/L LB Miller broth (10 g/L tryptone + 5 g/L yeast extract + 10 g/L sodium chloride, Fisher Scientific, BP1426-2). Antibiotic selection was performed with 100 mg/L ampicillin (Alfa Aesar, J63807), 25 mg/L chloramphenicol (Alfa Aesar, B20841), or 50 mg/L kanamycin (Alfa Aesar, J61272). Solid media was supplemented with 20 g/L agar (Sunrise Science, 1910-1KG).

### Polymerase chain reaction (PCR)

PCR reactions were performed using the Q5 2X Master Mix (NEB, M0492L). Primers for PCR were designed with Benchling (https://benchling.com/, quality controlled with the New England Biolabs Tm Calculator (https://tmcalculator.neb.com/), and ordered from IDT (Integrated DNA Technologies, Inc., Skokie, Illinois). Reactions were performed in a total volume of 50 μL, with 25 μL of the Q5 Master Mix, 2.5 μL of both the forward and reverse primers (at 10 μM), X μL of template DNA (1 ng plasmid DNA, 100 ng genomic DNA), and 20-X μL of nuclease free water (VWR 02-0201-0500). PCR settings were determined based on instructions from NEB:

1. 98C for 30 sec
2. 30 PCR cycles:
  - 98C for 10 sec
  - annealing temp. for 15 sec
  - 72C for 20 sec per kbp
3. 72C for 2 min
4. 10C hold

### Modular cloning

Modular cloning with TypeIIS restriction enzymes was used to assemble genetic designs. Modular cloning uses a hierarchical assembly process to make parts, transcription units, and pathways. TypeIIS cloning reactions were based on the TypeIIS enzymes BbsI (10 U/μL, Thermo Scientific, ER1011) or BsaI (10 U/μL, NEB, R0535). DNA parts were diluted to 20 fmol/μL, with 1 μL of each part used in the reaction (2 parts for L0 assembly, 4 parts for L1 assembly). 7.9 - N parts (2 or 4) μL of nuclease free water was added to a PCR tube (USA Scientific, 1402-4700). Next, 1 μL of 10X Ligase Buffer and 0.4 μL of 20 U/μL T4 DNA ligase (Promega, M1794) was added to the tube. Finally, 1 1 μL of either the BbsI (L0 assembly) or BsaI (L1 assembly) enzymes was added to the reaction, yielding a total reaction of 10.3 μL. The reaction was then run on a thermocycler with the following conditions: 37 ^∘^C for 5 h, 50 ^∘^C for 15 min, 80 ^∘^C for 20 min, and a hold at 10 ^∘^C.

### Gibson cloning

Gibson assembly reactions followed instructions from the NEBuilder HiFi DNA Assembly Master Mix (NEB, E2621S). Briefly, PCR was used to amplify fragments with overlapping sequences (20–30 bp overlaps). When appropriate, the DpnI enzyme was used to digest template plasmid (NEB, R0176S) per instructions. The fragments were diluted to 0.2 pmols for 2–3 fragments or 0.5 pmols for 4 or more fragments in nuclease-free water, and transferred to a PCR tube. 10 μL of the HiFi master mix was added along with nuclease free water to reach a total reaction volume of 20 μL. The reaction was then run on a thermocycler at 50 ^∘^C for 60 min, followed by a hold at 10 ^∘^C.

### Yeast transformations

*S. cerevisiae* transformations were done based on the lithium acetate method^108^. *S. cerevisiae* cells from a glycerol stock were inoculated in 5 mL of YPD in a 14 mL Falcon tube and shaken overnight on a rotating drum at 30 ^∘^C. In the morning, these cells were used to inoculate 5 mL of fresh YPD to a density of OD = 0.25. The cells were incubated at 30 ^∘^C on a rotating drum until OD = 1.0 (approximately 4 h). The cells were then pelleted at 500 × *g* for 5 min, washed with 2.5 mL of sterile water, and centrifuged again at 500 × *g* for 5 min. The cells were resuspended in 100 μL of 100 mM lithium acetate (TCI, L0191) and transferred to a 1.5 mL microcentrifuge tube (USA Scientific, 1615-5500). The cells were pelleted at 500 × *g* for 30 s, resuspended to a total volume of 50 μL in about 40 μL of 100 mM lithium acetate, and then flicked to mix. The following were then added to the cell mixture: 240 μL PEG 3350 (VWR, 0955), 36 μL 1.0 M lithium acetate, 5 μL boiled salmon sperm DNA (Invitrogen AM9680, 10 μg/μL), and 50 μL transforming DNA. Between each addition to the cell mixture, the microcentrifuge tube was flicked to completely mix. The salmon sperm DNA was prepared by boiling for 5 min on a thermocycler at 100 ^∘^C. The tube was then incubated at 30 ^∘^C for 30 min, followed by the addition of 35 μL of dimethyl sulfoxide (DMSO, Sigma, D8418). The heat shock step followed at 42 ^∘^C for 15 min. For auxotrophic selection, the cells were plated onto CSM knockout agarose plates. For antibiotic selection, the cells were pelleted at 500 × *g* for 30 s followed by removal of the transformation mixture. 1 mL of YPD was used to gently resuspend the cell pellet and transferred to 4 mL of fresh YPD in a Falcon tube. The cells were allowed to incubate overnight at 30 ^∘^C, and then plated onto YPD agarose plates with the appropriate antibiotic. For both auxotrophic and antibiotic selections, the plates were incubated at 30 ^∘^C until transformants appeared (typically 2–4 days).

Transformation of *K. phaffi* was performed by electroporation^28^. A 10 mL preculture in a 100 mL flask was inoculated from a glycerol stock and grown overnight at 30 ^∘^C with shaking at 200 rpm. The next morning, 50 μL of preculture was tranferred into 100 mL fresh YPD in a 250 mL flask, and this culture was incubated overnight again to OD600 = 1.3–1.5. This culture was harvested into three 50 mL conical tubes and pelleted at 4 ^∘^C, 1,500 × *g* for 5 min. The media was decanted and the pellet was resuspended by tapping firmly. The three pellets were resuspended in ice-cold sterile water and combined into one tube to a total of 40 mL. The cells were pelleted again, decanted, and resuspended in 20 mL ice-cold water. The cells were pelleted again and resuspended in 20 mL ice-cold 1 M sorbitol. The cells were pelleted again, the sorbitol was decanted, and the pellet was loosened by tapping firmly. 500 μL of ice-cold sorbitol was added to the pellet and mixed by flicking. These electrocompetent cells were stored on ice. Electroporation cuvettes (2 mm gap, Molecular BioProducts, 5520) were stored on ice during the centrifugation steps, and DNA was added to the bottom (5–10 μg for plasmid DNA, 5–20 μg linearized DNA for integration, or 10 μg circular transfer vector plus 10 μg recombinase expression vector for recombinase-based transformations). 80 μL of competent cells were added to the DNA-containing electroporation cuvettes and incubated on ice for 5 min. Cells were electroporated at 1500 V, then transferred into a round-bottom Falcon tube containing 1 mL 2xYPD at room temperature. Cells were recovered overnight at 30 ^∘^C with shaking at 200 rpm and 100–200 μL was plated onto YPD antibiotic plates. Plates were incubated at 30 ^∘^C until colonies appeared (2–4 days).

Transformation of *Y. lipolytica* was performed by chemical transformation^109^. A 10 mL preculture in a 250 mL flask was inoculated from a glycerol stock and incubated at 30 ^∘^C with shaking at 200 rpm overnight. The next day, 25 mL of fresh YPD was inoculated from the preculture to OD6O00 = 0.5, and incubated for at 30 ^∘^C with shaking. After 3 h, 250 μL of 5 M hydroxyurea (Sigma H8627) was added to the culture, and incubation was continued for another 2 h. The cells were then transferred to a 50 mL conical tube (Greiner bio-one, 227261), centrifuged at 1500 × *g* for 5 min, and washed twice with 10 mL sterile deionized water. The pellet was resuspended to OD600 = 50 in 0.1M lithium acetate. For each transformant, 100 μL was transferred to a 1.5 mL microcentrifuge tube, which was centrifuged at 1500 × *g* for 5 min. The supernatant was removed and the following were added: 90 μL of 50% PEG-3350, 5 μL of 2 M ditriothreitol (G Bioscience, 277D-E), 5 μL of 2 M lithium acetate, and 2.5 μL of sheared, boiled salmon sperm DNA (10 μg per μL). This cocktail was mixed well with the cells by vortexing, then 5–10 μg of plasmid DNA in less than 40 μL was added and mixed by flicking. The cell and DNA mixture was then heat shocked at 39 ^∘^C for 1 h. The entire transformation mixture was plated onto SC media without leucine and incubated at 30 ^∘^C until colonies appeared (4 days).

### Parts and plasmids

All genetic parts used in this study and their sources are detailed in Supplementary Table S10. Parts made for this study were synthesized by Integrated DNA Technologies (IDT). Design included codon optimization using IDT’s proprietary algorithm and elimination of BsaI and BbsI restriction sites.

This study used cloning plasmids, integrating plasmids, and shuttle vectors. Plasmids built for this study are depicted in Supplementary Fig. S3. In modular cloning, plasmids that maintain transcriptional parts are referred to as Level 0 (L0) plasmids and plasmids maintaining transcription units are referred to as Level 1 (L1) plasmids. The L0 plasmids used in this study – pJHC07AB (Supplementary Fig. S3a), pJHC07BC (Supplementary Fig. S3b), pJHC07CD (Supplementary Fig. S3c) – are derived from pEMY07AB, pEMY07BC, and pEMY07CD^51^. These were constructed using Gibson assembly (NEB, E2611S) to replace the lacZ selection gene with the ccdb selection gene^110^. Plasmid pJHC07AB maintains promoters, pJHC07BC maintains ORFs (genes), and pJHC07CD maintains terminators. Integrating L1 plasmids built for this study included pJHC15HR1 (Supplementary Fig. S3d), pJHC15HR2 (Supplementary Fig. S3e), pJHC15HR3 (Supplementary Fig. S3f), pJHC15HR4 (Supplementary Fig. S3g), pJHC15HR5 (Supplementary Fig. S3h), and pJHC15HR6 (Supplementary Fig. S3i). These were constructed using Gibson assembly, and included two connector sequences, the ccdB selection gene, the chlR cassette, and ColE1 replicon. The connector sequences are sequentially homologous 60bp spacers, such that the 3′ spacer of pJHC15HR1 is homologous to the 5′ spacer of pJHC15HR2 and so forth. Once a transcription unit was assembled into these plasmids, PCR was used amplify the transcription unit fragment and the flanking connectors. These fragments were integrated into the *S. cerevisiae* genome using the native homologous recombination pathway, similar to DNA assembler^29^. We targeted two *S. cerevisiae* loci – ChrXV and HO (definitions and ref to supplement). The shuttle vector pCY112 built for this study is depicted in Supplementary Fig. S3j. It was constructed using Gibson assembly, and contains the ccdB selection gene, ColE1 replicon, chlR cassete, the low copy yeast replicon CEN6/ARSH4, and the Klleu2 auxotrophic cassette. Parts and plasmids specific to each strain are described in the next section.

### Yeast strain design and construction

#### FEY_1

The parent strain was *S. cerevisiae* S288C hap1:HAP1^111^. The design was a metabolic pathway for synthesis of valine-derived chemicals (Supplementary Fig. S4a). The sequences for acetolactate synthase(*ahas1*), ketol-acid reductoisomerase (*ilv6*), and dihydroxy-acid dehydratase (*ilvD1*) were derived from *Penicillium chrysogenum*^112^. The sequence for aldehyde decarbonylase (*ado*) was derived from *Prochlorococcus marinus*^113^. The sequence for alpha-ketoisovalerate decarboxylase (*kivD*) was derived from *Lactococcus lactis*^114^. These CDSs were cloned into pJHC07BC using TypeIIS assembly. Transcription units were then built by combining L0 promoter, CDS, and terminator plasmids into a L1 integrating plasmid. The resulting transcription unit plasmids were pJHC15HR1-Ptef1-ahas1-Ttip1, pJHC15HR2-Psmtef1-ilv6-Tprm9, pJHC15HR3-Phta1-ilvD1-Tyhi9, pJHC15HR4-Pagtef1-nat-Tagtef1, pJHC15HR5-Psptdh3-ado-Trpl41b, and pJHC15HR6-Ptdh3-kivD-Trpl15a. Each level 1 vector was linearized by PCR and transformed into *S.cerevisiae* strain S228c, along with homology arms for the ChrXV integration locus (with the 5′ arm containing the spacer homologous to the pJHC15HR1 5′ spacer and 3′ homology arm containing the spacer homologous to the pJHC15HR6 3′ spacer). The primers used to amplify the homology arms from genomic DNA are included in Supplementary Table S10. The linearized fragments then assembled by yeast assembly. Transformants were selected on YPD with nourseothricin and verified by PCR.

#### FEY_2

The parent strain was *S. cerevisiae* CEN.PK113-7D^115^. The design was a metabolic pathway for the synthesis of β-carotene (Supplementary Fig. S4b). The sequences for geranylgeranyl diphosphate synthase (*crtE*), bifunctional lycopene cyclase/phytoene synthase (*crtYB*), and phytoene desaturase (*crtI*) were sourced from a previous study^87^. These coding sequences were synthesized, cloned into L0 pEMY07BC vectors with a BbsI type IIs reaction, assembled into level 1 transcription units (Psbtdh3-crtE-Trpl41b, Psptdh3-crtYB-Tyol036w, Phta2-hyg-Tagtef1, and Psmtef1-crtI-Trpl15a) with BsaI type IIs reactions, and integrated into the ChrXV locus as described above; however, the construct was integrated into strain CEN.PK113-7D and selected on YPD with hygromycin B. Plasmids pAG700 and pAG22-2 are dCas9 and gRNA expression plasmids, respectively, and were provided by Amar Ghodasara. Each plasmid was sequentially transformed into the above-described crt pathway integration strain with selection on YPD with hygromycin B, geneticin, and nourseothricin to yield FEY_2.

#### FEY_5

The parent strain was CEN.PK113-7D. The design was a fluorescent protein integrated into a genomic locus with an antibiotic selection marker (Supplementary Fig. S4c). The sequence for the fluorescent protein encoding gene *yEmCitrine* was sourced from a previous study^92^ and cloned into L0 vector pEMY07BC with a BbsI type IIs reaction. Level 1 transcription units Pact1-yEmCitrine-Tadh1 and Pagtef1-Nat-Tagtef1 were assembled into pJHC15HR1 and pJHC15HR2 with BsaI type IIs reactions and were integrated into the ChrXV locus as described above.

#### FEY_15

The parent strain was CEN.PK113-7D. The design was an inducible deactivated Cas9 expression cassette integrated into a chromosomal locus along with a high-copy replicating vector containing the guide RNA expression cassette (Supplementary Fig. S4d).The deactivated Cas9 (*dCas9Mx1*) sequence was sourced from a previous study^35^ and cloned into pEMY07BC. Transcription units Pgal10-dCas9Mx1-Tspo1 and Psptef1-nat-Ttip1 were assembled in pJHC15HR1 and pJHC15HR2 with BsaI type IIs reactions and integrated into the HO locus (HO locus homology arm primers included in Supplementary Table S10). Yeast shuttle vector pY128 containing the insert Psnr52-gRNAscr-TtracrSUP4 was subsequently transformed into the integration strain to yield FEY_15. The pY128 vector was sourced from a previous study^51^.

#### FEY_18

The parent strain was *S. cerevisiae* strain W303^11^. The design was a Cas9 expression cassette on a low copy number plasmid and a guide RNA expression cassette on a high copy number plasmid (Supplementary Fig. S4e). Plasmids p414-Tef1p-Cas9-Cyc1t and p426-Snr52p-gRNA.CAN1.Y-Sup4t^30^ were purchased from Addgene (43802 and 43803).

#### FEY_27

The parent strain was S288C hap1:HAP1. The design was a Cpf1 expression strain on a low copy number plasmid (Supplementary Fig. S4f). Plasmid pCSN067^38^ was purchased from Addgene (101748).

#### FEY_29

The parent strain was S288C hap1:HAP1. The design was a Cpf1 programming crRNA expression strain on a high copy number plasmid (Supplementary Fig. S4g). Plasmid pUDE722^37^ was purchased from Addgene (103022).

#### FEY_30

The parent strain was S288C hap1:HAP1. The design was a Cre expression strain on a low copy number plasmid (Supplementary Fig. S4h). Plasmid pSH66^33^ was purchased from Euroscarf (P30672).

#### FEY_37

The parent strain was S288C hap1:HAP1. The design was a single enzyme expression strain on a high copy number plasmid (Supplementary Fig. S4i). The sequence for prespatane sesquiterpene synthase (*pst*) was derived from *Laurencia pacifica*^88^. This coding sequence was synthesized, cloned into level 0 vector pEMY07BC with a BbsI type IIS reaction, and cloned with Ppgk1 and Ttdh1 by BsaI type IIS reactions into the level 1 shuttle vector pY128. Shuttle vector pY128 contains the endogenous yeast 2μ plasmid origin of replication.

#### FEY_43

The parent strain was S288C hap1:HAP1. The design was a three enzyme pathway integrated into a chromosomal locus with an antibiotic selection marker (Supplementary Fig. S4j). The sequence for bifunctional diterpene synthase (*mdst*) was derived from *Selaginella moellendorffii*^116^. The sequence for bifunctional ferruginol, 11-hydroxyferruginol synthase (*hfst*) was derived from *Salvia pomifera*^117^. The sequence for 11-hydroxyferruginol C20-oxidase (*cast*) was derived from *Salvia rosmarinus*^89^. These coding sequences were synthesized and cloned into level 0 vector pEMY07BC with BbsI. Transcription units Psktef1-mdst-Tecm10, Psptef1-nat-Ttip1, Psmtdh3-hfst-Ttdh3, and Psmtef1-cast-Teno1 were assembled into pJHC15HR1-4 with BsaI, linearized by PCR, and integrated into the HO locus as described above.

#### FEY_45

The parent strain was S288C hap1:HAP1. The design was a two enzyme pathway integrated into a chromosomal locus with an antibiotic selection marker (Supplementary Fig. S4k). The sequence for dimethylallylcistransferase (*nppst*) was derived from *Solanum lycopersicum*^118^. The sequence for limonene synthase was derived from *Citrus limon*^119^. These coding sequences were synthesized and cloned into level 0 vector pEMY07BC with BbsI. Transcription units Psbtef1-nppst-Tecm10, Psptef1-nat-Ttip1, and Psktdh3-lst-Ttdh1 were assembled into pJHC15HR1-3, linearized by PCR, and integrated into the HO locus as described above.

#### FEY_48

The parent strain was *S. cerevisiae* BY4742^13^. The design was a monocistronic dual fluorescent protein construct integrated into a chromosomal location as well as an empty yeast shuttle vector with a low copy number origin and auxotrophic complementation marker (Supplementary Fig. S4l). The *yEGFP-2A-mRuby* sequence was designed by combining the *yEGFP* and *mRuby* sequences from Sheff et al.^92^ and Lee et al.^93^, respectively, with a self-cleaving 2A sequence^94^. This coding sequence was cloned into level 0 vector pEMY07BC with BbsI. Transcription units Psptef1-yEGFP-2A-mRuby-Trps9a and Psptef1-nat-Ttip1 were assembled into pJHC15HR1-2 with BsaI, linearized by PCR, and integrated into the HO locus of BY4742 as described above. The lacZ*α* insert of pEMY112^51^ was substituted with a ccdB insert to form pCY112, which was then transformed into the EGFP-2A-mRuby integrated strain above. The plasmid pCY112 contains the CEN6/ARSH4 yeast plasmid origin of replication.

#### FEY_55

The parent strain was *S. cerevisiae* BY4741^13^. The design was a low copy number yeast shuttle vector expressing a fluorescent protein (Supplementary Fig. S4m). Plasmid pKK1112(Prev1-Venus-Teno2//LEU2//CEN6//KanR-ColE1) was generated using parts from the MoClo Yeast Toolkit^27^. The following level 0 parts were combined with BsaI into an eight-part level 1 shuttle vector: pYTK084, pYTK002, pYTK027, pYTK033, pYTK055, pYTK067, pYTK075, and pYTK081.

#### FEY_73

The *S. cerevisiae* strain BY4743^120^ was used without modification.

#### FEY_74

The parent strain was *Y.lipolytica* strain Po1f^16^. The design was a plasmid expressing Cas9 (Supplementary Fig. S4n). Plasmid pCRISPRyl^39^ was purchased from Addgene (103022).

#### FEY_75

The parent strain was *K.phaffii* strain CBS 7435 (ATCC 76273). The design was a fluorescent protein expression cassette integrated into a genomic locus by site-specific recombination (Supplementary Fig. S4o). The strain containing an *attP* site for BxbI-mediated recombination was created by transforming plasmid PP74 linearized by AccI (NEB, R0161S) into the parent strain as described by Perez-Pinera^28^. An integrating vector pKK2147(Pgap-aMFnoEAEA-RFPsec-Taox1) was constructed by combining pYTK084, pYTK002, pYTK067, and pYTK078^27^ with pPTK002, pPTK006, pPTK018, pPTK019, and pPTK020 in a 9-part BsaI type IIs reaction as described by Obst^121^. This integration vector was co-transformed with BxBI expression plasmid PP43 into the attP-containing strain and selected on YPD plates with nourseothricin and G418 to yield FEY_75.

### High-molecular weight genomic DNA isolation

Genomic DNA was isolated using Promega’s Genomic DNA Isolation Kit (Promega, A1120). A modified version of Promega’s protocol for yeast gDNA isolation was used to limit shearing of DNA, with added insight from Josh Quick’s Ultra-long read sequencing protocol^122^. No vortexing and limited pipetting/mixing steps were used to maximize Nanopore read lengths. 5 mL of cells were grown overnight (or until saturation) at 30. The cells were pelleted at 500 × *g* for 5 min, and resuspended in 1.5 mL of 50 mM EDTA (Millipore, 324506) and 37.5 μL of 5 U/μL zymolyase (Zymo, E1004). The samples were incubated at 37 °C for 1 h to allow the Zymolyase to digest the cell wall. The cells were pelleted at 500 × *g* for 5 min, re-suspended in 1.5 mL of the Nuclei Lysis Solution (mix by inversion, flicking), and incubated at room temperature for 30 min. 7.5 μL of RNAse A Solution was then added and incubated for 15 min at 37 °C. Once cooled to room temperature, 500 μL of the protein precipitation solution was added (invert to mix). The samples were put on ice for 5 min, and subsequently centrifuged for 10 min at 3000 × *g*. 700 μL of the supernatant was added to a fresh microcentrifuge tube with 700 μL of isopropanol (Sigma, I9516). The microcentrifuge tubes were gently mixed by inversion and centrifuged at 4000 × *g* for 1 min. The DNA pellet was washed with 70% ethanol (Sigma, E7023) and centrifuged at 4000 × *g* for 1 min. The ethanol was carefully pipetted off the DNA pellet, and the tube cap was left open at room temperature for 20 min to allow residual ethanol to evaporate. 50 μL of 10 mM Tris-HCl (Alfa Aesar, J67233) and 0.02% Triton X-100 (Sigma-Aldrich, X100-500ML) was added to resuspend the DNA pellet and incubated overnight at 4 °C. DNA quality was evaluated using a Nanodrop, and the concentration was calculated using a Qubit.

### Nanopore DNA library preparation and MinION loading

The Rapid Barcoding Kit was used to tagment the DNA libraries for sequencing (ONT, SQK-RBK004). Up to four genomes were multiplexed on each MinION flow cell. Library preparation closely followed the protocol provided by ONT. Briefly, 400 ng of template DNA for each isolate was diluted to 7.5 μL, mixed with 2.5 μL of the Fragmentation Mix, and then incubated at 30 ^∘^C for 1 min and 80 ^∘^C for 1 min on a thermal cycler. The barcoded samples were then pooled together and concentrated using AMPure XP beads in 10 μL of 10 mM Tris-HCl, 50 mM NaCl. The pooled sample was next mixed with 1 μL of RAP for 5 min at room temperature, and stored on ice until ready to load. R9.4 MinION Flow Cells (ONT, FLO-MIN106) were used for all sequencing runs. The flow cells were first primed per ONT’s instructions. The 11 μL of prepped DNA was mixed with 4.5 μL of nuclease-free water, 34 uL of SQB, and 25.5 μL of LLB and loaded onto the MinION flow cell. Sequencing runs were executed using ONT’s MinKNOW software (v8.3.1) with the default settings.

### Read processing

Nanopore fast5 files were basecalled using Guppy v2.3.5 (Oxford Nanopore base caller). The subsequent fastq files were demultiplexed using the EPI2ME interface (Metrichor, Oxford, UK). Illumina reads were demultiplexed using the native software on the iSeq machine. Random subsets of Illumina and Nanopore reads at a specific genome coverage were generated using a custom python script (https://github.com/aseetharam/common_scripts/blob/master/sample_fastq.py).

For the metagenome experiment, nanopore and illumina reads from FEY_15 and the zymo mock metagenome were combined into the same nanopore and Illumina fastq file. FEY_15 Nanopore reads at 10X genome coverage were used for each read dilution experiment. The number of zymo mock metagenome nanopore reads was based off the estimated number of base pairs in the FEY_15 read library. The genome size of *S. cerevisiae* is 12.1 Mb, so 10X genome coverage is 121 Mb. Therefore, the nanopore read library sizes of the zymo mock metagenome were 121 Mb (1:1), 1210 Mb (1:10), 12100 Mb (1:100), and 121000 Mb (1:1000). All of the Illumina reads from FEY_15 and the zymo mock metagenome were simply combined into one file and used for each of the four dilution experiments.

### Illumina DNA library preparation and iSeq 100 loading

The Nextera DNA Flex Library Prep Kit (Illumina, 20018704) along with the Nextera DNA CD Indexes (Illumina, 20018707) were used to tagment the DNA libraries for sequencing. Library preparation closely followed the instructions provided by Illumina, and up to four genomes were multiplexed on one Illumina sequencing cartridge. Briefly, tagmentation was first performed with 500 ng of genomic DNA in 30 μL of nuclease-free water. The reaction was stopped by adding 10 μL of both the BLT and TB1 reagents and incubating at 55^∘^C for 15 min on a thermal cycler. Index adapters for each sample along with EPM were then added to barcode and amplify the genomic DNA. The DNA libraries were amplified using the following PCR program:

1. 68C for 3 min
2. 98C for 3 min
3. 5 PCR cycles:
  - 98C for 45 sec
  - 62C for 30 sec
  - 68C for 2 min
4. 68C for 1 min
5. 10C hold

The DNA libraries were cleaned using subsequent steps with the SPM reagent and 80% ethanol, and concentrated in 32 μL of the RSB reagent. Assuming four genomes were multiplexed on one flow cell, 25 pM of each DNA library were pooled together in 100 μL of the RSB reagent and stored on ice until ready to load. The pooled libraries were loaded onto the sequencing cartridges according to Illumina’s instructions. The Local Run Manager on the iSeq 100 machine was used to initiate sequencing runs. A GENERATEFASTQ run was started, and run with the parameters Read Type: Paired End, Read Lengths: 151, and Index Reads: 2.

### Nanopore de novo genome assembly

For the MiniASM^68^ assembly, reads were first mapped using minimap2 (v2.17-r941)^61^ with the parameters "-x ava-ont -t8”. MiniASM (v0.3) was then subsequently run with the default parameters. Canu^69^ (v1.8) was run with the parameters "minReadLength=2500 mhapSensitivity=high corMhapSenstivity=high corOutCoverage=500”. SMARTdenovo^70^ (v1.0) was run using the parameters "-c 1 -k 14 -J 2500 -e zmo”. Flye^71^ (v2.4) was run with the parameters "–meta –plasmids". ABySS^64^ (v 2.1.5) was run with the abyss-pe option and the parameter "k=96". Edena^63^ (v3.131028) was run with the default parameters. Velvet^65^ (v1.2.10) was run with a hashlength of 21 bp. MaSuRCA (v3.3.4) was run with the parameter "JF_SIZE = 242000000 FLYE_ASSEMBLY=1". SPAdes (v3.13.1) was run with the parameters "–sc –nanopore –pe < *#* > -1 –pe < *#* > -2".

### Nanopore and Illumina read polishing

The de novo Nanopore genome assemblies were first polished with Nanopore reads using Medaka (v0.4) with the default parameters. The assembly was then polished with Illumina reads, first with Racon (v1.3.1) followed by Pilon (v1.22). For Racon, the Illumina reads were first mapped to an assembly using minimap2 with the parameter "-ax sr”. Racon^78^ was then run using the default parameters. For Pilon, assemblies were first indexed using bwa (v0.7.17-r1188)^123^. Illumina reads were then mapped to the assembly using bwa with the parameter "mem -t 14”. Pilon^79^ was then run using the parameter "-Xmx160G”.

### Prymetime genome assembly workflow

Visualization of the full Prymetime workflow is shown in Supplementary Fig. S20. First, Flye (v2.4) was run with the parameters "–meta –plasmids" on a Nanopore fastq file to generate the initial genome assembly. Contigs in the resulting assembly file were separated into circular or linear contig files. This was accomplished using a custom python script and the assembly_info.txt file resulting from Flye. The linear contigs were polished first with Medaka (Nanopore reads), followed by Racon and Pilon (Illumina reads). Medaka was run with the default parameters. In preparation for Racon polishing, the Illumina reads were mapped using minimap2 with the parameter "-ax sr". Racon was then run with the default parameters. For Pilon preparation, the assembly was first indexed with bwa, followed by mapping with bwa and the parameter "mem -t 14". Pilon was then run with the parameter "-Xmx160G". To find potential circular contigs that Flye may have missed, a custom python script was used on the polished linear contigs file. The script used the Mummer option nucmer (v3.1)^124^ with the parameters "maxmatch = True, simplify = False, mincluster = 2000, min_id = 99, min_length = 2000, coords_header = True" on contigs that were less than 50,000 bp to identify repetitive contigs. The repetitive contigs were extracted and combined with the circular contigs from the initial Flye assembly, and sent to be re-assembled with Unicycler (v0.4.8). In order to do this, the contigs were first separated into separate fasta files using awk (v4.0.2). Nanopore and Illumina reads were then mapped to each individual contig, with matches extracted into fastq files. The Nanopore reads were mapped using minimap2 with the parameters "-ax map-ont" followed by extraction of hits with samtools (v1.9) and the parameters "fastq -n -F 4 -". Each paired-end Illumina file was mapped using minimap2 with the parameter "-ax sr" followed by extraction of hits with samtools and the parameters "fastq -n -F 4 -". The resulting two Illumina files were paired using fastq_pair (v1.0) with the default parameters^125^. Unicycler was then run with the mapped and paired Illumina files along with the mapped Nanopore file with the default parameters^82^. The re-assembled circular and repetitive contigs resulting from Unicycler were combined with the polished linear contigs, yielding the final assembly.

### Prymetime annotation and visualization of engineering and genome features

Non-native engineering signatures were detected in the genome assemblies using BLASTN (v2.5.0) with the parameters "-perc_identity 98 -qcov_hsp_perc 98". The query for this BLASTN search was a curated list of all non-native engineering signatures used to engineer the yeast strains in this study, and are included in the Prymetime package. The genome features telomeres, centromeres, and mitochrondrion were detected using BLASTN with the parameters "-max_target_seqs 1 -max_hsps 1". The genome feature sequences were downloaded from the Saccharomyces Genome Database^100^, and are included in the Prymetime package. The genome plotter chromoMap (v0.2)^101^ was run with the parameters "data_based_color_map = T, data_type = "categorical" to show the engineering signatures and genome elements hits from the BLASTN search in the context of the entire genome assembly. The genome alignment and visualization software AliTV (v1.0.6) was run with the default parameters^102^.

### Genome assessment tools

QUAST^126^ (v5.0.0) was run with the default parameters, yielding the metrics number of contigs, maximum contig length, and N50. For accuracy-related metrics, the nucmer command was run as part of the MUMmer package^124^. The command "dnadiff -d" was used on the resulting delta file to find the average identity to the reference and the number of SNPs. Genome assemblies were evaluated for genome completeness using BUSCO (v4.0.6)^95^ with the saccharomycetales_odb9 datasets, as well as a BLASTN^40^ search of ORFs from *S. cerevisiae* S288C. Engineered signatures were searched for in-genome assemblies using BLASTN with the expect threshold set at 0.0001.

### Reporting summary

Further information on research design is available in the Nature Research Reporting Summary linked to this article.

## Supplementary information

Supplementary Information

Reporting Summary

## Data Availability

Illumina and nanopore raw reads from all engineered yeast strains have been deposited to DDBJ/ENA/GenBank under the BioProject PRJNA650312. Illumina and nanopore raw reads from the non-engineered yeast strains have been deposited to DDBJ/ENA/GenBank under the BioProject PRJNA694170. All yeast genome assemblies from this study (engineered and non-engineered) are available in https://github.com/emyounglab/prymetime_genomes.

## Code availability

Prymetime can be accessed as a Docker image on GitHub at https://github.com/emyounglab/prymetime. The Prymetime code has been published on Zenodo, 10.5281/zenodo.4440108.

## References

[1] Ostrov N (2019). Technological challenges and milestones for writing genomes. Science.

[2] Bartley BA, Beal J, Karr JR, Strychalski EA (2020). Organizing genome engineering for the gigabase scale. Nat. Commun..

[3] Collins JH, Young EM (2018). Genetic engineering of host organisms for pharmaceutical synthesis. Curr. Opin. Biotech..

[4] Paddon CJ, Keasling JD (2014). Semi-synthetic artemisinin: a model for the use of synthetic biology in pharmaceutical development. Nat. Rev. Microbiol..

[5] Zhou YJ, Kerkhoven EJ, Nielsen J (2018). Barriers and opportunities in bio-based production of hydrocarbons. Nat. Energy.

[6] Peralta-Yahya PP, Zhang F, del Cardayre SB, Keasling JD (2012). Microbial engineering for the production of advanced biofuels. Nature.

[7] Werten MWT, Eggink G, Cohen Stuart MA, de Wolf FA (2019). Production of protein-based polymers in *Pichia pastoris*. Biotechnol. Adv..

[8] Keating KW, Young EM (2019). Synthetic biology for bio-derived structural materials. Curr. Opin. Chem. Eng..

[9] Borodina I, Nielsen J (2014). Advances in metabolic engineering of yeast *Saccharomyces cerevisiae* for production of chemicals. Biotechnol. J..

[10] Ekas H, Deaner M, Alper HS (2019). Recent advancements in fungal-derived fuel and chemical production and commercialization. Curr. Opin. Biotechnol..

[11] Thomas BJ, Rothstein R (1989). Elevated recombination rates in transcriptionally active DNA. Cell.

[12] Sikorski RS, Hieter P (1989). A system of shuttle vectors and yeast host strains designed for efficient manipulation of DNA in *Saccharomyces cerevisiae*. Genetics.

[13] Brachmann CB (1998). Designer deletion strains derived from *Saccharomyces cerevisiae* S288C: a useful set of strains and plasmids for PCR-mediated gene disruption and other applications. Yeast.

[14] Markham KA, Alper HS (2018). Synthetic biology expands the industrial potential of *Yarrowia lipolytica*. Trends Biotechnol..

[15] Abdel-Mawgoud AM (2018). Metabolic engineering in the host *Yarrowia lipolytica*. Metab. Eng..

[16] Madzak C, Treton B, Blanchin-Roland S (2000). Strong hybrid promoters and integrative expression/secretion vectors for quasi-constitutive expression of heterologous proteins in the yeast *Yarrowia lipolytica*. J. Mol. Microbiol. Biotechnol..

[17] Peña DA, Gasser B, Zanghellini J, Steiger MG, Mattanovich D (2018). Metabolic engineering of *Pichia pastoris*. Metab. Eng..

[18] Gasser, B. & Mattanovich, D. A yeast for all seasons – is *Pichia pastoris* a suitable chassis organism for future bioproduction? *FEMS Microbiol. Lett.* **365**, 10.1093/femsle/fny181 Fny181, http://oup.prod.sis.lan/femsle/article-pdf/365/17/fny181/25431392/fny181.pdf (2018). 10.1093/femsle/fny181 30052876

[19] Anton BP, Fomenkov A, Raleigh EA, Berkmen M (2016). Complete genome sequence of the engineered *Escherichia coli* shuffle strains and their wild-type parents. Genome Announc..

[20] Solis-Escalante, D. et al. The genome sequence of the popular hexose-transport-deficient *Saccharomyces cerevisiae* strain EBY.VW4000 reveals LoxP/Cre-induced translocations and gene loss. *FEMS Yeast Res.* **15**, 10.1093/femsyr/fou004 (2015). 10.1093/femsyr/fou004 25673752

[21] Li J (2019). Whole genome sequencing reveals rare off-target mutations and considerable inherent genetic or/and somaclonal variations in CRISPR/Cas9-edited cotton plants. Plant Biotechnol. J..

[22] Veres A (2014). Low incidence of off-target mutations in individual CRISPR-Cas9 and TALEN targeted human stem cell clones detected by whole-genome sequencing. Cell Stem Cell.

[23] Schwarzhans J-P (2016). Non-canonical integration events in *Pichia pastoris* encountered during standard transformation analysed with genome sequencing. Sci. Rep..

[24] Baker M (2016). 1500 scientists lift the lid on reproducibility. Nature.

[25] Mumberg D, Müller R, Funk M (1995). Yeast vectors for the controlled expression of heterologous proteins in different genetic backgrounds. Gene.

[26] Voth WP, Richards JD, Shaw JM, Stillman DJ (2001). Yeast vectors for integration at the HO locus. Nucleic Acids Res..

[27] Lee ME, DeLoache WC, Cervantes B, Dueber JE (2015). A highly characterized yeast toolkit for modular, multipart assembly. ACS Synth. Biol..

[28] Perez-Pinera P (2016). Synthetic biology and microbioreactor platforms for programmable production of biologics at the point-of-care. Nat. Commun..

[29] Shao Z, Zhao H, Zhao H (2008). DNA assembler, an in vivo genetic method for rapid construction of biochemical pathways. Nucleic Acids Res..

[30] DiCarlo JE (2013). Yeast oligo-mediated genome engineering (YOGE). ACS Synth. Biol..

[31] Si T (2017). Automated multiplex genome-scale engineering in yeast. Nat. Commun..

[32] Luo J, Sun X, Cormack JD, Brendan P, Boeke M (2018). Karyotype engineering by chromosome fusion leads to reproductive isolation in yeast. Nature.

[33] Hegemann, J. H. & Heick, S. B. *Delete and Repeat: a Comprehensive Toolkit for Sequential Gene Knockout in the Budding Yeast Saccharomyces cerevisiae* (Humana Press, Totowa, NJ, 2011). 10.1007/978-1-61779-197-0_12 21815094

[34] DiCarlo JE (2013). Genome engineering in *Saccharomyces cerevisiae* using CRISPR-Cas systems. Nucleic Acids Res..

[35] Farzadfard F, Perli SD, Lu TK (2013). Tunable and multifunctional eukaryotic transcription factors based on CRISPR/Cas. ACS Synth. Biol..

[36] Ronda C (2015). CrEdit: CRISPR mediated multi-loci gene integration in *Saccharomyces cerevisiae*. Microb. Cell Factories.

[37] Świat MA (2017). FnCpf1: a novel and efficient genome editing tool for *Saccharomyces cerevisiae*. Nucleic Acids Res..

[38] Verwaal R, Buiting-Wiessenhaan N, Dalhuijsen S, Roubos JA (2018). CRISPR/Cpf1 enables fast and simple genome editing of *Saccharomyces cerevisiae*. Yeast.

[39] Schwartz CM, Hussain MS, Blenner M, Wheeldon I (2016). Synthetic RNA polymerase III promoters facilitate high-efficiency CRISPR-Cas9-mediated genome editing in *Yarrowia lipolytica*. ACS Synth. Biol..

[40] Johnson M (2008). NCBI BLAST: a better web interface. Nucleic Acids Res..

[41] de Toro M, Garcilláon-Barcia MP, De La Cruz F (2014). Plasmid diversity and adaptation analyzed by massive sequencing of *Escherichia coli* plasmids. Microbiol. Spectr..

[42] Arredondo-Alonso S, Willems RJ, van Schaik W, Schürch AC (2017). On the (im)possibility of reconstructing plasmids from whole-genome short-read sequencing data. Microb. Genom..

[43] Blount BA (2018). Rapid host strain improvement by in vivo rearrangement of a synthetic yeast chromosome. Nat. Commun..

[44] Gowers G-OF (2020). Improved betulinic acid biosynthesis using synthetic yeast chromosome recombination and semi-automated rapid LC-MS screening. Nat. Commun..

[45] Galanie S, Thodey K, Trenchard IJ, Filsinger Interrante M, Smolke CD (2015). Complete biosynthesis of opioids in yeast. Science.

[46] Meadows AL (2016). Rewriting yeast central carbon metabolism for industrial isoprenoid production. Nature.

[47] Dragosits M, Mattanovich D (2013). Adaptive laboratory evolution – principles and applications for biotechnology. Microb. Cell Factories.

[48] Mans R, Daran J-MG, Pronk JT (2018). Under pressure: evolutionary engineering of yeast strains for improved performance in fuels and chemicals production. Curr. Opin. Biotechnol..

[49] Strucko T (2018). Laboratory evolution reveals regulatory and metabolic trade-offs of glycerol utilization in *Saccharomyces cerevisiae*. Metab. Eng..

[50] Burén S (2017). Formation of nitrogenase NifDK tetramers in the mitochondria of *Saccharomyces cerevisiae*. ACS Synth. Biol..

[51] Young EM (2018). Iterative algorithm-guided design of massive strain libraries, applied to itaconic acid production in yeast. Metab. Eng..

[52] Casini A (2018). A pressure test to make 10 molecules in 90 days: external evaluation of methods to engineer biology. J. Am. Chem. Soc..

[53] Denby CM (2018). Industrial brewing yeast engineered for the production of primary flavor determinants in hopped beer. Nat. Commun..

[54] Awan AR (2017). Biosynthesis of the antibiotic nonribosomal peptide penicillin in baker’s yeast. Nat. Commun..

[55] Ali N, Rampazzo RdCP, Costa ADT, Krieger MA (2017). Current nucleic acid extraction methods and their implications to point-of-care diagnostics. BioMed Res. Int..

[56] van Dijk EL, Jaszczyszyn Y, Naquin D, Thermes C (2018). The third revolution in sequencing technology. Trends Genet..

[57] Watson M, Warr A (2019). Errors in long-read assemblies can critically affect protein prediction. Nat. Biotechnol..

[58] van Dijk EL, Jaszczyszyn Y, Thermes C (2014). Library preparation methods for next-generation sequencing: tone down the bias. Exp. Cell Res..

[59] Wajid B, Serpedin E (2012). Review of general algorithmic features for genome assemblers for next generation sequencers. Genom. Proteom. Bioinform..

[60] Yandell M, Ence D (2012). A beginner’s guide to eukaryotic genome annotation. Nat. Rev. Genet..

[61] Li H (2018). Minimap2: pairwise alignment for nucleotide sequences. Bioinformatics.

[62] Christianson TW, Sikorski RS, Dante M, Shero JH, Hieter P (1992). Multifunctional yeast high-copy-number shuttle vectors. Gene.

[63] Hernandez D, François P, Farinelli L, øOsterås M, Schrenzel J (2008). *De novo* bacterial genome sequencing: millions of very short reads assembled on a desktop computer. Genome Res..

[64] Simpson JT (2009). ABySS: a parallel assembler for short read sequence data. Genome Res..

[65] Zerbino DR, Birney E (2008). Velvet: algorithms for de novo short read assembly using de Bruijn graphs. Genome Res..

[66] Zimin AV (2013). The MaSuRCA genome assembler. Bioinformatics.

[67] Antipov D, Korobeynikov A, McLean JS, Pevzner PA (2015). hybridSPAdes: an algorithm for hybrid assembly of short and long reads. Bioinformatics.

[68] Li H (2016). Minimap and miniasm: fast mapping and de novo assembly for noisy long sequences. Bioinformatics.

[69] Koren S (2017). Canu: scalable and accurate long-read assembly via adaptive k-mer weighting and repeat separation. Genome Res..

[70] Ruan, J. Ultra-fast de novo assembler using long noisy reads. *GitHub* https://github.com/ruanjue/smartdenovo (2018). 10.46471/gigabyte.15 PMC9632051 36824332

[71] Kolmogorov M, Yuan J, Lin Y, Pevzner PA (2019). Assembly of long, error-prone reads using repeat graphs. Nat. Biotechnol..

[72] Giordano F (2017). De novo yeast genome assemblies from MinION, PacBio and MiSeq platforms. Sci. Rep..

[73] Salazar, A. N. et al. Nanopore sequencing enables near-complete de novo assembly of *Saccharomyces cerevisiae* reference strain CEN.PK113-7D. *FEMS Yeast Res.* **17**, 10.1093/femsyr/fox074 (2017). 10.1093/femsyr/fox074 PMC5812507 28961779

[74] Jenjaroenpun P (2018). Complete genomic and transcriptional landscape analysis using third-generation sequencing: a case study of *Saccharomyces cerevisiae* CEN.PK113-7D. Nucleic Acids Res..

[75] Love, K. R. et al. Comparative genomics and transcriptomics of *Pichia pastoris*. *BMC Genom.* 10.1186/s12864-016-2876-y (2016). 10.1186/s12864-016-2876-y PMC4974788 27495311

[76] McIlwain SJ (2016). Genome sequence and analysis of a stress-tolerant, wild-derived strain of *Saccharomyces cerevisiae* used in biofuels research. G3.

[77] ONT. Medaka: sequence correction provided by ONT Research. *GitHub* https://github.com/nanoporetech/medaka (2018).

[78] Vaser R, Sovic I, Nagarajan N, Sikic M (2017). Fast and accurate de novo genome assembly from long uncorrected reads. Genome Res..

[79] Walker BJ (2014). Pilon: an integrated tool for comprehensive microbial variant detection and genome assembly improvement. PLoS One.

[80] Li Z (2012). Comparison of the two major classes of assembly algorithms: overlap-layout-consensus and de Bruijn graph. Brief. Funct. Genom..

[81] Miller JR (2008). Aggressive assembly of pyrosequencing reads with mates. Bioinformatics.

[82] Wick RR, Judd LM, Gorrie CL, Holt KE (2017). Unicycler: resolving bacterial genome assemblies from short and long sequencing reads. PLoS Comput. Biol..

[83] Valli, M. et al. Curation of the genome annotation of *Pichia pastoris* (*Komagataella phaffii*) CBS7435 from gene level to protein function. *FEMS Yeast Res.* 10.1093/femsyr/fow051 (2016). 10.1093/femsyr/fow051 27388471

[84] Kuberl A (2011). High-quality genome sequence of *Pichia pastoris* CBS7435. J. Biotechnol..

[85] Liu, L. & Alper, H. S. Draft genome sequence of the oleaginous yeast *Yarrowia lipolytica* PO1f, a commonly used metabolic engineering host. *Genome Announc.*10.1128/genomeA.00652-14 (2014). 10.1128/genomeA.00652-14 PMC4081999 24994799

[86] Zhang L, Liang Y, Wu W, Tan X, Lu X (2016). Microbial synthesis of propane by engineering valine pathway and aldehyde-deformylating oxygenase. Biotechnol. Biofuels.

[87] Verwaal R (2007). High-level production of beta-carotene in *Saccharomyces cerevisiae* by successive transformation with carotenogenic genes from *Xanthophyllomyces dendrorhous*. Appl. Environ. Microbiol..

[88] Kersten RD (2017). A red algal bourbonane sesquiterpene synthase defined by microgram-scale NMR-coupled crystalline sponge X-ray diffraction analysis. J. Am. Chem. Soc..

[89] Scheler U (2016). Elucidation of the biosynthesis of carnosic acid and its reconstitution in yeast. Nat. Commun..

[90] Cao X (2016). Metabolic engineering of oleaginous yeast *Yarrowia lipolytica* for limonene overproduction. Biotechnol. Biofuels.

[91] Jongedijk E (2015). Capturing of the monoterpene olefin limonene produced in *Saccharomyces cerevisiae*. Yeast.

[92] Sheff MA, Thorn KS (2004). Optimized cassettes for fluorescent protein tagging in *Saccharomyces cerevisiae*. Yeast.

[93] Lee S, Lim WA, Thorn KS (2013). Improved blue, green, and red fluorescent protein tagging vectors for *Saccharomyces cerevisiae*. PLOS ONE.

[94] Souza-Moreira, T. M. et al. Screening of 2A peptides for polycistronic gene expression in yeast. *FEMS Yeast Res.* 10.1093/femsyr/foy036 Foy036, https://academic.oup.com/femsyr/article-pdf/18/5/foy036/24968970/foy036.pdf (2018). 10.1093/femsyr/foy036 29617770

[95] Simao FA, Waterhouse RM, Ioannidis P, Kriventseva EV, Zdobnov EM (2015). BUSCO: assessing genome assembly and annotation completeness with single-copy orthologs. Bioinformatics.

[96] Darling AE, Mau B, Perna NT (2010). progressiveMauve: multiple genome alignment with gene gain, loss and rearrangement. PLOS ONE.

[97] Crosato, G. et al. The impact of CUP1 gene copy-number and XVI-VIII/XV-XVI translocations on copper and sulfite tolerance in vineyard *Saccharomyces cerevisiae* strain populations. *FEMS Yeast Res.* 10.1093/femsyr/foaa028 Foaa028, https://academic.oup.com/femsyr/article-pdf/20/4/foaa028/33336149/foaa028.pdf (2020). 10.1093/femsyr/foaa028 32436567

[98] Kobayashi T (2011). Regulation of ribosomal RNA gene copy number and its role in modulating genome integrity and evolutionary adaptability in yeast. Cell. Mol. Life Sci..

[99] Tøorresen OK (2019). Tandem repeats lead to sequence assembly errors and impose multi-level challenges for genome and protein databases. Nucleic Acids Res..

[100] Cherry JM (2011). Saccharomyces genome database: the genomics resource of budding yeast. Nucleic Acids Res..

[101] Anand, L. & Rodriguez Lopez, C. M. chromoMap: an R package for interactive visualization and annotation of chromosomes. *bioRxiv* 10.1101/605600https://www.biorxiv.org/content/early/2020/01/23/605600.full.pdf (2020). 10.1186/s12859-021-04556-z PMC8753883 35016614

[102] Ankenbrand MJ, Hohlfeld S, Hackl T, Förster F (2017). AliTV–interactive visualization of whole genome comparisons. PeerJ Comput. Sci..

[103] Harris, R. S. *Improved Pairwise Alignment of Genomic DNA* (Pennsylvania State University, 2007).

[104] Nijkamp JF (2012). De novo sequencing, assembly and analysis of the genome of the laboratory strain *Saccharomyces cerevisiae* CEN.PK113-7D, a model for modern industrial biotechnology. Microb. Cell Factories.

[105] Nicholls, S. M., Quick, J. C., Tang, S. & Loman, N. J. Ultra-deep, long-read nanopore sequencing of mock microbial community standards. *GigaScience* 10.1093/gigascience/giz043 (2019). 10.1093/gigascience/giz043 PMC6520541 31089679

[106] Berlin K (2015). Assembling large genomes with single-molecule sequencing and locality-sensitive hashing. Nat. Biotechnol..

[107] Kobayashi T, Heck DJ, Nomura M, Horiuchi T (1998). Expansion and contraction of ribosomal DNA repeats in *Saccharomyces cerevisiae*: requirement of replication fork blocking (Fob1) protein and the role of RNA polymerase I. Genes Dev..

[108] Gietz RD, Schiestl RH (2007). High-efficiency yeast transformation using the LiAc/SS carrier DNA/PEG method. Nat. Protoc..

[109] Jang I-S, Yu BJ, Jang JY, Jegal J, Lee JY (2018). Improving the efficiency of homologous recombination by chemical and biological approaches in *Yarrowia lipolytica*. PLOS ONE.

[110] Bernard P, Gabarit P, Bahassi EM, Couturier M (1994). Positive-selection vectors using the F plasmid ccdB killer gene. Gene.

[111] Hickman MJ, Winston F (2007). Heme levels switch the function of Hap1 of *Saccharomyces cerevisiae* between transcriptional activator and transcriptional repressor. Mol. Cell. Biol..

[112] van den Berg MA (2008). Genome sequencing and analysis of the filamentous fungus *Penicillium chrysogenum*. Nat. Biotechnol..

[113] Rocap G (2003). Genome divergence in two *Prochlorococcus* ecotypes reflects oceanic niche differentiation. Nature.

[114] Bolotin A (2001). The complete genome sequence of the lactic acid bacterium *Lactococcus lactis* ssp. lactis IL1403. Genome Res..

[115] Entian, K.-D. & Kötter, P. In *Methods in Microbiology* Vol. 36 (eds. Stansfield, I. & Stark, M. J.) (Academic Press, 2007).

[116] Sugai Y (2011). Enzymatic 13C labeling and multidimensional NMR analysis of miltiradiene synthesized by bifunctional diterpene cyclase in *Selaginella moellendorffii*. J. Biol. Chem..

[117] Ignea C (2016). Carnosic acid biosynthesis elucidated by a synthetic biology platform. Proc. Natl Acad. Sci. USA.

[118] Schilmiller AL (2009). Monoterpenes in the glandular trichomes of tomato are synthesized from a neryl diphosphate precursor rather than geranyl diphosphate. Proc. Natl Acad. Sci. USA.

[119] Lücker J (2002). Monoterpene biosynthesis in lemon (*Citrus limon*). Eur. J. Biochem..

[120] Giaever G (2002). Functional profiling of the *Saccharomyces cerevisiae* genome. Nature.

[121] Obst U, Lu TK, Sieber V (2017). A modular toolkit for generating *Pichia pastoris* secretion libraries. ACS Synth. Biol..

[122] Jain M (2018). Nanopore sequencing and assembly of a human genome with ultra-long reads. Nat. Biotechnol..

[123] Li, H. Aligning sequence reads, clone sequences and assembly contigs with BWA-MEM. Preprint at http://arXiv.org/1303.3997v2 (2013). 1303.3997.

[124] Kurtz S (2004). Versatile and open software for comparing large genomes. Genome Biol..

[125] Edwards, R. & Edwards, J. A. fastq-pair: efficient synchronization of paired-end fastq files. *bioRxiv* 10.1101/552885 (2019).

[126] Gurevich A, Saveliev V, Vyahhi N, Tesler G (2013). QUAST: quality assessment tool for genome assemblies. Bioinformatics.

