# Supplementary material for: Engineered yeast genomes accurately assembled from pure and mixed samples
Source: Nat Commun. 2021 Mar 5;12:1485. doi: 10.1038/s41467-021-21656-9 (PMC7935868; doi:10.1038/s41467-021-21656-9)
Supplement: Supplementary file 1 — Supplementary Information [file 41467_2021_21656_MOESM1_ESM.pdf]

Supplemental Information for

Engineered yeast genomes accurately assembled from pure and mixed samples

Joseph H. Collins, Kevin W. Keating, Trent R. Jones, Shravani Balaji, Celeste B. Marsan, Marina Çomo, Zachary J. Newlon, Tom Mitchell, Bryan Bartley, Aaron Adler, Nicholas Roehner, Eric M. Young

Table of Contents

Supplementary Table S1: Engineered strains used in this study

Supplementary Table S2: BLASTN results for all engineered signatures in their respective genome assemblies

Supplementary Table S3: Percent unmapped reads for all sequencing runs conducted in this study

Supplementary Table S4: Copy number of rDNA repeats found in chromosomal or extra-chromosomal contigs

Supplementary Table S5: Assembly accuracy using different polishing tools

Supplementary Table S6: Assembly accuracy with polishing at various genome coverage depths of Illumina reads

Supplementary Table S7: BUSCO score for each Nanopore *de novo* assembler, genome coverage depth, and polishing step

Supplementary Table S8: Percent of *S. cerevisiae* S288C CDSs found in each assembly

Supplementary Table S9: Evaluation of *de novo* Nanopore assemblers with structure-related metrics

Supplementary Table S10: All genetic parts used in this study

Supplementary Figure S1: Read length distribution for two Nanopore genomic DNA library preparation kits

Supplementary Figure S2: Improvement to FEY_2 plasmid assembly using Unicycler

Supplementary Figure S3: Plasmid maps used for cloning in this study

Supplementary Figure S4: Design of all engineered yeast strains

Supplementary Figure S5: Comparison of engineered strain assembly plasmids with or without Unicycler

Supplementary Figure S6: Mauve whole genome alignment of all *S. cerevisiae* strains in this study

Supplementary Figure S7: Mauve whole genome alignment of all nonconventional yeasts in this study

Supplementary Figure S8: Resequenced nonconventional yeast genomes compared to reference

Supplementary Figure S9: Chromosome completeness assessment of the engineered *S. cerevisiae* strains

Supplementary Figure S10: Chromomap and AliTV alignments for S288C-derived engineered yeast strains, part 1

Supplementary Figure S11: Chromomap and AliTV alignments for S288C-derived engineered yeast strains, part 2

Supplementary Figure S12: Chromomap and AliTV alignments for CEN.PK-derived engineered yeast strains

Supplementary Figure S13: Chromomap and AliTV alignments for W303α-derived engineered yeast strains

Supplementary Figure S14: Chromomap and AliTV alignments for BY4742-derived engineered yeast strains

Supplementary Figure S15: Chromomap and AliTV alignments for BY4741-derived engineered yeast strains

Supplementary Figure S16: Chromomap and AliTV alignments for *Y. lipolytica*-derived engineered yeast strains

Supplementary Figure S17: Chromomap and AliTV alignments for *K. phaffii*-derived engineered yeast strains

Supplementary Figure S18: Step-by-step validation of the Prymetime workflow with publicly available CEN.PK-113 reads

Supplementary Figure S19: chromoMap visualization of genome structure elements for four nanopore *de novo* assemblers compared to the S288C reference assembly

Supplementary Figure S20: Detailed illustration of the Prymetime workflow

Supplementary Tables

Supplementary Table 1. Engineered strains used in this study

Strain	Parent	Parent Source	Knockout(s)	Chromosome Insert	Plasmid 1	Plasmid 2	Plasmid Source
FEY_1	*S.c.* S288C	Hickman, 2007	-	hap1:HAP1 ChrXV::(Ptef1-ahas1-Ttip1//Psmtef1-ilv6-Tprm9//Phta1-ilvD1-Tyhi9//Pagtef1-nat-Tagtef1//Psptdh3-ado-Trpl41b//Ptdh3-kivD-	-	-	-
FEY_2	*S.c.* CENPK	Entian, 2007	-	ChrXV::(Psbtdh3-crtE-Trpl41b//Psptdh3-crtYB-Tyol036w//Phta2-hyg-Tagtef1//Psmtef1-crtI-Trpl15a)	pAG700::(Ptdh3-dCas9-Tadh1//Pagtef1-nat-Tagtef1//CEN6/ARSH4//AmpR//ColE1)	pAG_22-2::(Pscsnr52-22_2-Tsup4//Pagtef1-kan-Tagtef1//2u//CmR//ColE1)	This study
FEY_5	*S.c.* CENPK	Entian, 2007	-	ChrXV::(Pact1-yEmCitrine-Tadh1//Pagtef1-Nat-Tagtef1)	-	-	-
FEY_15	*S.c.* CENPK	Entian, 2007	-	HO::(Pgal10-dCas9Mx1-Tspo1//Psptef1-nat-Ttip1)	pY128:(Psnr52-gRNAscr-TtracrSUP4//Pagtef1-kan-Tagtef1//2micron//CmR//ColE1)	-	This study
FEY_18	*S.c.* W303	Thomas, 1989	ade2-1 ura3-1 his3-11 trp1-1 leu2-3 leu2-	-	p414-Ptef1-Cas9-Tcyc1	p426-Psnr52-gRNA_CAN1-Tsup4	DiCarlo, 2013, Addgene, Plasmids #43802 & #43803
FEY_27	*S.c.* S288C	Hickman, 2007	-	hap1:HAP1	pCSN067::(LbCpf1-SV40//Ptef1-kan-Ttef1//Ptrp1-TRP1//CEN6/ARSH4//AmpR//ColE1)	-	Verwaal, 2018, Addgene, Plasmid #101748
FEY_29	*S.c.* S288C	Hickman, 2007	-	hap1:HAP1	pUDE722::(Psnr52-Cpf1can1-Tsup4//Ptef1-kan-Ttef1//2u//AmpR//ColE1)	-	Swiat, 2017, Addgene, Plasmid #103022
FEY_30	*S.c.* S288C	Hickman, 2007	-	hap1:HAP1	pSH66::(Pgal1-Cre-Tcyc1//Pagtef1-nat-Tagtef1//CEN6/ARSH4//AmpR//ColE1	-	Hegemann, 2011, Euroscarf, P30672
FEY_37	*S.c.* S288C	Hickman, 2007	-	hap1:HAP1	pY128:(Ppgk1-pst-Ttdh1//Pagtef1-kan-Tagtef1//2micron//CmR//ColE1)	-	This study
FEY_43	*S.c.* S288C	Hickman, 2007	-	hap1:HAP1 HO::(Psktef1-mdst-Tecm10//Psptef1-nat-Ttip1//Psmtdh3-hfst-Ttdh3//Psmtef1-cast-Teno1)	-	-	-
FEY_45	*S.c.* S288C	Hickman, 2007	-	hap1:HAP1 HO::(Psbtef1-nppst-Tecm10//Psptef1-nat-Ttip1//Psktdh3-lst-Ttdh1)	-	-	-
FEY_48	*S.c.* BY4742	Brachmann, 1998	his3Δ0 leu2Δ0 lys2Δ0 ura3Δ0	HO::(Psptef1-yEGFP-2A-mRuby-Trps9a//Psptef1-nat-Ttip1)	pCY112::(ccdb//Pagtef1-Klleu2-Tagtef1//CEN6/ARSH4//CmR//ColE1)	-	This study
FEY_55	*S.c.* BY4741	Brachmann, 1998	his3Δ0 leu2Δ0 met15Δ0 ura3Δ0	-	pKK1112:(Prev1-Venus-Teno2//LEU2//CEN6//KanR-ColE1)	-	This study
FEY_73	*S.c.* BY4743	Giaever, 2002	MATa/α his3Δ1/his3Δ1 leu2Δ0/leu2Δ0 LYS2/lys2Δ0 met15Δ0/MET15	-	-	-	-
FEY_74	Y.l. Po1f	Madzak, 2000	MATA ura3302 leu2270 xpr2322 axp2deltaNU4	-	pCRISPRyl(CEN//UAS1B8-TEF(136)-ylCas9-NLS-Tcyc1//ColE1//AmpR//sgRNA//Leu2)	-	Schwartz, 2015, Addgene, Plasmid # 70007
FEY_75	*K.p.* ATCC 76273	Wegner, 1986	-	trp2::(Kan//pUC//attL//Pgap-aMFnoEAEA-RFP-Taox1/Nat/ColE1/Kan//attR)	-	-	Obst, 2017, and this study

Supplementary Table 2. BLASTN results for all engineered signatures in their respective genome assemblies

Chromosome integrations

Assembly	Percent Identity	# of Gaps	Query Cover
FEY_1	99.98	1	100
FEY_2	99.99	0	100
FEY_5	99.76	6	100
FEY_15	99.97	1	100
FEY_18	-	-	-
FEY_27	-	-	-
FEY_29	-	-	-
FEY_30	-	-	-
FEY_37	-	-	-
FEY_43	99.97	2	100
FEY_45	99.91	3	100
FEY_48	99.95	1	100
FEY_55	-	-	-

Plasmid 1

Assembly	Percent Identity	# of Gaps	Query Cover	Length	Correct Length
FEY_1	-	-	-	-	-
FEY_2	98.73	37	100	6143	6181
FEY_5	-	-	-	-	-
FEY_15	99.8	9	100	4811	4820
FEY_18	98.46	97	100	10984	9524
FEY_27	99.93	2	100	11326	11322
FEY_29	100	0	100	5058	5058
FEY_30	99.88	1	100	7103	7108
FEY_37	99.87	7	100	6289	6296
FEY_43	-	-	-	-	-
FEY_45	-	-	-	-	-
FEY_48	99.98	0	100	4746	4746
FEY_55	100	0	100	6239	6239

Plasmid 2

Assembly	Percent Identity	# of Gaps	Query Cover	Length	Correct Length
FEY_1	-	-	-	-	-
FEY_2	99.78	5	100	9654	9495
FEY_5	-	-	-	-	-
FEY_15	-	-	-	-	-
FEY_18	99.65	55	100	6263	6274
FEY_27	-	-	-	-	-
FEY_29	-	-	-	-	-
FEY_30	-	-	-	-	-
FEY_37	-	-	-	-	-
FEY_43	-	-	-	-	-
FEY_45	-	-	-	-	-
FEY_48	-	-	-	-	-
FEY_55	-	-	-	-	-

Supplementary Table 3. Percentage of unmapped reads for all Nanopore and Illumina sequencing runs conducted in this study.

	Nanopore			Illumina		
Assembly	# of mapped reads	Total # of reads	% unmapped	# of mapped reads	Total # of reads	% unmapped
FEY_1	697,706	699,869	0.31	2,753,181	2,782,839	1.07
FEY_2	125,390	126,276	0.70	5,218,038	5,297,074	1.49
FEY_5	42,803	43,860	2.41	1,772,663	1,799,638	1.50
FEY_15	1,080,000	1,084,770	0.44	1,844,674	1,859,349	0.79
FEY_18	226,416	230,538	1.79	1,565,427	1,584,568	1.21
FEY_27	372,988	377,195	1.12	1,689,817	1,712,861	1.35
FEY_29	24,895	25,289	1.56	2,405,033	2,432,880	1.14
FEY_30	290,290	292,686	0.82	1,712,745	1,728,205	0.89
FEY_37	82,571	83,461	1.07	2,818,802	2,849,926	1.09
FEY_45	30,649	31,127	1.54	2,558,221	2,594,864	1.41
FEY_48	135,575	138,358	2.01	2,845,218	2,903,080	1.99
FEY_73	177,309	177,721	0.23	981,633	993,615	1.21
FEY_74	13,336	14,761	9.65	2,984,430	3,010,418	0.86
FEY_75	233,165	235,752	1.10	4,938,763	4,988,155	0.99
YL Po1f	607,370	661,061	8.12	1,680,619	1,695,330	0.87
KP CBS7435	358,514	359,921	0.39	1,968,607	2,002,731	1.70

Supplementary Table 4. Copy number of rDNA repeats found in chromosomal or extra-chromosomal contigs.

Strain	Chr copy #	Extra-chr copy #
FEY_1	0	1
FEY_2	0	2
FEY_5	0	3
FEY_15	0	3
FEY_18	0	5
FEY_27	0	1
FEY_29	0	1
FEY_30	0	1
FEY_37	0	1
FEY_43	0	1
FEY_45	2	0
FEY_48	0	1
FEY_55	0	1
BY4743	0	1
CEN.PK113-7D	0	3
S288C	2	0

Supplementary Table 5. Improvement to assembly accuracy using different polishing tools.

Assembler	Raw	Medaka	Racon	Pilon
MiniASM	86.77	99.41	99.89	99.90
Canu	99.33	99.42	99.90	99.91
Flye	98.16	99.41	99.90	99.90
SMARTdenovo	99.13	99.39	99.89	99.89

Supplementary Table 6. Improvement to assembly accuracy through polishing at various genome coverage depths of Illumina reads.

Coverage	Assembler	Avg ID	# of SNPs
0X	MiniASM	99.41	3629
	Canu	99.42	3714
	Flye	99.41	3639
	SMARTdenovo	99.39	3589
5X	MiniASM	99.56	16824
	Canu	99.56	16879
	Flye	99.56	16915
	SMARTdenovo	99.54	16749
10X	MiniASM	99.68	14149
	Canu	99.69	14141
	Flye	99.69	14202
	SMARTdenovo	99.68	13848
20X	MiniASM	99.82	6361
	Canu	99.83	6281
	Flye	99.83	6340
	SMARTdenovo	99.80	6277
40X	MiniASM	99.89	1944
	Canu	99.89	1837
	Flye	99.89	1871
	SMARTdenovo	99.87	1813
100X	MiniASM	99.89	1137
	Canu	99.90	1036
	Flye	99.90	1139
	SMARTdenovo	99.89	1079
200X	MiniASM	99.90	1027
	Canu	99.91	1027
	Flye	99.90	1081
	SMARTdenovo	99.89	1046
267X	MiniASM	99.90	1067
	Canu	99.91	981
	Flye	99.90	1049
	SMARTdenovo	99.89	998
Reference		99.90	785

Supplementary Table 7. BUSCO genome completeness assessment for each Nanopore *de novo* assembler at each genome coverage depth and polishing software.

Coverage	Assembler	Raw	Medaka	Racon	Pilon
10X	MiniASM	0.00	2.40	17.07	17.36
	Canu	0.00	11.28	91.00	93.69
	Flye	0.06	13.09	95.73	98.13
	SMARTdenovo	0.00	10.11	94.51	97.37
20X	MiniASM	1.81	25.37	71.30	71.65
	Canu	8.53	40.62	96.73	97.43
	Flye	19.23	43.54	98.01	98.36
	SMARTdenovo	23.44	33.49	97.43	98.25
40X	MiniASM	1.11	21.45	94.56	95.50
	Canu	2.75	22.79	97.84	98.54
	Flye	3.80	18.29	97.78	98.19
	SMARTdenovo	4.15	19.11	97.72	98.36
60X	MiniASM	0.29	6.66	27.94	28.17
	Canu	4.73	30.98	91.93	92.46
	Flye	15.37	38.92	98.07	98.19
	SMARTdenovo	24.90	39.98	97.78	98.31
Reference		98.25			

Supplementary Table 8. Percentage of *S. cerevisiae* S288C CDS found in each Nanopore *de novo* assembler at each genome coverage depth and polishing software.

Coverage	Assembler	Raw	Medaka	Racon	Pilon
10X	MiniASM	18.99	20.41	20.64	20.56
	Canu	6.42	95.07	95.67	95.73
	Flye	6.55	99.18	99.49	99.47
	SMARTdenovo	6.18	98.28	98.86	98.98
20X	MiniASM	11.02	75.23	75.60	75.43
	Canu	16.77	99.13	99.18	99.11
	Flye	20.44	99.40	99.39	99.39
	SMARTdenovo	99.59	99.69	99.76	99.69
40X	MiniASM	80.77	97.46	97.65	97.71
	Canu	98.43	99.27	99.40	99.40
	Flye	98.79	99.27	99.44	99.51
	SMARTdenovo	98.79	99.20	99.35	99.39
60X	MiniASM	22.95	31.26	31.39	31.38
	Canu	87.51	94.22	94.35	94.30
	Flye	98.60	99.13	99.16	99.18
	SMARTdenovo	98.89	99.40	99.45	99.47
Reference		99.66			

Supplementary Table 9. Evaluation of *de novo* Nanopore assemblers at various genome coverage depths with structure-related metrics.

Coverage	Assembler	N50 Avg (Mb)	# of Contigs Avg	Largest Contig Avg (Mb)
10X	MiniASM	0.067	48.0	0.122
	Canu	0.116	126.6	0.383
	Flye	0.220	99.4	0.752
	SMARTdenovo	0.074	56.0	0.141
20X	MiniASM	0.247	61.6	0.789
	Canu	0.667	38.2	1.064
	Flye	0.846	25.6	1.336
	SMARTdenovo	0.230	64.2	0.601
40X	MiniASM	0.810	19.2	1.407
	Canu	0.858	22.4	1.411
	Flye	0.890	21.8	1.518
	SMARTdenovo	0.682	29.8	1.069
60X	MiniASM	0.894	19.0	1.159
	Canu	0.908	22.0	1.496
	Flye	0.901	21.0	1.525
	SMARTdenovo	0.745	28.0	1.500
Reference		0.914	22.0	1.519

Table S10. All genetic parts used in this study

Name	Type	DNA sequence	Source	Ref.
Ptef1	Promoter	ATAGCTTCAAAATGTTTCTACTCCTTTTTTACTCTTCCAGATTTTCTCGGACACCGC<br>GCATCGCCGTACCACTTCAAAACACCCAAGCACAGCATACTAAATTTCCCCTCTTT<br>CTTCCTCTAGGGTGTCGTTAATTACCCGTACTAAAGGTTTGGAAAAGAAAAAAGTG<br>ACCGCCTCGTTTCTTTTTCTTCGTCGAAAAAGGCAATAAAAATTTTTATCACGTTTC<br>TTTTTCTTGAAAATTTTTTTTTTTGATTTTTTTCTCTTTCGATGACCTCCCATTGATAT<br>TTAAGTTAATAAACGGTGTTCAATTTCTCAAGTTTCAGTTTCATTTTTCTTGTTCTAT<br>TACAACTTTTTTTACTTCTTGCTCATTAGAAAGAAAGCATAGCAATCTAATCTAAGTT	Young, 2018	[1]
Psmtef1	Promoter	ATGCTTCAAAAACGCACTGTACTCCTTTTTACTCTTCCGGATTTTCTCGCACTCTCC<br>GCATCGCCGCACGAGCCAAGCCACACCCACACACCTCATACCATGTTTCCCCTCT<br>TTGTCTCTTTCGTGCGGCTCCATTACCCGCATGAAACTGTATAAAAGTAACAAAAG<br>ACTATTTCGTTTCTTTTTCTTTGTCGGAAAAGGCAAAAAAAAAAATTTTTATCACATT<br>TCTTTTTCTTGAAAATTTTTTTTGGGATTTTTTTCTCTTTCGATGACCTCCCATTGATA<br>TTTAAGTTAATAAAAGCACTCCCGTTTTCCAAGTTTTAATTTGTTCCTCTTGTTTAGT<br>CATTCTTCTTCTCAGCATTGGTCAATTAGAAAGAGAGCATAGCAAACTGATCTAAGT	Young, 2018	[1]
Phta1	Promoter	AAAGGGTGCAACGCGCGAAAAAGTGAGAACAGCCTTCCCTTTCGGGCGACATTGA<br>GCGTCTAACCATAGTTAACGACCCAACCGCGTTTTCTTCAAATTTGAACTCGCCGA<br>GCTCACAAATAATTCATTAGCGCTGTTCCAAAATTTTCGCCTCACTGTGCGAAGCTA<br>TTGGAATGGAGTGTATTTGGTGGCTCAAAAAAAGAGCACAATAGTTAACTCGTCGT<br>TGTTGAAGAAACGCCCGTAGAGATATGTGGTTTCTCATGCTGTTATTTGTTATTGCC<br>CACTTTGTTGATTTCAAAATCTTTTCTCACCCCCTTCCCCGTTCACGAAGCCAGCCA<br>GTGGATCGTAAATACTAGCAATAAGTCTTGACCTAAAAAATATATAAATAAGTCTCC<br>TAATCAGCTTGTAGATTTTCTGGTCTTGTTGAACCATCATCTATTTACTTCCAATCTG<br>TACTTCTCTTCTTGATACTACATCATCATACGGATTTGGTTATTTCTCAGTGAATAAA<br>CAACTTCAAAACAAACAAATTTCATACATATAAAATATAA	Young, 2018	[1]
Pagtef1	Promoter	AGCTTGCCTTGTCCCCGCCGGGTCACCCGGCCAGCGACATGGAGGCCCAGAATA<br>CCCTCCTTGACAGTCTTGACGTGCGCAGCTCAGGGGCATGATGTGACTGTCGCCC<br>GTACATTTAGCCCATACATCCCCATGTATAATCATTTGCATCCATACATTTTGATGG<br>CCGCACGGCGCGAAGCAAAAATTACGGCTCCTCGCTGCAGACCTGCGAGCAGGG<br>AAACGCTCCCCTCACAGACGCGTTGAATTGTCCCCACGCCGCGCCCCTGTAGAGA<br>AATATAAAAGGTTAGGATTTGCCACTGAGGTTCTTCTTTCATATACTTCCTTTTAAAA<br>TCTTGCTAGGATACAGTTCTCACATCACATCCGAACATAAACAAC	Young, 2018	[1]
Psptdh3	Promoter	GTTTTATTTCTGCTGCCATCCGTAAATGCCAGGATTTGAGCGGGTTACACAATATAT<br>CTCATATTTTCGGTGTCTGGGTCATTACTTTACTCTTGGCATCCACTAAATATATTG<br>GATCCTGCTTTTTAAACTGGCTTCCAGAAAAAAATCAATGGAGTGATGCAAACTGC<br>CTGGAGTAAAAGATGACACAAGGCGATTGACCTACGCATGTATCTATCTCATTTTC<br>TTACACCTTCTATTTCATTCTAACTCTTTGATTTGGAAAACACCTAAGAAAAAAAAG<br>GTTGAAATCAGTTCCCTGAAATTGTCCCCCTACTTGACTAATAAATATATAAAGACG<br>GTAGGTATTGACTGTAATTCGTAAATCTATACTTCTTAAACTTCTTCAAATTTACTTT<br>TTTGGATAGTCTTATTTTTGGTTTCAATACCCCAAGAACTTAGTTTCAAATAAATACA	Young, 2018	[1]
Ptdh3	Promoter	TTAGTCAAAAAATTAGCCTTTTAATTCTGCTGTAACCCGTACATGCCCAAAATAGGG<br>GGCGGGTTACACAGAATATATAACATCGTAGGTGTCTGGGTGAACAGTTTATTCCT<br>GGCATCCACTAAATATAATGGAGCCCGCTTTTTAAGCTGGCATCCAGAAAAAAAAA<br>GAATCCCAGCACCAAAATATTGTTTTCTTCACCAACCATCAGTTCATAGGTCCATTC<br>TCTTAGCGCAACTACAGAGAACAGGGGCACAAACAGGCAAAAAACGGGCACAACC<br>TCAATGGAGTGATGCAACCTGCCTGGAGTAAATGATGACACAAGGCAATTGACCCA<br>CGCATGTATCTATCTCATTTTCTTACACCTTCTATTACCTTCTGCTCTCTCTGATTTG<br>GAAAAAGCTGAAAAAAAAGGTTGAAACCAGTTCCCTGAAATTATTCCCCTACTTGA<br>CTAATAAGTATATAAAGACGGTAGGTATTGATTGTAATTCTGTAAATCTATTTCTTAA<br>ACTTCTTAAATTCTACTTTTATAGTTAGTCTTTTTTTTAGTTTTAAAACACCAAGAACT<br>TAGTTTCGAATAAACACACATAAACAAACAA	Young, 2018	[1]
Psbtdh3	Promoter	CATTCATCTTTCACCTGCCATTAGTAACCCGACTTCTCATTGAGCGGGTTACGGCA<br>GCCACAGGCCACATTCCGAATGTCTGGGTGAGCGGTCCCTTTTCCAGCATCCACT<br>AAATATCTCGGATCCCGCTTTTTAATCTGGCTTCCTGAAAAAAATCAATGGAGTGAT<br>GCAAACTGACTGGAGCAAAAAGCTGACACAAGGCAATCGACCTACGTGTCTGTCT<br>ATTTTCTCACACCTTCTATTACCTTCTAACTCTCTGGGTTGGAAAAAACTGAAAAAA<br>AGGTTGTCTCCAGTTTCCACAAATCATCCCCCTGTTTGATTAATAAATATATAAAGA<br>CGACAACTATCGATCATAAACTCATAAAACTATAACTCCTTTACACTTCTTATTTTAT<br>AGTTATTCTATTTTAATTCTTATTGATTTTAAAACCCCAAGAACTTAGTTTCGAAAAC<br>ACACACACACAAACAATTAAA	Young, 2018	[1]
Phta2	Promoter	AAAGGGTGCAACGCGCGAAAAAGTGAGAACAGCCTTCCCTTTCGGGCGACATTGA<br>GCGTCTAACCATAGTTAACGACCCAACCGCGTTTTCTTCAAATTTGAACTCGCCGA<br>GCTCACAAATAATTCATTAGCGCTGTTCCAAAATTTTCGCCTCACTGTGCGAAGCTA<br>TTGGAATGGAGTGTATTTGGTGGCTCAAAAAAAGAGCACAATAGTTAACTCGTCGT<br>TGTTGAAGAAACGCCCGTAGAGATATGTGGTTTCTCATGCTGTTATTTGTTATTGCC<br>CACTTTGTTGATTTCAAAATCTTTTCTCACCCCCTTCCCCGTTCACGAAGCCAGCCA<br>GTGGATCGTAAATACTAGCAATAAGTCTTGACCTAAAAAATATATAAATAAGTCTCC<br>TAATCAGCTTGTAGATTTTCTGGTCTTGTTGAACCATCATCTATTTACTTCCAATCTG<br>TACTTCTCTTCTTGATACTACATCATCATACGGATTTGGTTATTTCTCAGTGAATAAA<br>CAACTTCAAAACAAACAAATTTCATACATATAAAATATAA	Young, 2018	[1]
Psnr52	Promoter	TCTTTGAAAAGATAATGTATGATTATGCTTTCACTCATATTTATACAGAAACTTGATG<br>TTTTCTTTCGAGTATATACAAGGTGATTACATGTACGTTTGAAGTACAACTCTAGAT<br>TTTGTAGTGCCCTCTTGGGCTAGCGGTAAAGGTGCGCATTTTTTCACACCCTACAA<br>TGTTCTGTTCAAAAGATTTTGGTCAAACGCTGTAGAAGTGAAAGTTGGTGCGCATG<br>TTTCGGCGTTCGAAACTTCTCCGCAGTGAAAGATAAATGATC	Young, 2018	[1]

Pact1	Promoter	AAAATGTGTGGGGAAGCGGGTAAGCTGCCACAGCAATTAATGCACAACATTTAACC<br>TACATTCTTCCTTATCGGATCCTCAAAACCCTTAAAAACATATGCCTCACCCTAACA<br>TATTTTCCAATTAACCCTCAATATTTCTCTGTCACCCGGCCTCTATTTTCCATTTTCT<br>TCTTTACCCGCCACGCGTTTTTTTCTTTCAAATTTTTTTCTTCTTTCTTCTTTTTCTTC<br>CACGTCCTCTTGCATAAATAAATAAACCGTTTTGAAACCAAACTCGCCTCTCTCTCT<br>CCTTTTTGAAATATTTTTGGGTTTGTTTGATCCTTTCCTTCCCAATCTCTCTTGTTTA<br>ATATATATTCATTTATATCACGCTCTCTTTTTATCTTCCTTTTTTTCCTCTCTCTTGTA<br>TTCTTCCTTCCCCTTTCTACTCAAACCAAGAAGAAAAAGAAAAGGTCAATCTTTGTT<br>AAAGAATAGGATCTTCTACTACATCAGCTTTTAGATTTTTCACGCTTACTGCTTTTTT<br>CTTCCCAAGATCGAAAATTTACTGAATTAAC	Young, 2018	[1]
Pgal10	Promoter	TGCGGCGCGAGGCACATCTGCGTTTCAGGAACGCGACCGGTGATGACGAGGACG<br>CACGGAGGAGAGTCATCCGTCGGAGGGCTGTCGCCCGCTCGGCGGCTTCTAATC<br>CGTACTTCAATATAGCAATGAGCAGTTAAGCGTATTACTGAAAGTTCCAAAGAGAA<br>GGTTTTTTTAGGCTAAGATAATGGGGCTCTTTACATTTCCACAACATATAAGTAAGA<br>TTAGATATGGATATGTATATGGTGGTAATGCCATGTAATATGATTATTAAACTTCTTT<br>GCGTCCATCCAAAAAAAAAAGTAAGAATTTTTGAAAATTCAATATAA	Young, 2018	[1]
Psptef1	Promoter	ATAGCCGACAAATCTTTTACTCCTTTTTTTACTCTTCCGCATTTTCTCGGACTGCGC<br>GCATCGCCGCACCGCTTCCAAAACACCTGAACATTACATACTATTTTTCCCCTCTTT<br>CTTTCTTTAGGGTGGTGTTAAATTACCCGCTCTGAAGCTTTGGAAAAGAAACAAAA<br>GGCCACTTCGTTTCTTTTTCTTCGTCGAAAAGGGCAAAAAAAAAATTTTTACCACGT<br>TTCTTTTTCTTGAAAATTTTTTTTTTTTTATTTTTTCTCTTTCGATGACCTCCCATTGAT<br>ATTTAAGTTAATAAATGGTCATCAATTTCTCAAGTTTTATTTCGTTTTCCTTGTTTCA<br>TGTCGACTTTTTTACATCCTTCTCAGTTAGAAAGAAAGCATAGCAATCTAATCTAAG	Young, 2018	[1]
Ppgk1	Promoter	TCCCTCCTTCTTGAATTGATGTTACCCTCATAAAGCACGTGGCCTCTTATCGAGAAA<br>GAAATTACCGTCGCTCGTGATTTGTTTGCAAAAAGAACAAAACTGAAAAAACCCAG<br>ACACGCTCGACTTCCTGTGTTCCTATTGATTGCAGCTTCCAATTTCGTCACACAACA<br>AGGTCCTAGCGACGGCTCACAGGTTTTGTAACAAGCAATCGAAGGTTCTGGAATG<br>GCGGGAAAGGGTTTAGTACCACATGCTATGATGCCCACTGTGATCTCCAGAGCAA<br>AGTTCGTTCGATCGTACTGTTACTCTCTCTCTTTCAAACAGAATTGTCCGAATCGTG<br>TGACAACAACAGCCTGTTCTCACACACTCTTTTCTTCTAACCAAGGGGGTGGTTTA<br>GTTTAGTAGAACCTCGTGAAACTTACATTTACATATATATAAACTTGCATAAATTGGT<br>CAATGCAAGAAATACATATTTGGTCTTTTCTAATTCGTAGTTTTTCAAGTTCTTAGAT<br>GCTTTCTTTTTCTCTTTTTTACAGATCATCAAGGAAGTAATTATCTACTTTTTACAAC	Young, 2018	[1]
Psktef1	Promoter	GCAGAAATGGGAAAACATTGGCGTTGGCAATGGCTGCTAAGTGACTGCCACATGT<br>CAAGGTGTTAGCATGCAGATGTACCCATTGGACACCGATGTTGCAGTAGTAGGACT<br>GTTTCAAGCAATACATGCCTAGCGTGGACGCAAACACACGTACACCAAGCAAAAAC<br>CATCTTTTGCATGACAGAGATCTGATAAACCCAGTCACCGCATCAACAGTCAGCCC<br>AAAACGGGCCATTAAAAATGACTCTGACACCACCGGCCCCGCACGCAGAGAGCGC<br>TTCGAACGGCCACGTTGCCCTAACCTTTTGGCTCGGCGAAGAGGGACCAACTTTC<br>CATAAACGGCAAGCATCAGAAACCGTATCCGGAAATACCTGCCATATACGGAAGGT<br>AATGAAACACGACACCCATTCCCCATACTGTCGTTTCTTTTTTATCCCGAGGCGCT<br>CATTACTCAACGTAATAGCCCACACGGTGGCAAACTCTCACGCAGGAAAAATTTTT<br>TTCTGGTATAAAAGCATTGGGATGGCCCCCTGTTCCCTCTCCCTTCTGCTTTTTTCC<br>TTTTGGTTCTTTTCTTAAACCAACGAATACTAATAGTAAAA	Young, 2018	[1]
Psmtdh3	Promoter	GCGGCTAAAAAGCTAAATATTCCAAAAAAACATAAAACAAAAGTTTGAGCCAATTAA<br>CCCAAATAATCTGTATCAGGGTAACGATACCTCGAGCTTCCACTAAATTCTAGTAC<br>GTCACGTTGTACGAAGTATAGAGCATCCTGAAATAATGAAAAAAAGAAACAATTGG<br>CCGACTCCGCTTGTTTCTATTCCTTTCCACGTGATCTGAATTATTTACAGGAAGTCG<br>CATTTTTTGCGCAGTTGTTGCTGGGTAGCAGCGGCCAGCAAGGTCTGGTGAAACT<br>CGACTGATGCGTTGAGGACTAGAAACTGGTGGTGACAGCTAAACTGAGCTCCTCA<br>ACCTGATCATGTCATGGCTGTCTCAAAGATCTTGCAGACTGTAACTACGAGCGCAT<br>ACGTATGCGCATACGTGTCTGATAGTACCCAGTGATCTGATCTATATTTTTTTGGG<br>GGGGTTTTGAAAGGATATATAAAGGTTACACTTCCTCAGTTTAGTTTATTCTTGAGT<br>TTGAAGTTCCTTATGATTATACTTGTCTTAAATATTAAAAAAAACCAAGAACTTAGTT<br>TCAAATTAAATTCATCACATACACAAACAAAACAAA	Young, 2018	[1]
Psbtef1	Promoter	CTTCATTGCTGTCTCATTTCCCTCTGATGGACGCACACGCTGGTGCATCCACCGCA<br>GCAGCGCCAGCGTATACACATTCTTGGATTCCACGCCACCCACCCTCATTTATAGG<br>CCGCCAGACGTACTTTCCCGCGACTGCGCCCTTCTATGATACCACACCCGTGCCC<br>CCACTGAACACCCACACATTTCTGACACCCACACACACGGTTAACCGCCCACACTG<br>TCTCGAAGTCGGTCGTACAAGGGGACGTGGGCGCCTTCTGAGCCCACCAGAAAAA<br>GGAAGGGGAATGCAAAGTTTCCGGCAGAAACCTCAAGAAAAAGTTTTTCCTCTTCG<br>AATATGCTGTATGCGGGAATGATCGAGCCTGTAGTTAATGATATATAAATTAGTTAT<br>TTCCATCGCCTTCTTTTCTCGTGTCGCTCGTTCTAGTGCTATTTCTGGCTTTTTCCC<br>TCTTTTTTTTCATCTTCCTTTTATTTTCTTTCTAATATATAAATTCTCTTGCATTTTTCT<br>TTTTTTCTTGTACTTATTTATTCTCCTTCTAGGTTTTATTTTTTGATCGTTTCTCAGAA<br>TAAAAAGTCAACGAAACTATAACTAACTAAAC	Young, 2018	[1]
Psktdh3	Promoter	ATAGCCAGCCATCAGACACTGGTTACCGTATTCTTCTTTACCTTTGTTTGTGGCGG<br>GACGCTGTTGTCTCTGTGTGTATGGATGTAACTATATACGAATGGTGGCTCGCCAG<br>GCGTAGCCGCCATTTCCGAGAGGTTTCTTGTCCCACGCTGGCGCGTCCTCTCACC<br>AACGGTTTCCCTTGTTTAGTCCGGAGTTTTACTGGGTGTCCCTAATGGAAACAGTG<br>GAAGCCCCGTCACCAGATGCACATACCTTGGTGGAAGTGTTCGGCTGTGGAGTGA<br>CATCGAATGAACGTGGTCGCCAGAGGCCTATAACTGGGTGCTACACGTTGCAAAA<br>GAAAAGTGTAGTAATTGGTATATAAGGAACTTGGATATGCTACATTGCCAGTTAGAA<br>GGTTCGATCTTTTTTTCTCTCTCTTACTCCTAAACCATCAACGAACTTAACGGTCCG<br>CAGATAATTTCCCCATATTAACTCGCACAACACTCTAGATAAACAAACTAACAAA	Young, 2018	[1]

Prev1	Promoter	AACGGTGTTGTTATCCGATACAACCGGATATTTTTCTTTTAATGAGTCTAAACCGTG<br>ATAGCTTCAGGTTAATACAATCAAAAAAAGCTCAAATATTCTTTTAATGCCGCGTTC<br>ACAGATTCCAATTGAATACAACTAGGTAGTTCATTATATGAAGCCTTTGCTACTATT<br>TTTCACTATAGTCTGCCTTCACCTTAATGCAGACATCCACATATTTTAATCACTTTAA<br>AATAAAAAGGAAGATATATTAGAAGCTATGATCCAATCTGTAAGCCAGATTAAAATT<br>CACGAACTCTTCTTTCATTTGAATTGAATGCTTTGAGTTGGGGTAGATTATCGCAAA<br>TTACTCATCACATTTATTGACTACGAACTTGCTGATGTCCTTTTTTTATTTATATTTTT<br>CTTCAGTGAAGCGATTTTTTTTTTACACAGACCAAGACGGAAAAAAGTAGCTAAGG<br>AAGAAAACAAAATCATGAAAAAAATGTGAAGTGATCATGCACATCGCATCAACTTAA<br>ACATTGGCTTAGAGATATATAGAGTTAGAGTTTACGGCAACCTTTAAGCACCAATAC<br>CTTTTGGCATAGTCTAAAGACCTGGTTCTTAATTTTAAACAAATTTAACTAAAGATTT<br>CCCTATCAAAGAAGTAACGAGTTGACAGATTTTCTCAAAATAAATCGATACTGCATT<br>TCTAGGCATATCCAGCGAGATC	Lee, 2015	[2]
Ptrp1	Promoter	AACGACATTACTATATATATAATATAGGAAGCATTTAATAGACAGCATCGTAATATAT<br>GTGTACTTTGCAGTTATGACGCCAGATGGCAGTAGTGGAAGATATTCTTTATTGAA<br>AAATAGCTTGTCACCTTACGTACAATCTTGATCCGGAGCTTTTCTTTTTTTGCCGAT<br>TAAGAATTAATTCGGTCGAAAAAAGAAAAGGAGAGGGCCAAGAGGGAGGGCATTG<br>GTGACTATTGAGCACGTGAGTATACGTGATTAAGCACACAAAGGCAGCTTGGAGT	Verwaal et al., Addgene, Plasmid #101748	[3]
Pgal1	Promoter	CTAGTACGGATTAGAAGCCGCCGAGCGGGTGACAGCCCTCCGAAGGAAGACTCTC<br>CTCCGTGCGTCCTCGTCTTCACCGGTCGCGTTCCTGAAACGCAGATGTGCCTCGC<br>GCCGCACTGCTCCGAACAATAAAGATTCTACAATACTAGCTTTTATGGTTATGAAGA<br>GGAAAAATTGGCAGTAACCTGGCCCCACAAACCTTCAAATGAACGAATCAAATTAA<br>CAACCATAGGATGATAATGCGATTAGTTTTTTAGCCTTATTTCTGGGGTAATTAATC<br>AGCGAAGCGATGATTTTTGATCTATTAACAGATATATAAATGCAAAAACTGCATAAC<br>CACTTTAACTAATACTTTCAACATTTTCGGTTTGTATTACTTCTTATTCAAATGTAAT<br>AAAAGTATCAACAAAAAATTGTTAATATACCTCTATACTTTAACGTCAAGGAGAAAA<br>AACCCCGGATTCTAGA	Hegemann et al., Euroscarf, P30672	[4]
UAS18B-TEF(136)	Promoter	CTGAGGTGTCTCACAAGTGCCGTGCAGTCCCGCCCCCACTTGCTTCTCTTTGTGT<br>GTAGTGTACGTACATTATCGAGACCGTTGTTCCCGCCCACCTCGATCCGGTCTAGA<br>CTGAGGTGTCTCACAAGTGCCGTGCAGTCCCGCCCCCACTTGCTTCTCTTTGTGT<br>GTAGTGTACGTACATTATCGAGACCGTTGTTCCCGCCCACCTCGATCCGGGGATC<br>CCTGAGGTGTCTCACAAGTGCCGTGCAGTCCCGCCCCCACTTGCTTCTCTTTGTGT<br>GTAGTGTACGTACATTATCGAGACCGTTGTTCCCGCCCACCTCGATCCGGGTCGA<br>CCTGAGGTGTCTCACAAGTGCCGTGCAGTCCCGCCCCCACTTGCTTCTCTTTGTGT<br>GTAGTGTACGTACATTATCGAGACCGTTGTTCCCGCCCACCTCGATCCGGGAGCT<br>CCTGAGGTGTCTCACAAGTGCCGTGCAGTCCCGCCCCCACTTGCTTCTCTTTGTGT<br>GTAGTGTACGTACATTATCGAGACCGTTGTTCCCGCCCACCTCGATCCGGTCTAGA<br>CTGAGGTGTCTCACAAGTGCCGTGCAGTCCCGCCCCCACTTGCTTCTCTTTGTGT<br>GTAGTGTACGTACATTATCGAGACCGTTGTTCCCGCCCACCTCGATCCGGGGATC<br>CCTGAGGTGTCTCACAAGTGCCGTGCAGTCCCGCCCCCACTTGCTTCTCTTTGTGT<br>GTAGTGTACGTACATTATCGAGACCGTTGTTCCCGCCCACCTCGATCCGGGTCGA<br>CCTGAGGTGTCTCACAAGTGCCGTGCAGTCCCGCCCCCACTTGCTTCTCTTTGTGT<br>GTAGTGTACGTACATTATCGAGACCGTTGTTCCCGCCCACCTCGATCCGGGCATG<br>CCTGCAGAAGCTTTTGTGGTTGGGACTTTAGCCAAGGGTATAAAAGACCACCGTCC<br>CCGAATTACCTTTCCTCTTCTTTTCTCTCTCTCCTTGTCAACTCACACCCGAAATCG<br>TTAAGCATTTCCTTCTGAGTATAAGAATCATTCAAAGGCGCGCCACA	Schwartz et al., Addgene, Plasmid # 70007	[5]
Pgap	Promoter	TTTTTGTAGAAATGTCTTGGTGTCCTCGTCCAATCAGGTAGCCATCTCTGAAATATC<br>TGGCTCCGTTGCAACTCCGAACGACCTGCTGGCAACGTAAAATTCTCCGGGGTAA<br>AACTTAAATGTGGAGTAATGGAACCAGAAACGTGTCTTCCCTTCTCTCTCCTTCCA<br>CCGCCCGTTACCGTCCCTAGGAAATTTTACTCTGCTGGAGAGCTTCTTCTACGGCC<br>CCCTTGCAGCAATGCTCTTCCCAGCATTACGTTGCGGGTAAAACGGAGGTCGTGT<br>ACCCGACCTAGCAGCCCAGGGATGGAAAAGTCCCGGCCGTCGCTGGCAATAATAG<br>CGGGCGGACGCATGTCATGAGATTATTGGAAACCACCAGAATCGAATATAAAAGG<br>CGAACACCTTTCCCAATTTTGGTTTCTCCTGACCCAAAGACTTTAAATTTAATTTATT<br>TGTCCCTATTTCAATCAATTGAACAACTAT	Obst et al.	[6]

ahas1	Gene	ATGCAATCAACGGCGACTGCGAGCAACCCGGAGAGTCGTCCCGTTCCATCTCCTG<br>CTTTCAACCAAGAGCCGCATAGGAATGAGATCAGCCCCCTACAGCATAGACAGCTT<br>CCTGAACTTGATGACTCTATGGTTGGTATGAGTGGTGGCGAGATCTTCCACGAGAT<br>GATGCTAAGGCAAGGGGTCAAACACGTCTTTGGGTACCCCGGAGGAGCCATTCTA<br>CCCGTGTTTGATGCGATTTACAATTCCAAACACTTCGAGTTCATATTGCCAAAACAC<br>GAGCAGGGCGCGGGTCATATGGCAGAGGGTTACGCTAGGGCCTCAGGAAAACCG<br>GGTGTGGTCCTAGTGACGAGCGGGCCTGGCGCGACTAACGTTATTACCCCAATGC<br>AGGATGCTATGAGCGACGGGACTCCGATGGTAGTCTTTTGCGGACAAGTTCCTAC<br>CTCCGCGATCGGGACTGACTCATTTCAGGAGGCCGACGTGATAGGAATAAGCCGT<br>GCGTGCACAAAGTGGAATGTGATGGTGAAATCTGTGGGAGAACTGCCACGTCGTA<br>TTCAAGAGGCCTTTGAAATTGCAACTTCTGGGCGTCCCGGTCCTGTATTAGTAGAT<br>CTGCCTAAGGATGTGACGGCGGGTATCTTAAGGAATCCCATACCCATGCACAGCA<br>CTATACCATCCTTGCCGAGTGCCGCTACCGTGGCCGCGCGTGAAATGAGCCGTAA<br>ACAACTGGAGGGCACTATTAATCGTGTTGCTAATTTGGTGAACGTAGCTAAAAAAC<br>CTATTCTGTATGTCGGGCAAGGTTTATTAGCCCGTCCCGACGGGCCTGAGATCTTA<br>AAGGAGTTTGCGGATAAGGCATGCATACCAGTGACTACGACTTTGCAAGGCTTAG<br>GGGGCTTTGATGAGCTGGACCCAAAAGCCCTACATATGTTGGGCATGCATGGTTC<br>AGCTTACGCCAACATGGCGATGCAAGAAGCGGATCTAATTATTGCGGTAGGAGCT<br>CGTTTCGATGACAGAGTCACGTTGTCTATCCCGAAATTCGCGCCGCAAGCAAAGTT<br>AGCCGCTACTGAGGGCCGTGGTGGGATTGTCCATTTCGAGATAATGCCTAAAAAC<br>ATTAACAAAGTTGTGCAGGCAAACGAGGCGGTCGAGGGTGACTGTGCTGACAATC<br>TACGTCTTTTATTGCCTCACGTAAAGGCTGTCTCTGAAAGGCCTGAATGGTTTGAA<br>CAAATTAATGACTGGAAACAAAGGTTCCCTCTATCTTTATACGACCGTCAGACTGA<br>GGATGGGCCCATTAAGCCACAAGCCGTAATCGAAAAGCTATCAGAATTGACGGCT<br>GATCGTAAAGAGAAAACTATAATAACTACGGGTGTCGGCCAGCATCAAATGTGGAC<br>GGCGCAACACTTCAGGTGGAGGCATCCCCGTACGATGATAACGAGTGGAGGACTG<br>GGCACGATGGGGTATGGTTTACCTGCAGCATTAGGCGCTAAGGTTGCTAGACCCG<br>ACTGTCTTGTCATCGACATCGACGGTGACGCCAGTTTCAATATGACACTGACAGAA<br>TTGAGCACAGCAGCGCAATTCAATATCGGCGTTAAGGTCCTGTTGATCAATAATGA<br>AGAACAGGGTATGGTGACACAGTGGCAGAACCTGTTCTACGAGGACCGTTACTCA<br>CATACGCATCAGCAGAACCCGGATTTCGTTCCGCTTGCCAAGGCAATGAGAATCG<br>GGGCTGATACGTGTTTCAAACCTAGCGAGTTGGAAGAGAAGTTAAAGTGGTTAATT<br>GAACATGATGGTCCCGCACTTCTGGAGGTTATTACAGATAGGAAAGTGCCCGTATT<br>GCCCATGGTCCCAAGCGGGAGAGGCCTACACGAATTTCTAGTTTACGACGAAGCG<br>AAGGACTTAGAGCGTAAAGAACTTATGCGTGAGAGGAACGTAGACTTCAGTGTCA	This study	van den Berg, 2008<br>[7]
ilv6	Gene	ATGGGGATAAAGACTATCGACTTCGCAGGTGTCAAGGAGGACGTTTACGAGCGTG<br>CTGATTGGCCAAGGGAGAAACTACAAGAATATTTCAAAAATGACACACTAGCCCTA<br>ATCGGTTATGGTTCCCAGGGACACGGACAGGGCCTTAATCTAAGGGACCAGGGAC<br>TTAATGTAATAGTCGGGGTAAGGAAGGACGGGGCGAGCTGGAAAGAAGCGATCCA<br>GGATGGATGGGTGCCTGGCAAGAATCTATTCGATGTAACGACGGCTATCGAAAAG<br>GGTACCGTAATAATGAATCTACTTTCCGACGCAGCACAATCCGAGACGTGGCCGA<br>CAATAAAACCGCTTCTAACTAAAGGTAAAACGCTGTACTTCTCACACGGCTTTTCTC<br>CGGTTTTTAAAGACTTGACTAAAGTTGACGTGCCGAAAGATATTGATGTAATATTAG<br>TTGCTCCTAAGGGGTCAGGCAGGACGGTAAGAACACTGTTCCGTGAGGGGCGTG<br>GTATAAACAGTTCAATCGCAGTCTTTCAGGACGTAACAGGCCAAGCAAAAGAGAGG<br>GCAATAGCTATGGGCGTTGCAGTTGGCAGCGGTTATTTATATGAAACAACGTTCGA<br>AAAGGAGGTCTACAGCGACCTGTATGGAGAGAGAGGCTGCCTGATGGGAGGTATC<br>CATGGTATGTTCCTGGCGCAATATGAGGTCTTACGTGAGAGAGGGCACTCTCCGT<br>CTGAAGCATTTAACGAGACTGTCGAAGAAGCCACTCAGAGCTTATACCCCCTAATC<br>GGGGGCAATGGTATGGACTGGATGTACGCCGCGTGCTCTACGACCGCAAGAAGG<br>GGGGCAATCGACTGGTCCTCAAGGTTCAAAGATACATTGAAGCCTGTGTTTAATGA<br>CTTGTACGACTCAGTACACAACGGCACTGAGACAAAAAGGTCACTAGACTATAACT<br>CTCAACCGGACTATAGAGAAAAGTATGAGAAAGAGATGCAAGAAATCAGAGATTTG<br>GAGATTTGGAGGGCTGGTAAAGCTGTTCGTTCTCTAAGACCAGAGAATCAGAAGTA	This study	van den Berg, 2008<br>[7]

ilvD1	Gene	ATGGAACAGAATCCGAAAAAAGATTACGACCTGACGAATCCGATCACGTCAACATC<br>AGGATTGAGGCAAGGATTAACAAGCTACGGCGACGCCCACTTCAGCTTGTTCCTG<br>AGGAAGGTCTTTATTAAGGCGCTGGGGTACTCAGAGGACGCACTTTCAAGGCCTA<br>TAGTCGGAATCATAAATACTTTTTCTGGATTTAATCCTTGCCATGCGAACGTGCCGC<br>AACTGATAGAAGCAGCAAAGCGTGGTGTGCAACTGAACGGTGGCCTAGCAATAGA<br>GTTTCCTACGATCAGTCTAGCGGAGTCATTCAGCCATCCCACGTCAATGTTTCTGA<br>GAAATCTAATGTCAATGGACACGGAAGAAATGATCAGAGCCCAACCACTAGACGCT<br>TGCATCATGATAGGCGGCTGTGATAAGACCGTTCCAGCGCAACTTATGGGAGGAA<br>TAAGCGCGAATAAGCCAATACTGCCTCTAATTACCGGACCTATGTTGCCTGGTTCA<br>CACCGTGGGCAACGTATCGGTGCATGTACTGACTGTAGGAACAACTGGGCCGCAT<br>TCAGAGCCGGGGAGATTGACGTTGAGGAAATCTCAGCCATCAACGAAGAGTTAGC<br>CCCGACGATAGGCACGTGTGGCGTGATGGGGACGGCCTCAACTATGGCGTGCGT<br>AACCGCAGCGCTAGGCATGATGCCCCTGAGGGGCGCGACAGCTCCCGCTGTGTC<br>CTCTGCACGTTTGCGTATTGCCGAAGAAACGGGAGCTAGCGCTGTAGCAATTGCT<br>AAGTCAAAACGTAAGCCTCAAGAAATTTTGACAAAGGAGTCTTTTTGGAACGCCAT<br>CACGGTCCTACAGGCAATCGGTGGTAGTACCAACGCTGTGGTGCATTTACTTGCTA<br>TAGCAAATAGACACCCAGAACTGCAAGGGGTTATCACCCTAGATACAGTCGAGGA<br>AATAGGGCGTCGTACTCCGCTACTTATCGACCTGAAGCCGAGTGGTGATAATTATA<br>TGAACGATTTTCACAATGCAGGTGGCATGATGGCTCTACTACAGGTGTTAAGGCCT<br>CTTCTACATTTATCAGCCTTAACAGTTACAGGTCAGACACTGGGGGAGGTTTTGGA<br>TGCTTCCCAAAGCAAAAGGCTTGTATTCGCACAGCAAATAATACGTCCTATTTCTGA<br>TCCACTGTTTCCAGCTAGTTCCCTAGCCGTCCTTCGTGGAAACTTAGCTCCGGATG<br>GAGCAGTACTTAAGGCAAGCGCATCAAAGTACAGGCACCTGCTAACGCACACTGG<br>GCCCGCAGTTGTATTTGAGAATTCAGCCGATTTAGCTCATCGTATCGACGACCCCA<br>ATTTGTTGGTTACCAAGGACTCCGTTCTGGTGTTAAAGAACATTGGACCTGTTGGA<br>AACCCCGGCATGCCGGAAGCTGGGCTTATACCGATACCTAAGAAACTAGCTGAGG<br>AAGGCGTAAAGGACATGATAAGGTTAAGTGATGGTAGAATGAGCGGAACTGCGGG<br>CGGCACTATCATACTTCACATTTCACCTGAGGCCGCACTACCGGAGAGCCCCTTC<br>GGGGTTGTGGAGACGGGGGATCTTATAGTCTGTGACATCGAGACACGTAAGCTTC<br>ATCTGGAAGTTACCGAGGCTGTGCTTCAAACGAGAATAGAAAAACGTAGACAGAG<br>CCTTGTAGAAGAAACTAGAGCCCGTAAGCAGATGCGTGGCTATCGTGGCCTTTAC<br>GAGAGGTCTGTAAACCAAGCGCAGGAGGGGGCGGATTTTGATTTCCTGACGGCTG	This study	van den Berg, 2008<br><br>[7]
nat	Gene	ATGGGTACCACTCTTGACGACACGGCTTACCGGTACCGCACCAGTGTCCCGGGGG<br>ACGCCGAGGCCATCGAGGCACTGGATGGGTCCTTCACCACCGACACCGTATTCCG<br>CGTCACCGCCACCGGGGACGGCTTCACCCTGCGGGAGGTGCCGGTGGACCCGC<br>CCCTGACCAAGGTGTTCCCCGACGACGAATCGGACGACGAATCGGACGACGGGG<br>AGGACGGCGACCCGGATTCCCGGACGTTCGTCGCGTACGGGGACGACGGCGACC<br>TGGCGGGCTTCGTGGTCGTCTCGTACTCCGGCTGGAACCGCCGGCTGACCGTCG<br>AGGACATCGAGGTCGCCCCGGAGCACCGGGGGCACGGGGTCGGGCGCGCGTTG<br>ATGGGGCTCGCGACGGAGTTCGCCCGCGAGCGGGGCGCCGGGCACCTCTGGCT<br>GGAGGTCACCAACGTCAACGCACCGGCGATCCACGCGTACCGGCGGATGGGGTT<br>CACCCTCTGCGGCCTGGACACCGCCCTGTACGACGGCACCGCCTCGGACGGCGA<br>GCAGGCGCTCTACATGAGCATGCCCTGCCCCTAA	Young, 2018	[1]
ado	Gene	ATGTACACAGTAGGTGACTATCTGTTGGATAGACTGCATGAATTAGGGATTGAGGA<br>AATATTCGGGGTCCCGGGTGACTATAACTTACAATTTCTGGATCAAATTATTAGTAG<br>AAAAGACATGAAGTGGGTCGGGAATGCGAATGAGTTGAATGCTAGTTATATGGCTG<br>ATGGCTATGCAAGGACAAAGAAGGCTGCTGCGTTTCTGACAACATTTGGAGTTGG<br>CGAGCTAAGCGCGGTAAATGGGTTGGCTGGGAGCTATGCGGAGAATCTTCCAGTA<br>GTGGAGATAGTCGGATCACCCACCTCCAAAGTCCAGAATGAGGGCAAGTTCGTAC<br>ATCACACCTTAGCCGATGGAGATTTTAAGCACTTCATGAAGATGCATGAGCCCGTT<br>ACTGCTGCCAGAACGCTACTAACGGCAGAAAATGCCACGGTAGAAATTGACCGTG<br>TATTGTCTGCTCTATTAAAGGAGAGGAAGCCCGTATACATTAATCTTCCGGTAGAC<br>GTCGCCGCCGCTAAGGCTGAGAAGCCTAGCTTACCTCTAAAGAAAGAGAACCCGA<br>CTAGTAATACATCAGATCAGGAAATACTAAATAAGATTCAGGAGTCATTAAAAAACG<br>CGAAAAAGCCGATAGTCATCACAGGACATGAAATAATTTCCTTCGGGTTGGAGAAC<br>ACAGTAACTCAATTCATCAGTAAGACGAAGCTACCAATAACTACGCTTAATTTTGGG<br>AAGTCATCAGTAGACGAAACATTGCCGTCTTTTCTGGGGATCTATAACGGAAAGTT<br>GTCCGAGCCGAATTTGAAGGAGTTCGTGGAATCAGCGGATTTCATTCTGATGTTGG<br>GGGTAAAATTGACGGATAGTTCCACCGGCGCATTCACTCACCACTTAAACGAGAAC<br>AAGATGATTAGTTTAAACATCGATGAAGGGAAAATATTCAACGAGTCAATCCAGAAT<br>TTCGACTTCGAGTCTTTGATAAGCTCTCTACTGGATCTGTCAGGTATTGAGTACAA<br>GGGTAAATACATAGATAAGAAACAAGAGGATTTTGTCCCGAGTAATGCGTTGCTGA<br>GTCAGGACCGTCTTTGGCAGGCAGTAGAAAATCTTACGCAATCCAATGAGACGATC<br>GTTGCTGAACAAGGGACGAGTTTCTTTGGGGCAAGTTCAATCTTTTTGAAACCTAA<br>AAGTCATTTCATTGGGCAGCCGCTGTGGGGAAGCATCGGTTACACTTTCCCCGCC<br>GCGCTGGGGAGCCAAATCGCAGATAAGGAAAGCCGTCACTTACTTTTTATTGGGG<br>ACGGTTCTTTACAATTGACAGTTCAAGAACTAGGTTTAGCGATAAGGGAAAAAATAA<br>ACCCAATCTGCTTCATTATCAACAACGATGGCTATACGGTCGAAAGGGAGATACAC<br>GGGCCGAACCAATCCTATAACGACATCCCGATGTGGAACTATAGTAAACTTCCCGA<br>ATCCTTTGGTGCTACCGAGGAGCGTGTAGTATCTAAGATCGTGCGTACCGAGAAT<br>GAATTTGTCAGCGTCATGAAAGAAGCGCAGGCGGATCCAAATAGGATGTATTGGAT<br>AGAGTTGGTCCTTGCAAAAGAGGACGCACCCAAGGTTCTTAAAAAAATGGGAAAAC<br>TTTTCGCTGAGCAAAACAAATCTTAA	This study	

kivD	Gene	ATGCCCACGCTGGAGATGCCGGTAGCTGCCGTATTAGATTCAACCGTGGGCTCCT<br>CTGAGGCTCTGCCTGATTTTACCTCAGATAGATACAAAGATGCATACTCCAGAATC<br>AATGCCATAGTGATAGAGGGCGAACAGGAGGCACATGACAACTATATTGCCATCG<br>GTACACTTCTTCCTGATCACGTAGAAGAATTAAAAAGGCTGGCAAAAATGGAGATG<br>AGACATAAGAAGGGTTTCACGGCTTGCGGAAAGAATTTAGGCGTTGAAGCGGATA<br>TGGATTTTGCTAGAGAGTTTTTCGCCCCACTACGTGACAATTTTCAGACCGCATTA<br>GGCCAGGGGAAAACTCCGACTTGTTTATTGATACAAGCCCTATTGATCGAGGCGTT<br>CGCAATTTCCGCTTATCACACCTACATCCCCGTATCCGATCCGTTCGCAAGGAAGA<br>TCACAGAAGGGGTCGTGAAGGATGAGTACACACACCTAAACTACGGCGAGGCTTG<br>GCTAAAGGCAAACCTGGAAAGCTGCAGGGAAGAACTTTTGGAGGCTAACCGTGAG<br>AATCTGCCCCTAATTCGTCGTATGTTAGATCAGGTGGCAGGGGACGCAGCTGTGC<br>TACAGATGGACAAAGAAGATCTTATAGAAGATTTCTTAATCGCGTATCAGGAATCAT<br>TGACCGAGATCGGTTTTAACACCAGGGAAATAACGAGGATGGCAGCAGCTGCTCT<br>GGTGTCATAA	This study	
crtE	Gene	ATGGACTACGCTAACATCTTGACTGCCATTCCTTTGGAATTCACCCCACAAGATGA<br>CATTGTCTTGTTGGAACCATACCACTACTTAGGTAAGAACCCAGGTAAGGAAATCA<br>GATCTCAATTGATTGAAGCTTTCAACTACTGGTTAGATGTCAAGAAGGAAGACTTG<br>GAAGTTATCCAAAATGTTGTTGGTATGTTGCACACCGCTTCTTTGTTGATGGATGAT<br>GTTGAAGATTCTTCCGTCTTGAGAAGAGGTTCTCCAGTTGCTCATTTGATCTACGG<br>TATTCCACAAACCATCAACACTGCTAACTACGTTTACTTCTTGGCTTACCAAGAAAT<br>CTTCAAATTGCGTCCAACTCCAATTCCAATGCCAGTTATCCCACCATCTTCTGCTTC<br>TTTGCAATCTTCTGTCTCCTCCGCCTCCTCTTCCTCTTCTGCCTCCTCTGAAAACGG<br>TGGTACCTCCACTCCAAACTCCCAAATCCCATTCTCCAAGGACACCTACTTGGACA<br>AGGTTATCACTGACGAAATGTTGTCTTTGCACCGTGGTCAAGGTTTGGAATTATTCT<br>GGAGAGACTCTTTGACCTGTCCATCTGAAGAAGAATACGTCAAGATGGTCTTGGGT<br>AAGACCGGTGGTTTGTTCAGAATTGCTGTCAGATTGATGATGGCCAAGTCTGAATG<br>TGACATTGACTTTGTTCAATTGGTTAACTTGATTTCCATCTACTTCCAAATCAGAGA<br>TGACTACATGAACTTGCAATCCTCTGAATACGCTCACAACAAGAACTTCGCTGAAG<br>ACTTGACTGAAGGTAAGTTCTCCTTCCCAACCATTCACTCCATTCACGCTAACCCAT<br>CTTCCAGATTGGTTATCAACACTTTACAAAAGAAGTCCACTTCTCCAGAAATCTTAC<br>ATCACTGTGTCAACTACATGAGAACTGAAACCCACTCTTTCGAATACACTCAAGAA<br>GTCTTGAACACTTTATCTGGTGCTTTGGAAAGAGAATTGGGTAGATTACAAGGTGA<br>ATTTGCTGAAGCTAACTCCAAGATCGATTTGGGTGACGTTGAATCTGAAGGTAGAA<br>CCGGTAAGAACGTCAAATTGGAAGCCATCTTGAAGAAATTGGCTGATATCCCTCTA	Verwaal, 2018	[2]
crtYB	Gene	ATGACCGCTTTGGCTTACTACCAAATCCACTTGATCTACACTTTGCCAATCTTAGGT<br>TTGCTAGGTTTGTTGACTTCTCCAATTTTGACCAAATTCGACATCTACAAGATTTCT<br>ATCTTAGTCTTTATTGCTTTCTCTGCTACCACTCCATGGGACTCCTGGATCATCAGA<br>AACGGTGCCTGGACCTACCCATCTGCTGAATCTGGTCAAGGTGTTTTCGGTACCTT<br>TTTGGATGTCCCATACGAAGAATACGCCTTCTTTGTTATCCAAACCGTCATCACCG<br>GTTTGGTTTACGTTTTGGCTACCAGACATTTGTTGCCATCTTTGGCTCTACCAAAGA<br>CCCGTTCTTCTGCCTTGTCTCTAGCTTTGAAGGCTTTAATCCCATTGCCAATCATCT<br>ATTTGTTCACCGCTCATCCATCTCCATCCCCAGATCCTTTGGTTACTGACCACTACT<br>TCTACATGAGAGCTTTGTCTTTGTTGATCACCCCACCAACCATGTTGTTGGCTGCTT<br>TATCTGGTGAATACGCTTTCGACTGGAAATCTGGTAGAGCTAAGTCCACCATTGCT<br>GCCATCATGATCCCAACTGTCTACTTGATCTGGGTTGACTACGTTGCCGTTGGTCA<br>AGACTCCTGGTCCATCAACGATGAAAAGATTGTCGGTTGGAGATTAGGTGGTGTCT<br>TGCCAATTGAAGAAGCTATGTTCTTCTTATTGACCAACTTGATGATCGTTTTGGGTT<br>TGTCTGCCTGTGACCACACTCAAGCCTTGTACTTGTTGCACGGTAGAACTATCTAC<br>GGTAACAAGAAGATGCCATCTTCTTTCCCATTAATCACTCCACCAGTTTTGTCCTTG<br>TTCTTCTCCTCCAGACCATACTCCTCCCAACCAAAGAGAGATTTGGAATTGGCTGT<br>CAAGTTGTTGGAAAAGAAGTCCAGATCTTTCTTCGTTGCTTCTGCCGGTTTCCCAT<br>CTGAAGTCAGAGAAAGATTGGTTGGTTTGTACGCTTTCTGTCGTGTCACCGATGAC<br>TTGATTGACTCTCCAGAAGTTTCCTCCAACCCACACGCTACCATTGACATGGTTTC<br>CGATTTCTTGACTTTATTATTCGGTCCTCCATTGCACCCATCTCAACCAGACAAGAT<br>TTTGTCTTCTCCATTATTACCACCTTCCCACCCATCCAGACCAACTGGTATGTACCC<br>ATTACCACCACCTCCATCTTTGTCTCCAGCTGAATTGGTCCAATTCTTGACTGAACG<br>TGTCCCAGTTCAATACCACTTCGCTTTCAGATTGTTGGCCAAATTGCAAGGTTTGAT<br>TCCAAGATACCCATTGGATGAATTATTGAGAGGTTACACCACTGACTTGATCTTCC<br>CATTGTCCACTGAAGCCGTCCAAGCTAGAAAGACCCCAATTGAAACTACTGCTGAC<br>TTGTTGGACTACGGTTTGTGTGTTGCCGGTTCTGTTGCTGAATTGTTGGTCTACGT<br>TTCCTGGGCTTCCGCTCCATCCCAAGTTCCAGCTACTATTGAAGAAAGAGAAGCTG<br>TTTTGGTCGCCTCTCGTGAAATGGGTACCGCTTTGCAATTGGTCAACATTGCCAGA<br>GATATCAAGGGTGACGCTACTGAAGGTAGATTCTACTTGCCATTGTCTTTCTTTGG<br>TTTGAGAGATGAATCCAAATTGGCCATTCCAACTGACTGGACTGAACCAAGACCTC<br>AAGATTTCGACAAATTGTTGTCTCTATCTCCATCTTCCACTTTACCATCCTCTAACG<br>CTTCTGAATCCTTCAGATTCGAATGGAAGACCTACTCTTTGCCATTGGTTGCTTACG<br>CTGAAGATTTGGCTAAGCACTCTTACAAGGGTATTGACAGATTACCAACTGAAGTC<br>CAAGCTGGTATGAGAGCTGCTTGTGCTTCTTACTTGTTGATTGGTCGTGAAATCAA<br>GGTTGTCTGGAAGGGTGATGTCGGTGAAAGAAGAACCGTTGCTGGTTGGAGAAGA<br>GTCAGAAAGGTTTTGTCTGTTGTCATGTCCGGTTGGGAAGGTCAATAA	Verwaal et al., Addgene, Plasmid #101748	[2]

hyg	Gene	ATGGGTAAAAAGCCTGAACTCACCGCGACGTCTGTCGAGAAGTTTCTGATCGAAAA<br>GTTCGACAGCGTCTCCGACCTGATGCAGCTCTCGGAGGGCGAAGAATCTCGTGCT<br>TTCAGCTTCGATGTAGGAGGGCGTGGATATGTCCTGCGGGTAAATAGCTGCGCCG<br>ATGGTTTCTACAAAGATCGTTATGTTTATCGGCACTTTGCATCGGCCGCGCTCCCG<br>ATTCCGGAAGTGCTTGACATTGGGGAATTCAGCGAGAGCCTGACCTATTGCATCTC<br>CCGCCGTGCACAGGGTGTCACGTTGCAAGACCTGCCTGAAACCGAACTGCCCGCT<br>GTTCTGCAGCCGGTCGCGGAGGCAATGGATGCGATCGCTGCGGCCGATCTTAGC<br>CAGACGAGCGGGTTCGGCCCATTCGGACCGCAAGGAATCGGTCAATACACTACAT<br>GGCGTGATTTCATATGCGCGATTGCTGATCCCCATGTGTATCACTGGCAAACTGTG<br>ATGGACGACACCGTCAGTGCGTCCGTCGCGCAGGCTCTCGATGAGCTGATGCTTT<br>GGGCCGAGGACTGCCCCGAAGTCCGGCACCTCGTGCACGCGGATTTCGGCTCCA<br>ACAATGTCCTGACGGACAATGGCCGCATAACAGCGGTCATTGACTGGAGCGAGGC<br>GATGTTCGGGGATTCCCAATACGAGGTCGCCAACATCTTCTTCTGGAGGCCGTGG<br>TTGGCTTGTATGGAGCAGCAGACGCGCTACTTCGAGCGGAGGCATCCGGAGCTTG<br>CAGGATCGCCGCGGCTCCGGGCGTATATGCTCCGCATTGGTCTTGACCAACTCTA<br>TCAGAGCTTGGTTGACGGCAATTTCGATGATGCAGCTTGGGCGCAGGGTCGATGC<br>GACGCAATCGTCCGATCCGGAGCCGGGACTGTCGGGCGTACACAAATCGCCCGC<br>AGAAGCGCGGCCGTCTGGACCGATGGCTGTGTAGAAGTACTCGCCGATAGTGGAA<br>ACCGACGCCCCAGCACTCGTCCGAGGGCAAAGGAATAA	Young, 2018	[1]
crtl	Gene	ATGGGTAAGGAACAAGACCAAGACAAGCCAACTGCCATCATCGTTGGTTGTGGTAT<br>CGGTGGTATTGCTACCGCTGCCAGATTAGCTAAGGAAGGTTTCCAAGTTACCGTCT<br>TTGAAAAGAACGACTACTCCGGTGGTAGATGTTCTTTGATTGAAAGAGATGGTTAC<br>AGATTCGACCAAGGTCCATCTTTGTTGCTATTACCAGACTTGTTCAAGCAAACCTTC<br>GAAGATTTGGGTGAAAAGATGGAAGACTGGGTTGATTTGATCAAGTGTGAACCAAA<br>CTACGTTTGTCACTTCCATGATGAAGAAACTTTCACCTTCTCCACTGACATGGCTTT<br>ATTGAAGAGAGAAGTCGAAAGATTTGAAGGTAAAGATGGTTTCGACAGATTCTTGT<br>CTTTCATCCAAGAAGCTCACAGACATTACGAATTGGCTGTTGTCCACGTCTTGCAA<br>AAGAACTTCCCAGGTTTCGCTGCTTTCTTGAGATTACAATTCATCGGTCAAATCTTA<br>GCTTTGCACCCATTTGAATCCATCTGGACCAGAGTTTGTCGTTACTTCAAGACTGA<br>CAGATTGAGAAGAGTCTTCTCCTTTGCCGTTATGTACATGGGTCAATCTCCATACT<br>CTGCTCCAGGTACCTACTCCTTGTTGCAATACACTGAATTGACTGAAGGTATCTGG<br>TACCCAAGAGGTGGTTTCTGGCAAGTTCCAAACACTTTGTTGCAAATCGTCAAGAG<br>AAACAACCCATCTGCTAAGTTCAACTTCAACGCTCCAGTTTCTCAAGTTTTGTTGTC<br>TCCAGCTAAGGACAGAGCTACCGGTGTCAGATTAGAATCTGGTGAAGAACACCAC<br>GCTGATGTTGTCATTGTCAATGCTGACTTGGTCTACGCTTCTGAACATTTGATTCCA<br>GATGATGCTAGAAACAAGATCGGTCAATTAGGTGAAGTTAAGCGTTCCTGGTGGG<br>CTGATTTGGTTGGTGGTAAGAAGTTGAAGGGTTCTTGTTCTTCTTTGTCTTTCTACT<br>GGTCTATGGACAGAATCGTTGACGGTTTGGGTGGTCACAACATCTTCTTGGCTGAA<br>GACTTCAAGGGTTCCTTCGACACCATTTTCGAAGAATTGGGTTTGCCAGCTGACCC<br>ATCTTTCTATGTTAACGTTCCATCCAGAATTGACCCTTCTGCTGCTCCAGAAGGTAA<br>GGATGCCATTGTCATCTTAGTCCCATGTGGTCACATCGATGCTTCCAACCCTCAAG<br>ACTACAACAAATTGGTTGCCAGAGCCAGAAAGTTCGTCATCCAAACCTTGTCTGCC<br>AAGTTGGGTCTACCAGATTTCGAAAAGATGATTGTTGCTGAAAAGGTTCACGATGC<br>TCCATCCTGGGAAAAGGAATTCAACTTGAAGGACGGTTCCATTTTGGGTTTGGCTC<br>ACAACTTCATGCAAGTCTTGGGTTTCAGACCATCCACCAGACACCCAAAGTACGAC<br>AAATTGTTCTTTGTCGGTGCTTCTACCCACCCAGGTACTGGTGTTCCAATTGTCTTG<br>GCTGGTGCCAAATTGACTGCTAACCAAGTTTTGGAATCCTTCGATCGTTCTCCAGC<br>TCCAGATCCTAACATGTCTTTGTCTGTTCCATACGGTAAGCCATTGAAATCCAACG<br>GTACTGGTATTGACTCTCAAGTCCAATTGAAATTCATGGACTTGGAACGTTGGGTT<br>TACCTATTAGTCTTGTTGATTGGTGCTGTTATCGCCAGATCCGTCGGTGTCTTGGC	Verwaal, 2018	[2]

dCas9	Gene	GATAAGAAATACTCTATTGGTTTGGCTATCGGTACAAACTCTGTTGGTTGGGCTGT<br>TATTACTGATGAATACAAGGTTCCATCCAAGAAGTTCAAGGTTTTGGGTAACACTGA<br>TAGACACTCCATCAAAAAGAACTTGATTGGTGCCTTGTTGTTCGATTCTGGTGAAA<br>CTGCTGAAGCTACTAGATTGAAAAGAACCGCTAGAAGAAGATACACCAGAAGAAAG<br>AACAGAATCTGCTACTTGCAAGAAATCTTCTCCAACGAAATGGCCAAGGTTGATGA<br>TTCATTCTTCCACAGATTGGAAGAATCCTTCTTGGTCGAAGAAGATAAGAAGCACG<br>AAAGACATCCAATCTTCGGTAACATCGTTGATGAAGTTGCTTACCACGAAAAGTAC<br>CCAACTATCTACCATTTGAGAAAGAAGTTGGTTGACTCTACCGATAAGGCTGATTT<br>GAGATTGATCTATTTGGCTTTGGCCCACATGATTAAGTTCAGAGGTCATTTCTTGAT<br>CGAAGGTGATTTGAACCCAGATAACTCCGATGTTGATAAGTTGTTCATCCAATTAGT<br>CCAAACCTACAATCAATTATTCGAAGAAAACCCAATCAACGCCTCTGGTGTTGATG<br>CTAAAGCTATTTTGTCTGCCAGATTGTCCAAGTCCAGAAGATTAGAAAATTTGATCG<br>CCCAATTACCAGGTGAAAAGAAGAATGGTTTGTTCGGTAATTTGATTGCCTTGTCTT<br>TGGGTTTGACTCCAAACTTCAAGTCCAATTTCGATTTGGCTGAAGATGCCAAGTTG<br>CAATTATCTAAGGATACCTACGATGACGATTTGGATAACTTGTTGGCTCAAATCGGT<br>GATCAATACGCTGATTTGTTTTTGGCTGCTAAGAACTTGTCCGATGCCATTTTGTTG<br>TCCGATATTTTGAGAGTCAACACCGAAATTACTAAGGCTCCATTGTCTGCCTCTATG<br>ATCAAAAGATACGATGAACACCACCAAGACTTGACTTTGTTGAAGGCTTTGGTCAG<br>ACAACAATTACCTGAAAAGTACAAAGAAATTTTCTTCGATCAATCCAAGAACGGTTA<br>CGCCGGTTATATTGATGGTGGTGCTTCTCAAGAAGAATTTTACAAGTTCATCAAGC<br>CAATCTTGGAAAAGATGGACGGTACTGAAGAATTATTGGTCAAGTTGAACAGAGAA<br>GATTTGTTGAGAAAGCAAAGAACCTTCGACAACGGTTCTATTCCACATCAAATTCAC<br>TTGGGTGAATTGCACGCAATTTTGAGAAGACAAGAAGATTTTTATCCATTCTTGAAG<br>GACAACAGAGAAAAGATCGAAAAGATTCTGACCTTCAGAATCCCTTACTACGTTGG<br>TCCATTGGCTAGAGGTAATTCAAGATTTGCCTGGATGACTAGAAAGTCCGAAGAAA<br>CTATTACTCCTTGGAACTTCGAAGAAGTTGTAGATAAGGGTGCTTCTGCCCAATCC<br>TTTATTGAAAGAATGACCAACTTCGACAAGAACTTGCCAAACGAAAAGGTTTTGCC<br>AAAGCACTCTTTGTTGTACGAATACTTCACCGTCTACAACGAATTGACTAAGGTTAA<br>GTACGTCACCGAAGGTATGAGAAAACCAGCTTTTTTATCCGGTGAACAAAAGAAGG<br>CTATCGTCGATTTGTTGTTCAAGACCAACAGAAAGGTTACTGTCAAGCAATTAAAAG<br>AAGATTACTTCAAGAAAATCGAATGCTTCGACTCCGTTGAAATTTCTGGTGTCGAA<br>GATAGATTCAATGCCTCTTTAGGTACTTACCATGACTTGTTGAAAATCATCAAGGAC<br>AAGGATTTCTTGGACAACGAAGAAAACGAAGATATTTTGGAAGATATTGTCTTGACA<br>TTGACCTTGTTTGAAGATAGAGAAATGATTGAAGAAAGATTGAAAACCTACGCCCA<br>CTTGTTCGATGATAAGGTTATGAAGCAATTAAAGAGAAGAAGATACACTGGTTGGG<br>GTAGATTGTCCAGAAAATTGATTAACGGTATCAGAGACAAGCAATCCGGTAAGACC<br>ATTTTGGACTTTTTGAAGTCTGATGGTTTCGCTAACAGAAACTTCATGCAATTAATC<br>CACGACGATTCCTTGACTTTCAAAGAAGATATACAAAAGGCCCAAGTCTCTGGTCA<br>AGGTGATTCTTTACATGAACATATCGCTAACTTGGCTGGTTCTCCAGCTATTAAGAA<br>GGGTATTTTACAAACCGTTAAGGTCGTTGACGAATTGGTCAAAGTTATGGGTAGAC	Farzadfard, 2013	[8]
kan	Gene	ATGGGTAAGGAAAAGACACACGTTTCGAGGCCGCGATTAAATTCCAACATGGATG<br>CTGATTTATATGGGTATAAATGGGCTCGCGATAATGTCGGGCAATCAGGTGCGACA<br>ATCTATCGATTGTATGGGAAGCCCGATGCGCCAGAGTTGTTTCTGAAACATGGCAA<br>AGGTAGCGTTGCCAATGATGTTACAGATGAGATGGTCAGACTAAACTGGCTGACG<br>GAATTTATGCCTCTTCCGACCATCAAGCATTTTATCCGTACTCCTGATGATGCATGG<br>TTACTCACCACTGCGATCCCCGGCAAAACAGCATTCCAGGTATTAGAAGAATATCC<br>TGATTCAGGTGAAAATATTGTTGATGCGCTGGCAGTGTTCCTGCGCCGGTTGCATT<br>CGATTCCTGTTTGTAATTGTCCTTTTAACAGCGATCGCGTATTTCGTCTCGCTCAGG<br>CGCAATCACGAATGAATAACGGTTTGGTTGATGCGAGTGATTTTGATGACGAGCGT<br>AATGGCTGGCCTGTTGAACAAGTCTGGAAAGAAATGCATAAGCTTTTGCCATTCTC<br>ACCGGATTCAGTCGTCACTCATGGTGATTTCTCACTTGATAACCTTATTTTTGACGA<br>GGGGAAATTAATAGGTTGTATTGATGTTGGACGTGTCGGAATCGCAGACCGATACC<br>AGGATCTTGCCATCCTATGGAACTGCCTCGGTGAGTTTTCTCCTTCATTACAGAAA<br>CGGCTTTTTCAAAAATATGGTATTGATAATCCTGATATGAATAAATTGCAGTTTCATT<br>TGATGCTCGATGAGTTTTTCTAAA	Young, 2018	[1]
yEmCitrine	Gene	ATGTCTAAAGGTGAAGAATTATTCACTGGTGTTGTCCCAATTTTGGTTGAATTAGAT<br>GGTGATGTTAATGGTCACAAATTTTCTGTCTCCGGTGAAGGTGAAGGTGATGCTAC<br>TTACGGTAAATTGACCTTAAAATTTATTTGTACTACTGGTAAATTGCCAGTTCCATG<br>GCCAACCTTAGTCACTACTTTAGGTTATGGTTTGATGTGTTTTGCTAGATACCCAGA<br>TCATATGAAACAACATGACTTTTTCAAGTCTGCCATGCCAGAAGGTTATGTTCAAGA<br>AAGAACTATTTTTTTCAAAGATGACGGTAACTACAAGACCAGAGCTGAAGTCAAGTT<br>TGAAGGTGATACCTTAGTTAATAGAATCGAATTAAAAGGTATTGATTTTAAAGAAGA<br>TGGTAACATTTTAGGTCACAAATTGGAATACAACTATAACTCTCACAATGTTTACAT<br>CATGGCTGACAAACAAAAGAATGGTATCAAAGTTAACTTCAAAATTAGACACAACAT<br>TGAAGATGGTTCTGTTCAATTAGCTGACCATTATCAACAAAATACTCCAATTGGTGA<br>TGGTCCAGTCTTGTTACCAGACAACCATTACTTATCCTATCAATCTAGATTATCCAA<br>AGATCCAAACGAAAAGAGGGATCACATGGTCTTGTTAGAATTTGTTACTGCTGCTG<br>GTATTACCCATGGTATGGATGAATTGTACAAATAA	Young, 2018	[1]

dCas9Mx1	Gene	ATGTCTAGAGCCCCAAAGAAGAAGAGAAAAGTTAGACCCGGGGATAAGAAATACT<br>CTATTGGTTTGGCTATCGGTACAAACTCTGTTGGTTGGGCTGTTATTACTGATGAAT<br>ACAAGGTTCCATCCAAGAAGTTCAAGGTTTTGGGTAACACTGATAGACACTCCATC<br>AAAAAGAACTTGATTGGTGCCTTGTTGTTCGATTCTGGTGAAACTGCTGAAGCTAC<br>TAGATTGAAAAGAACCGCTAGAAGAAGATACACCAGAAGAAAGAACAGAATCTGCT<br>ACTTGCAAGAAATCTTCTCCAACGAAATGGCCAAGGTTGATGATTCATTCTTCCACA<br>GATTGGAAGAATCCTTCTTGGTCGAAGAAGATAAGAAGCACGAAAGACATCCAATC<br>TTCGGTAACATCGTTGATGAAGTTGCTTACCACGAAAAGTACCCAACTATCTACCAT<br>TTGAGAAAGAAGTTGGTTGACTCTACCGATAAGGCTGATTTGAGATTGATCTATTTG<br>GCTTTGGCCCACATGATTAAGTTCAGAGGTCATTTCTTGATCGAAGGTGATTTGAA<br>CCCAGATAACTCCGATGTTGATAAGTTGTTCATCCAATTAGTCCAAACCTACAATCA<br>ATTATTCGAAGAAAACCCAATCAACGCCTCTGGTGTTGATGCTAAAGCTATTTTGTC<br>TGCCAGATTGTCCAAGTCCAGAAGATTAGAAAATTTGATCGCCCAATTACCAGGTG<br>AAAAGAAGAATGGTTTGTTCGGTAATTTGATTGCCTTGTCTTTGGGTTTGACTCCAA<br>ACTTCAAGTCCAATTTCGATTTGGCTGAAGATGCCAAGTTGCAATTATCTAAGGATA<br>CCTACGATGACGATTTGGATAACTTGTTGGCTCAAATCGGTGATCAATACGCTGAT<br>TTGTTTTTGGCTGCTAAGAACTTGTCCGATGCCATTTTGTTGTCCGATATTTTGAGA<br>GTCAACACCGAAATTACTAAGGCTCCATTGTCTGCCTCTATGATCAAAAGATACGA<br>TGAACACCACCAAGACTTGACTTTGTTGAAGGCTTTGGTCAGACAACAATTACCTG<br>AAAAGTACAAAGAAATTTTCTTCGATCAATCCAAGAACGGTTACGCCGGTTATATTG<br>ATGGTGGTGCTTCTCAAGAAGAATTTTACAAGTTCATCAAGCCAATCTTGGAAAAG<br>ATGGACGGTACTGAAGAATTATTGGTCAAGTTGAACAGAGAAGATTTGTTGAGAAA<br>GCAAAGAACCTTCGACAACGGTTCTATTCCACATCAAATTCACTTGGGTGAATTGC<br>ACGCAATTTTGAGAAGACAAGAAGATTTTTATCCATTCTTGAAGGACAACAGAGAAA<br>AGATCGAAAAGATTCTGACCTTCAGAATCCCTTACTACGTTGGTCCATTGGCTAGA<br>GGTAATTCAAGATTTGCCTGGATGACTAGAAAGTCCGAAGAAACTATTACTCCTTG<br>GAACTTCGAAGAAGTTGTAGATAAGGGTGCTTCTGCCCAATCCTTTATTGAAAGAA<br>TGACCAACTTCGACAAGAACTTGCCAAACGAAAAGGTTTTGCCAAAGCACTCTTTG<br>TTGTACGAATACTTCACCGTCTACAACGAATTGACTAAGGTTAAGTACGTCACCGA<br>AGGTATGAGAAAACCAGCTTTTTTATCCGGTGAACAAAAGAAGGCTATCGTCGATT<br>TGTTGTTCAAGACCAACAGAAAGGTTACTGTCAAGCAATTAAAAGAAGATTACTTCA<br>AGAAAATCGAATGCTTCGACTCCGTTGAAATTTCTGGTGTCGAAGATAGATTCAAT<br>GCCTCTTTAGGTACTTACCATGACTTGTTGAAAATCATCAAGGACAAGGATTTCTTG<br>GACAACGAAGAAAACGAAGATATTTTGGAAGATATTGTCTTGACATTGACCTTGTTT<br>GAAGATAGAGAAATGATTGAAGAAAGATTGAAAACCTACGCCCACTTGTTCGATGA<br>TAAGGTTATGAAGCAATTAAAGAGAAGAAGATACACTGGTTGGGGTAGATTGTCCA<br>GAAAATTGATTAACGGTATCAGAGACAAGCAATCCGGTAAGACCATTTTGGACTTT<br>TTGAAGTCTGATGGTTTCGCTAACAGAAACTTCATGCAATTAATCCACGACGATTCC<br>TTGACTTTCAAAGAAGATATACAAAAGGCCCAAGTCTCTGGTCAAGGTGATTCTTTA<br>CATGAACATATCGCTAACTTGGCTGGTTCTCCAGCTATTAAGAAGGGTATTTTACAA	Farzadfard, 2013	[8]
pst	Gene	ATGTCCGATTCCGTTGAAGTAGGGTTCAACAAGCTCCGATTCACCTCTTTCACCTC<br>CTTCGGAGATGAGTTCATCAACGAGCACGAAGCTCCTGCTTTCATCGAATCTGTTG<br>CCTGGTTCCAGTCCCTTAACGCTATTGCTACTCCTCAGCACCTCAAGATCGTCAAA<br>AACGCTACCTTCGAGCGACTCGTTTCTCGAACTTTCCCTTTCGCTGATCTCGCTGG<br>AGCTAGAATTGCTACCGATCTCATGATCCTCACCTTCCTCATCGATGATCTCTCCG<br>ATGTCGTCGAAGCTACCGATGATACTGCTATGCACGCTATGTCTGCTGTCGAAGGA<br>CAGGTTACCCATGTTCTTAGAGGAGGAACTCCTAGACCTGGAGAACATCCTCTTGC<br>TGTTGCTATGCGATCCATCGTTGATCGAGCTATGCTCACCTACAACCCTGATTGGA<br>TCGACCTTATGCGGAAGGAGTTCATCACCTACCTCGAGATGAACCGACTTGAGCG<br>AATCAACCGACTTGAAGGACCTGGACTTTCTTGGACCATGTTCGAAAACACCCGGT<br>ACTACTCCTCTTGCGTTCTTCCTTTCCTCTACCTCTCTGCTGGAATGGGATGTACTG<br>GATGTCCTTCTACCGTCCTTTCTGTCCCTTTCGTCAAGATCATGACCGATCTTACC<br>GTCAACCACGTTGCTTGGGTCAACGATATCGTTGGAGCCAACAAGGAGCGAAAGG<br>AAGCTGTTAACAACAACATCGTGTTCGTCATCGCCAACGATCGAGGACTTACTATG<br>GCTGGAGCTGTCAAAGATGCCGTTAAGCGAACTAACCAGGAGTGTGAAGTGTTCC<br>TCAACCTTGAACACCGACTTCATGCTGGAGGAGCTGTTGTTGATGGAGATGATCTC<br>TTCAACTACATCGAGGTCCTCAAGTACTGGATGCGAGGATCTCTTGATTGGCACTT<br>CGAGTCTAAGCGGTACAAGGTTAAGGCCTCTTAA	This study	

mdst	Gene	ATGGCTTCCAACTCTCCTACCCTCGAAAACACCAAGCTTGCTGGAGCTCCTGAAAA<br>GCGACAGAAGAAGAAGCAGCTCCCTTATCAGGGAATCCTTCATGTTCCTGGGGAC<br>CGAGTCGAAGAGCTTGATACTCGAGAAACCTCTCTCCTTGTCGCTGAAGTTAAGGG<br>ATGGCTCATGAAGCTCGCTTCTGGAAAAGGAGAGATCTCCCCTTCTGCTTACGATA<br>CTGCTTGGGTTGCTCGAATCGCTTCTGAGTCTGATTCCTCTCTTCCCGAATTCCCT<br>GAGGCTTTAGAGTGGATCATCAACTCTCAGCTCCCTGATGGATCTTGGGGAGATG<br>ATCGACATCTTCAGCTCTACGATCGAGTCCTTTCTACCCTTTCTTGCCTTGTCACCC<br>TCAAGACTTGGGATATCGGACACAACTCCATCGCTCAAGGAACCAAGTTCCTTCGA<br>GAGAACATGATCAAGCTCAAGCAGGATGACGGAGATCTCCTTTCTGGATTCGAAGT<br>CACCTTCCCCATGATGCTTCACGAAGCTAAGCAGCTTGGACTCGATATCCCTTACG<br>AAACCGAGTTTACCCGACTTCTCGAGATCTCTACCAAGAAGAAGCTCGCCAAGATC<br>CCTCTCGATAAGCTTCATTCTGCCCCTACTACCCTCCTCTACTCTTTAGAAGGACTC<br>CAGGATCTCGAGATCGATTGGCAAAAGATCCTCAAGCTCCAGTCCAAGGATGGAT<br>CTTTCCTCTCTTCCCCTTCTTCTACCGCTTGTGTCTACCTTAAGACCAAGGACCGAA<br>AGTCTCTCCAGTACCTTCAGAACGCTATGGAGGATCAGAACTACGCTGTCCCTTGC<br>CATTACCCTATCGATCTTTTCGAGTCCCTCTGGGTCGTTGATACCATCGAAAGACT<br>CGGAATCGACGTATTCTTCCGAGATGAGATCAAGGCTGTCCTCGATTACGTCTACT<br>CCTTTTGGACCAACGAGGGAATTGGATGGGGATCTACCTGTCTTGTCAACGATATC<br>GACGATACCGCTATGGCTTTCCGAATCCTTCGAATGCACGGCTACAACGTTTCCCC<br>TGATGCTTTTAACCAGTTCTGGCTTCCTGGCGACAAGTTTTGCTGCTTTGTCGGAG<br>AGCTCTCTCATGGAGTCTCTGAAATGCTCAACCTCCATCGAGCTTCTCAGGTCGAT<br>TTCCCTAACGAAGCTATCCTCACCAAGACCTTCAAGTACTCCCACGATTACCTCCT<br>CAACGTCGATTCTGCTCACATGGATAAGTGGGCTACCAAGAAGAACCTCATGGGA<br>GAAGTCGCTTTCGAGCTTGCTAACCCTTTCCATGATTGCCTTCCCCGAATCTACAA<br>CAACGCCTACATCAAGCACTACGGCATGGATGATCTCTGGATCGCTAAGACCATCT<br>ACCGACTTCCTCTCGTCAACAACAAGGTATTCCTCGAACTCGCTAACCGATATGCT<br>CAGCAGTGTCAGCTTTACCAACCTGCTGAACTCACCAAGCTCGTTAATTGGTGGCA<br>CTCTTCCCGATTCGAGGATATTCCTTCTACCCGACTTACCGCCAACATCGATATGC<br>TTCCCTACATCTACTACGTCATCTGCGCTACCTTCCACGAACAGGAATTTGCTCAG<br>CTTCGGGTATTCTTCTCCAAGGCTTGTTGCCTTAACACCCTCTTCGACGATCTCAT<br>GGATTGTGCTACCTCCATCGAGGAGCTTGATCGACTTCAGAACGTCATCGAACGAT<br>GGGATATCTCTCTCTCTCACGAGCTTCCTCTCGAATACAGAATCCCTTTCCAGGAG<br>TTCTACAACACCGTCCTTGTCATGACCGAAGCTGCTTCTAAGATCCACAAGAACCT<br>CTCTCCCGAGTTCATCTGCAAGTACCTTTCCGGAATCTACACCAAGCTCATCAAGT<br>CCGAGATCGCTGATGCTAGATGGAAGATCGAGGGCTATATCCCTTCCTTCGAGGA<br>GTACATGGAGAACGCTGAAGTCTCTATCTCCACCTGGGTCCATGTCCTTATGTCTA<br>TCCTCTTCTGCGGAGAGCCTCTTACCGAAGAAATCCTTAACACCATCTACGACTCC<br>CGACCTCTTAAGCTTGACCGAATCATCTGCCGACTCTGCAACGATATCCAGACCTA<br>CAAGATCGAGATGAAGCTCGGACAGCCTACTCAAGGAGTCTCTTGTTACATGAAG<br>GAGCACCCTGGAGCTACCGAAGAAGATGCTCTTGTCTACCTCCAGTCTCTCCTTGA	This study	
hfst	Gene	ATGTCCTCTCCTGAAATGGCCAAAGAGATCATGCACAAGTACGGACAGGTGTTCTC<br>TGGACGAACTATCGCTCAAGCTGTCCATGCTTGTGATCACGACAAGATCTCCATGG<br>GATTCCTTCCTGTTGGAGCTGAATGGCGAGATATGCGAAAGATCTGCAAGGAGCA<br>GATGTTCTCTCACCAGTCTATGGAGGATTCCCAGAACCTTCGAAAGCAGAAGCTTC<br>AGCAGCTCCTCGATTATACCCAGAAGTGCTCTGAAGAGGGACGAGGAATCGATAT<br>CCGAGAAGCTGCTTTCATCACCACCCTTAACCTCATGTCTGCTACCCTCTTCTCCA<br>TGCAAGCTACCGAATTCGACTCCAAGGTCACCATGGAGTTCAAGGAGATCATCGAA<br>GGAGTCGCTTCCATCGTTGGAGTTCCTAACTTCGCTGACTACTTCCCTATCCTTCG<br>ACCTTTCGATCCTCAGGGAGTTAAGAGACGAGCTGATGTCTACTTCGGACGACTTC<br>TTGGACTCATCGAGGGATACCTTAACGAGCGAATCGAGTTCCGAAAGGCTAACCC<br>TAATGCCCCTAAGAAGGACGATTTCCTTGAAACCCTCGTCGATGCTCTTGATGCTA<br>AGGACTACAAGCTCAAGACCGAGCATCTTACCCACCTTATGCTCGATCTCTTCGTT<br>GGAGGATCTGAGACTTCTACCACCGAAATCGAGTGGATCATGTGGGAACTTGTCG<br>CTTCTCCTGAGAAGATGGCTAAGGTTAAGGCTGAGCTCAAGTCCGTTATGGGAGG<br>AGAAAAGGTCGTCGATGAGTCCATGATGCCTAGACTTCCTTACCTTCAGGCTGTCG<br>TCAAGGAGTCTATGCGATTACATCCTCCTGGACCTCTCCTTCTTCCTAGAAAAGCT<br>GAGTCCGATCAGGTCGTTAACGGATACCTTATCCCCAAGGGAACCCAGGTTCTCAT<br>TAATGCTTGGGCTATGGGACGAGATTCCTCTCTTTGGAAGAACCCTGACTCCTTCG<br>AGCCTGAACGATTCCTTGATCAGAAGATCGACTTCAAGGGCACCGATTACGAGCTT<br>ATCCCTTTCGGATCTGGACGACGAGTTTGTCCTGGAATGCCTCTTGCTAACCGAAT<br>CCTTCATACCGTTACCGCTACCCTTGTCCACAACTTTGACTGGAAGCTTGAGCGAC<br>CTGAGGCTAATGATGCTCATAAGGGAGTCCTCTTCGGATTTGCTGTTAGACGAGCT<br>GTCCCTCTCAAGATTGTCCCTATTAAGGCCTAA	This study	

cast	Gene	ATGTGGTCTGAGTATTCCCGAGGTAGACAAGGAGGATCTGGAGGATTACCTCCTG<br>GACCTCCTAGACTTCCTATCATTGGCAACATCCTCCAGCTTGGACGAGATCCTCAC<br>AAGTCTCTTGCTCAACTCGCTAAGACCTACGGACCTCTTATGTCTCTCAAGCTCGG<br>AAACCAGTTCGCTGTTGTTGTCTCTTCTCCCGAAATGGCTCGAGAGATCCTTCAGA<br>AACAGGGACTCATCTTCTCCAAGCCTTTCACTCCTTCTGCTGTTCGAGTCCTTGGA<br>CACAACGATATCTCCATGAACATGCTCCCTGCTTCCTCTGATCGATGGAAGAAGCT<br>TCGACGAGTTGCTCGAGAACAGCTCTTCTCTAATCCTGCTCTTCAGGCTACCCAGG<br>ATATCAGACAAGAGAGACTTCGACAGCTCACCGATTACGCTTCTCGATGTTGTGCT<br>CAAGGACGAGCTATGAACGTCGGAGAAGCTACTTTTACCACCATGACCAACCTCAT<br>GTTCGCTACCCTCTTCTCTGTCGAACTTACCCAGTATGGAGCTACCGATACCGGAT<br>CTGACAAGAAGTTCAAGGAGCACGTCAACGCTCTTACCCGATATATGGGAGTCCCT<br>AACGTCGCTGACTTCTTCCCTTTTCTTGCTCCTCTCGATCCTCAGGGAATGAGAAG<br>AAAGCTCACCTACCACCTCGGATCTCTTCTTGAACTTGTCCAGTCCCTCATCCAGC<br>AACGATTACAAGCTCGAAACGACTCCACCTACCAGAAGAAGAACGACTTCCTCGAT<br>ACCCTCCTCGATCTTTCTGAGGGAAACGAGTACGATCTCTCCATCAAGGAGATCAA<br>GCACATGTTCGTCGACCTCATCATCGCTGGATCTGATACTTCTGCTGCTACCACTG<br>AATGGGCTATGGTCGAACTTCTCCTTCACCCTGACAAGATGGCTAAGCTTAAGGCT<br>GAGCTCAAGTCTGTCCTTGGAGAGAAGTCTATCGTCGAGGAGTCCGATATCTCTC<br>GACTTCCTTACCTTCTCGCTACCGTCAAGGAAGTTCTCCGATATCATCCTGCTGCT<br>CCTCTTTTAGCTCCTCATGCTGCTGAAGAAGAAACCCAAGTTTCCGGATACATCAT<br>CCCCAAGAACACCAAGATGTTCATCAACGTCTGGTCCATCACCCGAGATCCTTCTA<br>TTTGGAAGAACCCTGAGTCCTTCGAGCCTGAACGATTCCTTGATTCCGAGATCGAC<br>TTCGGAGGACAGCACTTTGAGCTTATCCCTTTCGGATCTGGACGGAGAATCTGTCC<br>TGGAATGCCTTTAGCTTCCCGAATGCTTCAGTGCATGGTTGCTACCCTTTGCCACA<br>ACTTCGATTGGGAACTCGAGAAAGGAGCTGAGTCTAAGCAGCTTCAGCGAGAAGA<br>TGTGTTCGGACTTGCTCTTCAGAAGAAGATCCCTCTTCGAGCTGTCCCTATCAAGG	This study	
nppst	Gene	ATGTCTGCTCGAGGACTCAACAAAATCTCCTGCTCTCTCAACCTCCAGACCGAAAA<br>GCTTTGCTACGAGGACAACGACAACGATCTCGATGAGGAGCTTATGCCTAAGCAC<br>ATCGCTCTCATCATGGACGGAAACAGAAGATGGGCTAAGGACAAGGGACTTGAGG<br>TCTATGAAGGCCATAAGCACATCATCCCCAAGCTTAAGGAGATCTGCGACATCTCT<br>TCCAAGCTCGGAATCCAGATCATCACCGCTTTCGCTTTCTCTACCGAGAACTGGAA<br>GCGATCTAAGGAAGAGGTCGACTTCCTTCTCCAGATGTTCGAGGAGATCTACGAC<br>GAGTTCTCTCGATCTGGAGTTCGAGTCTCCATCATCGGATGCAAGTCTGATCTCCC<br>TATGACCCTTCAGAAGTGCATCGCTCTTACCGAAGAAACCACCAAGGGCAACAAG<br>GGACTTCATCTTGTCATCGCTCTCAACTACGGCGGATACTACGATATCCTTCAGGC<br>TACCAAGTCCATCGTCAACAAGGCTATGAACGGACTCCTCGATGTCGAGGATATCA<br>ACAAGAACCTCTTCGACCAGGAGCTCGAGTCTAAATGTCCTAACCCTGATCTCCTC<br>ATCCGAACTGGAGGAGAACAACGAGTCTCCAACTTCCTTCTTTGGCAGCTTGCTTA<br>CACCGAGTTCTACTTCACCAACACCCTCTTCCCTGACTTCGGAGAAGAGGATCTTA<br>AGGAGGCCATCATGAACTTCCAGCAGAGACATCGACGATTCGGAGGACATACCTA	This study	
lst	Gene	ATGAGCAGCTGCATCAATCCATCGACCCTCGTCACCTCCGTCAACGCTTTTAAGTG<br>CCTTCCTCTCGCTACCAACAAGGCCGCAATCCGAATCATGGCTAAGTACAAGCCC<br>GTCCAGTGTCTCATTTCCGCTAAATACGATAACCTGACAGTGGATCGAAGATCGGC<br>GAATTACCAACCTTCGATCTGGGATCATGACTTTCTGCAGAGTCTGAACAGCAACT<br>ACACCGATGAGGCCTACAAGAGAAGGGCTGAGGAACTCAGGGGAAAAGTGAAGAT<br>TGCTATTAAAGACGTCATTGAACCATTGGATCAATTGGAGCTGATCGATAACTTGC<br>AGAGGTTGGGACTCGCACATCGATTCGAAACGGAAATTCGAAATATCCTGAATAAC<br>ATCTATAACAATAACAAGGACTATAACTGGCGAAAGGAAAACTTGTACGCCACCTC<br>GTTGGAATTTAGACTTTTGCGACAGCACGGATATCCTGTCAGTCAGGAGGTATTCA<br>ATGGATTTAAGGACGACCAAGGCGGTTTCATTTGTGACGACTTCAAGGGAATTCTG<br>TCCCTCCATGAGGCATCTTATTACTCCTTGGAGGGTGAATCCATCATGGAAGAGGC<br>ATGGCAGTTCACGTCCAAGCACCTTAAGGAGGTTATGATCAGTAAAAACATGGAGG<br>AGGATGTGTTCGTCGCGGAACAAGCAAAGCGAGCTCTCGAGCTTCCTCTCCACTG<br>GAAGGTTCCCATGCTCGAGGCTCGATGGTTCATCCACATCTATGAACGACGGGAG<br>GATAAGAATCACCTCTTGTTGGAACTCGCTAAGATGGAATTCAATACCCTCCAGGC<br>CATCTACCAGGAGGAGTTGAAGGAGATCTCAGGGTGGTGGAAAGATACTGGACTC<br>GGGGAGAAGCTCTCGTTCGCGAGGAACCGGCTCGTCGCTTCTTTCTTGTGGAGCA<br>TGGGAATCGCTTTCGAGCCTCAGTTCGCTTATTGCAGAAGGGTCCTCACAATCTCC<br>ATTGCTCTTATCACGGTCATTGACGACATCTACGACGTGTATGGAACCCTCGATGA<br>GTTGGAAATTTTCACTGATGCTGTTGAGCGATGGGATATCAACTACGCTTTGAAAC<br>ATCTCCCCGGATACATGAAGATGTGTTTCCTGGCCTTGTATAATTTCGTCAATGAGT<br>TTGCATATTACGTCCTTAAGCAACAGGATTTCGATTTGCTCTTGTCCATCAAAAACG<br>CGTGGCTCGGACTTATCCAGGCATACCTCGTTGAAGCCAAATGGTATCACTCGAA<br>GTATACGCCTAAGCTTGAAGAGTATCTCGAGAACGGACTCGTTTCAATCACCGGAC<br>CCCTGATTATCACTATTTCATATCTCTCCGGCACAAACCCTATCATCAAGAAAGAGC<br>TTGAATTCCTGGAATCGAATCCTGATATCGTCCACTGGAGTTCCAAGATCTTCCGA<br>CTGCAGGACGATCTTGGAACATCCTCTGACGAAATCCAGAGGGGAGACGTCCCTA<br>AGTCTATCCAGTGCTACATGCATGAGACGGGCGCCTCGGAAGAGGTCGCTCGACA<br>GCATATCAAGGATATGATGCGACAAATGTGGAAAAAGGTTAACGCATACACCGCCG<br>ACAAAGATAGTCCACTTACCGGAACAACCACCGAATTCCTTCTTAATCTTGTGAGG<br>ATGTCTCATTTTATGTACCTCCATGGTGATGGTCACGGAGTCCAGAACCAGGAAAC<br>TATTGATGTCGGCTTCACTTTGCTTTTTCAGCCTATCCCTCTCGAGGACAAGCACAT<br>GGCGTTTACCGCCTCCCCCGGAACAAAGGGCTAA	This study	

yEGFP-2A-mRuby	Gene	ATGTCTAAAGGTGAAGAATTATTCACTGGTGTTGTCCCAATTTTGGTTGAATTAGATGGTGATGTTAATGGTCACAAATTTTCTGTCTCCGGTGAAGGTGAAGGTGATGCTACTTACGGTAAATTGACCTTAAAATTTATTTGTACTACTGGTAAATTGCCAGTTCCATGGCCAACCTTAGTCACTACTTTAACTTATGGTGTTCAATGTTTTTCTAGATACCCAGATCATATGAAACAACATGACTTTTTCAAGTCTGCCATGCCAGAAGGTTATGTTCAAGAAAGAACTATTTTTTTCAAAGATGACGGTAACTACAAGACCAGAGCTGAAGTCAAGTTTGAAGGTGATACCTTAGTTAATAGAATCGAATTAAAAGGTATTGATTTTAAAGAAGATGGTAACATTTTAGGTCACAAATTGGAATACAACTATAACTCTCACAATGTTTACATCATGGCTGACAAACAAAAGAATGGTATCAAAGTTAACTTCAAAATTAGACACAACATTGAAGATGGTTCTGTTCAATTAGCTGACCATTATCAACAAAATACTCCAATTGGTGATGGTCCAGTCTTGTTACCAGACAACCATTACTTATCCACTCAATCTGCCTTATCCAAAGATCCAAACGAAAAGAGGGATCACATGGTCTTGTTAGAATTTGTTACTGCTGCTGGTATTACCCATGGTATGGATGAATTGTACAAAGGAGCGACGAACTTCAGCCTGTTGAAACTGGCTGGGGATGTCGAACTGAATCCAGGGCCTATGGTTTCCAAGGGTGAAGAATTGATCAAGGAAAACATGAGAATGAAGGTTGTCATGGAAGGTTCTGTCAACGGTCACCAATTCAAATGTACCGGTGAAGGTGAAGGTAACCCATACATGGGTACTCAAACCATGAGAATCAAGGTTATTGAAGGTGGTCCATTACCATTTGCTTTCGACATCTTGGCTACTTCTTTCATGTACGGTTCCAGAACTTTCATCAAATACCCAAAGGGTATTCCAGACTTCTTCAAGCAATCCTTCCCAGAAGGTTTCACCTGGGAAAGAGTTACCCGTTACGAGGATGGTGGTGTTGTCACCGTCATGCAAGATACCTCTTTGGAAGATGGTTGTTTGGTCTACCACGTTCAAGTCCGTGGTGTCAACTTCCCATCTAACGGTCCTGTTATGCAAAAGAAAACCAAGGGTTGGGAACCAAACACTGAAATGATGTACCCAGCTGACGGTGGTTTGAGAGGTTACACTCACATGGCTTTGAAGGTCGATGGTGGTGGTCACTTGTCTTGTTCTTTCGTCACCACTTACAGATCCAAAAAGACTGTTGGTAACATCAAGATGCCAGGTATTCATGCCGTTGACCACAGATTGGAAAGATTGGAAGAATCTGACAACGAAATGTTCGTTGTCCAAAGAGAACACGCTGTTGCCAAATTTGCTGGTTTGGGTGGTGGTATGGATGAATTATACAAGTAA	This study	
Klleu2	Gene	ATGTCTAAGAATATCGTTGTCCTACCGGGTGATCACGTCGGTAAAGAAGTTACTGACGAAGCTATTAAGGTCTTGAATGCCATTGCTGAAGTCCGTCCAGAAATTAAGTTCAATTTCCAACATCACTTGATCGGGGGTGCTGCCATCGATGCCACTGGCACTCCTTTACCAGATGAAGCTCTAGAAGCCTCTAAGAAAGCCGATGCTGTCTTACTAGGTGCTGTTGGTGGTCCAAAATGGGGTACGGGCGCAGTTAGACCAGAACAAGGTCTATTGAAGATCAGAAAGGAATTGGGTCTATACGCCAACTTGAGGCCATGTAACTTTGCTTCTGATTCTTTACTAGATCTTTCTCCTTTGAAGCCTGAATATGCAAAGGGTACCGATTTCGTCGTCGTTAGAGAATTGGTTGGTGGTATCTACTTTGGTGAAAGAAAAGAAGATGAAGGTGACGGAGTTGCTTGGGATTCTGAGAAATACAGTGTTCCTGAAGTTCAAAGAATTACAAGAATGGCTGCTTTCTTGGCATTGCAACAAAACCCACCATTACCAATCTGGTCACTTGACAAGGCTAACGTGCTTGCCTCTTCCAGATTGTGGAGAAAGACTGTTGAAGAAACCATCAAGACTGAGTTCCCACAATTAACTGTTCAGCACCAATTGATCGATTCTGCTGCTATGATTTTGGTTAAATCACCAACTAAGCTAAACGGTGTTGTTATTACCAACAACATGTTTGGTGATATTATCTCCGATGAAGCCTCTGTTATTCCAGGTTCTTTGGGTTTATTACCTTCTGCATCTCTAGCTTCCCTACCTGACACTAACAAGGCATTCGGTTTGTACGAACCATGTCATGGTTCTGCCCCAGATTTACCAGCAAACAAGGTTAACCCAATTGCTACCATCTTATCTGCAGCTATGATGTTGAAGTTATCCTTGGATTTGGTTGAAGAAGGTAGGGCTCTTGAAGAAGCTGTTAGAAATGTCTTGGATGCAGGTGTCAGAACCGGTGACCTTGGTGGTTCTAACTCTACCACTGAGGTTGGCGATGCTATCGCCAAGGCTGTCAAGGAAATCTTGGCTTAA	Young, 2018	[1]
Venus	Gene	ATGTCTAAAGGTGAAGAATTATTCACTGGTGTTGTCCCAATTTTGGTTGAATTAGATGGTGATGTTAATGGTCACAAATTTTCTGTCTCCGGTGAAGGTGAAGGTGATGCTACTTACGGTAAATTGACCTTAAAATTGATTTGTACTACTGGTAAATTGCCAGTTCCATGGCCAACCTTAGTCACTACTTTAGGTTATGGTTTGCAATGTTTTGCTAGATACCCAGATCATATGAAACAACATGACTTTTTCAAGTCTGCCATGCCAGAAGGTTATGTTCAAGAAAGAACTATTTTTTTCAAAGATGACGGTAACTACAAGACCAGAGCTGAAGTCAAGTTTGAAGGTGATACCTTAGTTAATAGAATCGAATTAAAAGGTATTGATTTTAAAGAAGATGGTAACATTTTAGGTCACAAATTGGAATACAACTATAACTCTCACAATGTTTACATCACTGCTGACAAACAAAAGAATGGTATCAAAGCTAACTTCAAAATTAGACACAACATTGAAGATGGTGGTGTTCAATTAGCTGACCATTATCAACAAAATACTCCAATTGGTGATGGTCCAGTCTTGTTACCAGACAACCATTACTTATCCTATCAATCTGCCTTATCCAAAGATCCAAACGAAAAGAGAGATCACATGGTCTTGTTAGAATTTGTTACTGCTGCTGGTATTACCCATGGTATGGATGAATTGTACAAATAA	Young, 2018	[1]
sdAmpR	Gene	ATGAGCACTTTTAAAGTTCTGCTATGTGGCGCGGTATTATCCCGTATTGACGCCGGGCAAGAGCAACTCGGTCGCCGCATACACTATTCTCAGAATGACTTGGTTGAGTACTCACCAGTCACAGAAAAGCATCTTACGGATGGCATGACAGTAAGAGAATTATGCAGTGCTGCCATAACCATGAGTGATAACACTGCGGCCAACTTACTTCTGACAACGATCGGAGGACCGAAGGAGCTAACCGCTTTTTTGCACAACATGGGGGATCATGTAACTCGCCTTGATCGTTGGGAACCGGAGCTGAATGAAGCCATACCAAACGACGAGCGTGACACCACGATGCCTGTAGCAATGGCAACAACGTTGCGCAAACTATTAACTGGCGAACTACTTACTCTAGCTTCCCGGCAACAATTAATAGACTGGATGGAGGCGGATAAAGTTGCAGGACCACTTCTGCGCTCGGCCCTTCCGGCTGGCTGGTTTATTGCTGATAAATCTGGAGCCGGTGAGCGTGGCTCTCGCGGTATCATTGCAGCACTGGGGCCAGATGGTAAGCCCTCCCGTATCGTAGTTATCTACACGACGGGGAGCCAGGCAACTATGGATGAACGAAATAGACAGATCGCTGAGATAGGTGCCTCACTGATTAAGCATTGGTAA	Young, 2018	[1]

sdCmR_cassette	Gene	CACGTAAGAGGTTCCAACTTTCACCATAATGAAATAAGATCACTACCGGGCGTATT<br>TTTTGAGTTATCGAGATTTTCAGGAGCTAAGGAAGCTAAAATGGAGAAAAAAATCA<br>CTGGATATACCACCGTTGATATATCCCAATGGCATCGTAAAGAACATTTTGAGGCA<br>TTTCAGTCAGTTGCTCAATGTACCTATAACCAGACCGTTCAGCTGGATATTACGGC<br>CTTTTTAAAGACCGTAAAGAAAAATAAGCACAAGTTTTATCCGGCCTTTATTCACAT<br>TCTTGCCCGCCTGATGAATGCTCATCCGGAATTTCGTATGGCAATGAAAGACGGTG<br>AGCTGGTGATATGGGATAGTGTTCACCCTTGTTACACCGTTTTCCATGAGCAAACT<br>GAAACGTTTTCATCGCTCTGGAGTGAATACCACGACGATTTCCGGCAGTTTCTACA<br>CATATATTCGCAAGATGTGGCGTGTTACGGTGAAAACCTGGCCTATTTCCCTAAAG<br>GGTTTATTGAGAATATGTTTTTCGTCTCAGCCAATCCCTGGGTGAGTTTCACCAGTT<br>TTGATTTAAACGTGGCCAATATGGACAACTTCTTCGCCCCCGTTTTCACCATGGGC<br>AAATATTATACGCAAGGCGACAAGGTGCTGATGCCGCTGGCGATTCAGGTTCATCA<br>TGCCGTTTGTGATGGCTTCCATGTCGGCAGAATGCTTAATGAATTACAACAGTACT<br>GCGATGAGTGGCAGGGCGGGGCGTAA	Young, 2018	[1]
Cas9-SV40NLS	Gene	ATGGACAAGAAGTACTCCATTGGGCTCGATATCGGCACAAACAGCGTCGGTTGGG<br>CCGTCATTACGGACGAGTACAAGGTGCCGAGCAAAAAATTCAAAGTTCTGGGCAAT<br>ACCGATCGCCACAGCATAAAGAAGAACCTCATTGGCGCCCTCCTGTTCGACTCCG<br>GGGAGACGGCCGAAGCCACGCGGCTCAAAAGAACAGCACGGCGCAGATATACCC<br>GCAGAAAGAATCGGATCTGCTACCTGCAGGAGATCTTTAGTAATGAGATGGCTAAG<br>GTGGATGACTCTTTCTTCCATAGGCTGGAGGAGTCCTTTTTGGTGGAGGAGGATAA<br>AAAGCACGAGCGCCACCCAATCTTTGGCAATATCGTGGACGAGGTGGCGTACCAT<br>GAAAAGTACCCAACCATATATCATCTGAGGAAGAAGCTTGTAGACAGTACTGATAA<br>GGCTGACTTGCGGTTGATCTATCTCGCGCTGGCGCATATGATCAAATTTCGGGGA<br>CACTTCCTCATCGAGGGGGACCTGAACCCAGACAACAGCGATGTCGACAAACTCT<br>TTATCCAACTGGTTCAGACTTACAATCAGCTTTTCGAAGAGAACCCGATCAACGCA<br>TCCGGAGTTGACGCCAAAGCAATCCTGAGCGCTAGGCTGTCCAAATCCCGGCGGC<br>TCGAAAACCTCATCGCACAGCTCCCTGGGGAGAAGAAGAACGGCCTGTTTGGTAA<br>TCTTATCGCCCTGTCACTCGGGCTGACCCCCAACTTTAAATCTAACTTCGACCTGG<br>CCGAAGATGCCAAGCTTCAACTGAGCAAAGACACCTACGATGATGATCTCGACAAT<br>CTGCTGGCCCAGATCGGCGACCAGTACGCAGACCTTTTTTTGGCGGCAAAGAACC<br>TGTCAGACGCCATTCTGCTGAGTGATATTCTGCGAGTGAACACGGAGATCACCAAA<br>GCTCCGCTGAGCGCTAGTATGATCAAGCGCTATGATGAGCACCACCAAGACTTGA<br>CTTTGCTGAAGGCCCTTGTCAGACAGCAACTGCCTGAGAAGTACAAGGAAATTTTC<br>TTCGATCAGTCTAAAAATGGCTACGCCGGATACATTGACGGCGGAGCAAGCCAGG<br>AGGAATTTTACAAATTTATTAAGCCCATCTTGGAAAAAATGGACGGCACCGAGGAG<br>CTGCTGGTAAAGCTTAACAGAGAAGATCTGTTGCGCAAACAGCGCACTTTCGACAA<br>TGGAAGCATCCCCCACCAGATTCACCTGGGCGAACTGCACGCTATCCTCAGGCGG<br>CAAGAGGATTTCTACCCCTTTTTGAAAGATAACAGGGAAAAGATTGAGAAAATCCT<br>CACATTTCGGATACCCTACTATGTAGGCCCCCTCGCCCGGGGAAATTCCAGATTC<br>GCGTGGATGACTCGCAAATCAGAAGAGACCATCACTCCCTGGAACTTCGAGGAAG<br>TCGTGGATAAGGGGGCCTCTGCCCAGTCCTTCATCGAAAGGATGACTAACTTTGAT<br>AAAAATCTGCCTAACGAAAAGGTGCTTCCTAAACACTCTCTGCTGTACGAGTACTT<br>CACAGTTTATAACGAGCTCACCAAGGTCAAATACGTCACAGAAGGGATGAGAAAGC<br>CAGCATTCCTGTCTGGAGAGCAGAAGAAAGCTATCGTGGACCTCCTCTTCAAGAC<br>GAACCGGAAAGTTACCGTGAAACAGCTCAAAGAAGACTATTTCAAAAAGATTGAAT<br>GTTTCGACTCTGTTGAAATCAGCGGAGTGGAGGATCGCTTCAACGCATCCCTGGG<br>AACGTATCACGATCTCCTGAAAATCATTAAAGACAAGGACTTCCTGGACAATGAGG<br>AGAACGAGGACATTCTTGAGGACATTGTCCTCACCCTTACGTTGTTTGAAGATAGG<br>GAGATGATTGAAGAACGCTTGAAAACTTACGCTCATCTCTTCGACGACAAAGTCAT<br>GAAACAGCTCAAGAGGCGCCGATATACAGGATGGGGGCGGCTGTCAAGAAAACTG<br>ATCAATGGGATCCGAGACAAGCAGAGTGGAAAGACAATCCTGGATTTTCTTAAGTC<br>CGATGGATTTGCCAACCGGAACTTCATGCAGTTGATCCATGATGACTCTCTCACCT<br>TTAAGGAGGACATCCAGAAAGCACAAGTTTCTGGCCAGGGGGACAGTCTTCACGA<br>GCACATCGCTAATCTTGCAGGTAGCCCAGCTATCAAAAAGGGAATACTGCAGACC	DiCarlo et al., Addgene, Plasmids #43802 & #43803	[9]

LbCpf1-SV40-NLS	Gene	ATGTCTAAGTTGGAAAAATTCACCAACTGTTACTCTTTGTCTAAGACTTTGAGATTC<br>AAGGCCATCCCAGTTGGTAAGACCCAAGAAAACATCGACAACAAGAGACTATTAGT<br>TGAAGATGAAAAGAGAGCTGAAGACTACAAGGGTGTCAAGAAATTGTTGGACAGAT<br>ACTACTTGTCTTTTATCAACGACGTTTTGCATTCCATCAAGCTAAAGAACTTGAATA<br>ACTACATCTCTTTGTTCAGAAAGAAGACTAGAACTGAAAAGGAAAATAAGGAATTG<br>GAAAACTTGGAAATCAACTTGAGAAAGGAAATTGCTAAGGCTTTCAAGGGTAATGA<br>AGGTTACAAGTCTTTATTCAAGAAAGACATCATTGAAACCATTTTGCCAGAATTTTT<br>GGATGATAAGGATGAAATTGCTTTGGTTAACTCTTTCAACGGTTTCACCACTGCTTT<br>CACTGGTTTCTTCGACAACAGAGAAAACATGTTCTCCGAGGAAGCTAAATCCACTT<br>CTATTGCTTTCAGATGTATCAACGAAAACTTGACCCGTTACATCTCTAACATGGACA<br>TTTTTGAAAAGGTCGACGCCATCTTTGACAAGCACGAAGTCCAAGAAATCAAGGAA<br>AAGATCTTAAACTCCGACTACGATGTCGAAGATTTCTTCGAAGGTGAATTCTTCAAC<br>TTTGTTTTAACCCAAGAAGGTATCGATGTCTACAACGCCATTATCGGTGGTTTTGTC<br>ACTGAATCTGGTGAAAAGATCAAGGGTTTGAACGAATACATTAACTTGTACAACCA<br>AAAGACCAAACAAAAATTGCCAAAGTTCAAGCCATTGTACAAGCAAGTTTTGTCTGA<br>CAGAGAATCTTTGTCTTTTTACGGTGAAGGGTACACCTCTGACGAAGAAGTCTTGG<br>AAGTCTTCAGAAACACTTTGAACAAGAACTCTGAAATCTTCTCCTCCATCAAGAAGT<br>TAGAAAAGTTGTTCAAGAACTTCGATGAATACTCTTCTGCTGGTATCTTCGTTAAGA<br>ACGGTCCAGCCATCTCTACCATTTCTAAGGATATCTTTGGTGAATGGAACGTCATT<br>AGAGACAAATGGAACGCTGAATACGATGACATCCATTTGAAGAAAAAGGCTGTTGT<br>CACCGAAAAGTACGAAGACGACAGAAGAAAATCCTTCAAGAAGATCGGTTCCTTCT<br>CCTTGGAACAATTACAAGAATACGCCGATGCCGATTTGTCCGTTGTCGAAAAATTG<br>AAGGAAATTATTATTCAAAAGGTTGATGAAATTTACAAAGTTTACGGTTCCTCTGAA<br>AAGTTATTCGATGCTGATTTCGTCTTGGAAAAGTCTTTGAAGAAGAACGACGCTGT<br>TGTCGCTATCATGAAGGACTTGTTGGACTCTGTCAAATCTTTCGAAAACTATATCAA<br>GGCCTTCTTCGGTGAAGGTAAGGAAACTAACAGAGATGAATCCTTCTACGGTGACT<br>TTGTCTTGGCTTACGATATTTTGTTGAAGGTTGACCACATCTACGATGCCATCAGAA<br>ACTACGTTACTCAAAAGCCATACTCTAAGGACAAATTCAAGTTGTACTTCCAAAACC<br>CACAATTCATGGGTGGTTGGGATAAGGACAAGGAAACTGACTACAGAGCTACCATT<br>TTGAGATACGGTTCCAAGTACTACTTGGCCATCATGGACAAGAAGTACGCCAAGTG<br>TTTGCAAAAGATTGACAAGGACGATGTCAACGGTAACTACGAAAAGATTAACTACA<br>AGTTGTTGCCAGGTCCAAACAAGATGTTGCCAAAGGTTTTCTTCTCCAAAAAGTGG<br>ATGGCTTACTACAACCCATCTGAAGACATCCAAAAGATCTACAAGAACGGTACTTT<br>CAAAAAGGGTGACATGTTCAACTTAAACGACTGTCACAAGTTGATCGACTTCTTCA<br>AGGACTCCATCTCTAGATACCCAAAATGGTCCAACGCTTACGATTTCAACTTCTCT<br>GAAACTGAAAAATACAAGGATATTGCTGGTTTCTACCGTGAAGTCGAGGAACAAGG<br>TTATAAGGTTTCTTTCGAATCCGCTTCTAAGAAAGAAGTTGACAAATTAGTCGAAGA<br>AGGTAAGTTGTACATGTTCCAAATCTACAACAAAGATTTCTCCGACAAGTCTCACG<br>GTACTCCAAACTTGCACACCATGTACTTCAAGTTGCTATTCGATGAAAACAACCAC<br>GGTCAAATCAGATTGTCTGGTGGTGCTGAATTGTTCATGAGACGTGCTTCTCTAAA	Verwaal et al., Addgene, Plasmid #101748	[3]
trp1	Gene	ATGTCTGTTATTAATTTCACAGGTAGTTCTGGTCCATTGGTGAAAGTTTGCGGCTTG<br>CAGAGCACAGAGGCCGCAGAATGTGCTCTAGATTCCGATGCTGACTTGCTGGGTA<br>TTATATGTGTGCCCAATAGAAAGAGAACAATTGACCCGGTTATTGCAAGGAAAATTT<br>CAAGTCTTGTAAAAGCATATAAAAATAGTTCAGGCACTCCGAAATACTTGGTTGGC<br>GTGTTTCGTAATCAACCTAAGGAGGATGTTTTGGCTCTGGTCAATGATTACGGCAT<br>TGATATCGTCCAACTGCATGGAGATGAGTCGTGGCAAGAATACCAAGAGTTCCTCG<br>GTTTGCCAGTTATTAAAAGACTCGTATTTCCAAAAGACTGCAACATACTACTCAGTG<br>CAGCTTCACAGAAACCTCATTCGTTTATTCCCTTGTTTGATTCAGAAGCAGGTGGG<br>ACAGGTGAACTTTTGGATTGGAACTCGATTTCTGACTGGGTTGGAAGGCAAGAGA<br>GCCCCGAAAGCTTACATTTTATGTTAGCTGGTGGACTGACGCCAGAAAATGTTGGT<br>GATGCGCTTAGATTAAATGGCGTTATTGGTGTTGATGTAAGCGGAGGTGTGGAGA<br>CAAATGGTGTAAAAGACTCTAACAAAATAGCAAATTTCGTCAAAAATGCTAAGAAAT	Verwaal et al., Addgene, Plasmid #101748	[3]
Cre	Gene	ATGTCCAATTTACTGACCGTACACCAAAATTTGCCTGCATTACCGGTCGATGCAAC<br>GAGTGATGAGGTTCGCAAGAACCTGATGGACATGTTCAGGGATCGCCAGGCGTTT<br>TCTGAGCATACCTGGAAAATGCTTCTGTCCGTTTGCCGGTCGTGGGCGGCATGGT<br>GCAAGTTGAATAACCGGAAATGGTTTCCCGCAGAACCTGAAGATGTTCGCGATTAT<br>CTTCTATATCTTCAGGCGCGCGGTCTGGCAGTAAAAACTATCCAGCAACATTTGGG<br>CCAGCTAAACATGCTTCATCGTCGGTCCGGGCTGCCACGACCAAGTGACAGCAAT<br>GCTGTTTCACTGGTTATGCGGCGGATCCGAAAAGAAAACGTTGATGCCGGTGAAC<br>GTGCAAAACAGGCTCTAGCGTTCGAACGCACTGATTTCGACCAGGTTCGTTCACTC<br>ATGGAAAATAGCGATCGCTGCCAGGATATACGTAATCTGGCATTTCTGGGGATTGC<br>TTATAACACCCTGTTACGTATAGCCGAAATTGCCAGGATCAGGGTTAAAGATATCT<br>CACGTACTGACGGTGGGAGAATGTTAATCCATATTGGCAGAACGAAAACGCTGGTT<br>AGCACCGCAGGTGTAGAGAAGGCACTTAGCCTGGGGGTAACTAAACTGGTCGAGC<br>GATGGATTTCCGTCTCTGGTGTAGCTGATGATCCGAATAACTACCTGTTTTGCCGG<br>GTCAGAAAAAATGGTGTTGCCGCGCCATCTGCCACCAGCCAGCTATCAACTCGCG<br>CCCTGGAAGGGATTTTTGAAGCAACTCATCGATTGATTTACGGCGCTAAGGATGAC<br>TCTGGTCAGAGATACCTGGCCTGGTCTGGACACAGTGCCCGTGTCGGAGCCGCG<br>CGAGATATGGCCCGCGCTGGAGTTTCAATACCGGAGATCATGCAAGCTGGTGGCT<br>GGACCAATGTAAATATTGTCATGAACTATATCCGTACCCTGGATAGTGAAACAGGG<br>GCAATGGTGCGCCTGCTGGAAGATGGCGATTAG	Hegemann et al., Euroscarf, P30672	[4]

ccdB	Gene	ATGCAGTTTAAGGTTTACACCTATAAAAGAGAGAGCCGTTATCGTCTGTTTGTGGA<br>TGTACAGAGTGATATTATTGACACGCCCGGGCGACGGATGGTGATCCCCCTGGCC<br>AGTGCACGTCTGCTGTCAGATAAAGTCTCCCGTGAACTTTACCCGGTGGTGCATAT<br>CGGGGATGAAAGCTGGCGCATGATGACCACCGATATGGCCAGTGTGCCGGTCTC<br>CGTTATCGGGGAAGAAGTGGCTGATCTCAGCCACCGCGAAAATGACATCAAAAAC<br>GCCATTAACCTGATGTTCTGGGGAATATAA	Berman 2018	[10]
ylCas9-NLS	Gene	ATGGATAAGAAATACTCCATTGGCCTGGACATCGGAACCAACTCCGTGGGTTGGG<br>CCGTGATCACCGATGAGTACAAGGTGCCCTCTAAGAAATTCAAGGTCCTGGGCAA<br>CACCGACCGACACTCCATCAAGAAGAACCTGATCGGCGCTCTGCTCTTCGACTCT<br>GGCGAGACCGCTGAGGCCACCCGACTGAAGCGAACCGCTCGAAGACGATACACC<br>CGAAGAAAGAACCGAATCTGTTACCTGCAGGAGATCTTCTCTAACGAGATGGCCAA<br>GGTGGACGACTCTTTCTTCCACCGACTGGAGGAGTCTTTCCTGGTGGAGGAGGAC<br>AAGAAGCACGAGCGACACCCCATCTTCGGCAACATCGTGGACGAGGTGGCCTACC<br>ACGAGAAGTACCCCACCATCTACCACCTGCGAAAGAAGCTGGTGGACTCTACCGA<br>CAAGGCCGACCTGCGACTGATCTACCTGGCCCTGGCCCACATGATCAAGTTCCGA<br>GGCCACTTCCTGATCGAGGGCGACCTGAACCCCGACAACTCTGACGTGGACAAGC<br>TGTTCATCCAGCTGGTGCAGACCTACAACCAGCTCTTCGAAGAGAACCCCATTAAC<br>GCTTCTGGCGTGGATGCTAAGGCCATCCTGTCTGCCCGACTGTCTAAGTCTCGAC<br>GACTCGAGAACCTGATTGCTCAGCTCCCCGGAGAGAAGAAGAACGGTCTGTTCGG<br>AAACCTGATTGCTCTGTCCCTGGGTCTCACCCCTAACTTCAAGTCCAACTTCGATC<br>TGGCTGAGGACGCTAAGCTGCAGCTGTCTAAGGACACCTACGACGATGACCTGGA<br>TAACCTGCTCGCCCAGATTGGCGACCAGTACGCCGACCTGTTCCTGGCCGCCAAG<br>AACCTGTCTGACGCCATCCTGCTGTCTGACATCCTGCGAGTGAACACCGAGATCA<br>CCAAGGCCCCCCTGTCTGCCTCCATGATTAAGCGATACGATGAGCACCACCAGGA<br>TCTGACCCTCCTCAAGGCTCTGGTCCGACAGCAGCTGCCCGAGAAGTACAAGGAG<br>ATTTTCTTCGACCAGTCTAAGAACGGCTACGCCGGCTACATCGACGGCGGCGCCT<br>CTCAGGAGGAGTTCTACAAGTTCATTAAGCCCATCCTGGAGAAGATGGACGGAAC<br>CGAGGAACTGCTCGTGAAGCTGAACCGAGAGGACCTCCTGCGAAAGCAGCGAAC<br>CTTCGACAACGGCTCTATCCCCCACCAGATCCACCTGGGCGAGCTGCACGCCATC<br>CTGCGACGACAGGAGGACTTCTACCCCTTCCTGAAGGACAACCGAGAGAAGATCG<br>AGAAGATCCTGACCTTCCGAATCCCCTACTACGTGGGACCCCTGGCCCGAGGAAA<br>CTCTCGATTCGCTTGGATGACCCGAAAGTCTGAGGAGACCATTACCCCCTGGAAC<br>TTCGAGGAGGTGGTGGATAAGGGCGCCTCTGCTCAGTCTTTCATCGAGCGAATGA<br>CCAACTTCGACAAGAACCTCCCCAACGAGAAGGTCCTGCCCAAGCACTCTCTGCT<br>CTACGAGTACTTCACCGTCTACAACGAGCTCACCAAGGTCAAGTACGTGACCGAG<br>GGAATGCGAAAGCCCGCTTTCCTGTCTGGAGAGCAGAAGAAGGCTATTGTGGATC<br>TGCTCTTCAAGACTAACCGAAAGGTCACCGTCAAGCAGCTGAAGGAGGATTACTTC<br>AAGAAGATTGAGTGTTTCGATTCTGTCGAGATCTCCGGCGTCGAGGACCGATTCAA<br>CGCCTCTCTGGGTACCTACCACGACCTGCTGAAGATTATCAAGGACAAGGATTTCC<br>TGGATAACGAGGAGAACGAGGATATTCTCGAGGACATTGTCCTGACCCTCACCCT<br>GTTCGAGGATCGAGAGATGATTGAGGAGCGACTCAAGACCTACGCTCACCTGTTC<br>GACGACAAGGTGATGAAGCAGCTGAAGCGACGACGATACACCGGCTGGGGCCGA<br>CTGTCTCGAAAGCTGATCAACGGCATCCGAGACAAGCAGTCTGGCAAGACCATCC<br>TGGACTTCCTGAAGTCTGACGGCTTCGCCAACCGAAACTTCATGCAGCTGATCCAC<br>GACGACTCTCTGACCTTCAAGGAGGACATCCAGAAGGCCCAGGTGTCTGGCCAGG<br>GCGACTCTCTGCACGAGCACATCGCCAACCTGGCCGGCTCTCCCGCCATTAAGAA	Schwartz et al., Addgene, Plasmid # 70007	[5]
YlLeu2	Gene	ATGGAACCCGAAACTAAGAAGACCAAGACTGACTCCAAGAAGATTGTTCTTCTCGG<br>CGGCGACTTCTGTGGCCCCGAGGTGATTGCCGAGGCCGTCAAGGTGCTCAAGTCT<br>GTTGCTGAGGCCTCCGGCACCGAGTTTGTGTTTGAGGACCGACTCATTGGAGGAG<br>CTGCCATTGAGAAGGAGGGCGAGCCCATCACCGACGCTACTCTCGACATCTGCCG<br>AAAGGCTGACTCTATTATGCTCGGTGCTGTCGGAGGCGCTGCCAACACCGTATGG<br>ACCACTCCCGACGGACGAACCGACGTGCGACCCGAGCAGGGTCTCCTCAAGCTG<br>CGAAAGGACCTGAACCTGTACGCCAACCTGCGACCCTGCCAGCTGCTGTCGCCCA<br>AGCTCGCCGATCTCTCCCCCATCCGAAACGTTGAGGGCACCGACTTCATCATTGT<br>CCGAGAGCTCGTCGGAGGTATCTACTTTGGAGAGCGAAAGGAGGATGACGGATCT<br>GGCGTCGCTTCCGACACCGAGACCTACTCCGTTCCTGAGGTTGAGCGAATTGCCC<br>GAATGGCCGCCTTCCTGGCCCTTCAGCACAACCCCCCTCTTCCCGTGTGGTCTCT<br>TGACAAGGCCAACGTGCTGGCCTCCTCTCGACTTTGGCGAAAGACTGTCACTCGA<br>GTCCTCAAGGACGAATTCCCCCAGCTCGAGCTCAACCACCAGCTGATCGACTCGG<br>CCGCCATGATCCTCATCAAGCAGCCCTCCAAGATGAATGGTATCATCATCACCACC<br>AACATGTTTGGCGATATCATCTCCGACGAGGCCTCCGTCATCCCCGGTTCTCTGG<br>GTCTGCTGCCCTCCGCCTCTCTGGCTTCTCTGCCCGACACCAACGAGGCGTTCGG<br>TCTGTACGAGCCCTGTCACGGATCTGCCCCCGATCTCGGCAAGCAGAAGGTCAAC<br>CCCATTGCCACCATTCTGTCTGCCGCCATGATGCTCAAGTTCTCTCTTAACATGAA<br>GCCCGCCGGTGACGCTGTTGAGGCTGCCGTCAAGGAGTCCGTCGAGGCTGGTAT<br>CACTACCGCCGATATCGGAGGCTCTTCCTCCACCTCCGAGGTCGGAGACTTGTTG<br>CCAACAAGGTCAAGGAGCTGCTCAAGAAGGAGTAAGTCGTTTCTACGACGCATTG<br>ATGGAAGGAGCAAACTGACGCGCCTGCGGGTTGGTCTACCGGCAGGGTCCGCTA	Schwartz et al., Addgene, Plasmid # 70007	[5]

αMF-NoEAEA-RFP	Gene	ATGAGATTTCCTTCAATTTTTACTGCTGTTTTATTCGCAGCATCCTCCGCATTAGCT<br>GCTCCAGTCAACACTACAACAGAAGATGAAACGGCACAAATTCCGGCTGAAGCTG<br>TCATCGGTTACTCAGATTTAGAAGGGGATTTCGATGTTGCTGTTTTGCCATTTTCCA<br>ACAGCACAAATAACGGGTTATTGTTTATAAATACTACTATTGCCAGCATTGCTGCTA<br>AAGAAGAAGGGGTATCTCTCGAGAAAAGAGGTTCTGCAACTTCCGGTATGGTGTC<br>AAAGGGAGAGGAAAATAATATGGCTATTATTAAGGAGTTTATGCGTTTTAAGGTACA<br>TATGGAAGGTTCTGTCAACGGTCACGAATTCGAAATTGAAGGTGAGGGGGAGGGG<br>AGGCCATACGAGGGAACTCAGACTGCTAAGTTAAAGGTCACTAAAGGTGGTCCTTT<br>ACCTTTCGCCTGGGATATCCTGTCTCCACAGTTTATGTACGGTTCAAAGGCTTATG<br>TGAAACATCCTGCCGATATCCCAGATTATCTTAAACTTTCTTTCCCTGAGGGTTTTA<br>AGTGGGAGAGGGTAATGAACTTTGAAGACGGTGGTGTGGTCACTGTTACTCAGGA<br>CTCAAGTCTGCAGGACGGTGAGTTCATCTACAAGGTGAAGCTGAGAGGTACCAAT<br>TTTCCATCAGATGGTCCCGTGATGCAAAAAAAGACAATGGGTTGGGAAGCTTCTAG<br>TGAACGTATGTATCCCGAAGATGGAGCTTTGAAAGGTGAAATTAAGCAAAGACTAA<br>AACTTAAGGATGGTGGACATTACGATGCTGAAGTTAAGACGACCTACAAGGCCAAA<br>AAGCCAGTCCAGTTGCCTGGAGCATACAATGTTAACATCAAATTGGATATAACTTC<br>CCATAATGAAGACTATACCATCGTCGAGCAATACGAACGAGCCGAAGGGAGACAC<br>AGTACTGGTGGTATGGATGAACTTTATAAAGGATCCGGAACCGCAGGATCCTAA	Obst et al.	[6]
Ttip1	Terminator	AGGGAACCTTTTACAACAAATATTTGAAAAATTACCTCCATTATTATACCTTCTCTTT<br>ATGTAATTGTTAGTTCGAAAATTTTTTCTTCATTAATATAATCAACTTCTAAAACTTTC<br>TAAAAACGTTCTCTTTTTCGAGATTAGTGCTTCTTCCCAATCCGTAAGAAATGTTTC<br>CTTTCTTGACAATTGGCACCAGCTGGCTACTCGTTGCTCGAAAACTACTCTCTTTTA<br>TTTTTAATTTACGAACGA	Young, 2018	[1]
Tprm9	Terminator	CAGATGACGGGAGACACTAGCACACAACTTTACCAGGCAAGGTATTTGACGCTAG<br>CATGTGTCCAATTCAGTGTCATTTATGATTTTTTGTAGTAGGATATAAATATATACAG<br>CGCTCCAAATAGTGCGGTTGCCCCAAAAACACCACGGAACCTCATCTGTTCTCGTA<br>CTTTGTTGTGACAAAGTAGCTCACTGCCTTATTATCACATTTTCATTATGCAACGCT<br>TCGGAAAATACGATGTTGAAAAT	Young, 2018	[1]
Tyhi9	Terminator	ATTCTAAACGCATAGTTGTAAGGTTGATGTATATATATATATATATATGTATATATTA<br>ATTACAATAATATGCTCCCGCCCAAATTTTTCTCCTTCAATACCGCCGGAGGCGGT<br>ATTGAAGGAAATAGACGGAGAATTCCTTATCAAGAAAGCTTCCATCAAAGTGTACA<br>TAAGAAGTGCCGAAATTCGAAGTATTCTTTCAGAGAGTATTTTTGCAACATACCAAT<br>AAGCCAAATTACTC	Young, 2018	[1]
Tagtef1	Terminator	CAGTACTGACAATAAAAAGATTCTTGTTTTCAAGAACTTGTCATTTGTATAGTTTTTT<br>TATATTGTAGTTGTTCTATTTTAATCAAATGTTAGCGTGATTTATATTTTTTTTCGCCT<br>CGACATCATCTGCCCAGATGCGAAGTTAAGTGCGCAGAAAGTAATATCATGCGTCA<br>ATCGTATGTGAATGCTGGTCGCTATACTGCTGTCGATTCGATACTAACGCCGCCAT<br>CCAGTGTCGA	Young, 2018	[1]
Trpl41b	Terminator	CGGATTGAGAGCAAATCGTTAAGTTCAGGTCAAGTAAAAATTGATTTCGAAAACTAA<br>TTTCTCTTATACAATCCTTTGATTGGACCGTCATCCTTTCGAATATAAGATTTTGTTA<br>AGAATATTTTAGACAGAGATCTACTTTATATTTAATATCTAGATATTACATAATTTCC<br>TCTCTAATAAAATATCATTAATAAAATAAAAATGAAGCGATTTGATTTTGTGTTGTCA<br>ACTTAGTTTGCCGCTATGCCTCTTGGGTAATGCTATTATTGAATCGAAGGGCTTTAT<br>TATATTACCCTTTAGCTTATTCTGAGGTTTCTGTGGCGTGCAAAGTGATGAACCGG<br>GCGGGTTTTAAGGATAAAATCAAAAAGTGAAAAAATGAACGGAAAATGGAATACCT<br>GTGAAATGGAGAATGATAATGAATCTTTCTGTCGTGCTTGAAAGATTTTCGGCT	Young, 2018	[1]
Trpl15a	Terminator	CTGGTTGATGGAAAATATAATTTTATTGGGCAAACTTTTGTTTATCTGATGTGTTTTA<br>TACTATTATCTTTTTAATTAATGATTCTATATACAAACCTGTATATTTTTTCTTTAACC<br>AATTTTTTTTTTTATAGACCTAGAGCTGTACT	Young, 2018	[1]
Tadh1	Terminator	CGAATTTCTTATGATTTATGATTTTTATTATTAAATAAGTTATAAAAAAAATAAGTGTA<br>TACAAATTTTAAAGTGACACTTAGGTTTTAAAACGAAAATTC	Young, 2018	[1]
Tsup4	Terminator	TTTTTTTGTTTTTTATGTCT		[9]
Tspo1	Terminator	GAGAGTAGACTTTTTCTGTGAAATTTAATGAGTTTTTGTTCACCTTTTTTACTTTTCT<br>TCTATGCCATATGGTTAAATAAAACATGGTTCAAACGCGATTCTTTTTTATTATCTAT<br>GTCTTTATTATGTAGTCATCACTATAATAACCGTTAATGAGCGACATAGTTGCATCA<br>GTTTTTAAGGCCAATTTGATTCAGAACCTTTTCAAGTTCTTCACTT	Young, 2018	[1]
Ttdh1	Terminator	GCAATCTTGATGAGGATAATGATTTTTTTTTGAATATACATAAATACTACCGTTTTTC<br>TGCTAGATTTTGTGAAGACGTAAATAAGTACATATTACTTTTTAAGCCAAGACAAGA<br>TTAAGCATTAACTTTACCCTTTTCTCTTCTAAGTTTCAATACTAGTTATCACTGTTTA<br>AAAGTTATGGCGAGAACGTCGGCGGTTAAAATATATTACCCTGAACGTGGTGAATT<br>GAAGTTCTAGGATGGTTTAAAGATTTTTCCTTTTTGGGAAATAAGTAAACAATATATT<br>GCTGCCTT	Young, 2018	[1]
Tecm10	Terminator	ACAAGATAATAAAAAAGATAATATTTTCGTTTAAAATTTCAGAAATATTGCTTACATC<br>AAACGAAATAGTAAGCGTAAACCATATATCCTTTCAACGATATGTATCATTTTTATA<br>GTCTTTTGGCGGTATAAAAAGAATAGCCAAAGGTGGAAGTCAGGTTAAACCAAAAG<br>AAACTACCCGCAAGGGATGTATGCATTTGAACATAAAAAACT	Young, 2018	[1]
Ttdh3	Terminator	GTGAATTTACTTTAAATCTTGCATTTAAATAAATTTTCTTTTTATAGCTTTATGACTTA<br>GTTTCAATTTATATACTATTTTAATGACATTTTCGATTCATTGATTGAAAGCTTTGTG<br>TTTTTTCTTGATGCGCTATTGCATTGTTCTTGTCTTTTTCGCCACATGTAATATCTGT<br>AGTAGATACCTGATACATTGTGGATGCTGAGTGAAATTTTAGTTAATAATGGAGGC<br>GCTCTTAATAATTTTGGGGATATTGGCTTTTTTTTTTAAAGTTTACAAATGAATTTTT	Young, 2018	[1]
Teno1	Terminator	AGCTTTTGATTAAGCCTTCTAGTCCAAAAAACACGTTTTTTTGTCATTTATTTCATTT<br>TCTTAGAATAGTTTAGTTTATTCATTTTATAGTCACGAATGTTTTATGATTCTATATA<br>GGGTTGCAAACAAGCATTTTTCATTTTATGTTAAAACAATTTCAGGTTTACCTTTTAT<br>TCTGCTTGTGGTGACGCGTGTATCCGCCCGCTCTTTTGGTCACCCATGTATTTAAT<br>TGCATAAATAATTCTTAAAAGTGGAGCTAGTCTATTTCTATTTACATACCTCTCATTT	Young, 2018	[1]

Trps9a	Terminator	TTGTGCTGATTTTTATTTCTTAATTACGTGTTGAAATCACGATACTGGCATCCGATAGGGGAGTTGTATTCACAATACTATCGATTTTGTAAAATAGGACGAAGAACTTTTTATATACGAGCATTTCCTAATTAGTAGGAAGCGGAAAATAATAATATAAGAAAGTAAACGCAAAAGATAGGCTGACTGCCTTCATTCGACTAGGAGGTGAGGCGACATATTTGTCACCATTCAAGTTACCGAGATGGTAGAGAGGTGGATGGCTCGGGTGAGCTTGATTGTACACTGCAGCAACGATGC	Young, 2018	[1]
Teno2	Terminator	ATCCTAACTCGAGAGTGCTTTTAACTAAGAATTATTAGTCTTTTCTGCTTATTTTTTCATCATAGTTTAGAACACTTTATATTAACGAATAGTTTATGAATCTATTTAGGTTTAAAAATTGATACAGTTTTATAAGTTACTTTTTCAAAGACTCGTGCTGTCTATTGCATAATGCACTGGAAGGGGAAAAAAAAGGTGCACACGCGTGGCTTTTTCTTGAATTTGCAGTTTGAAAAATGCTG	Lee, 2015	[2]
Tyol036w	Terminator	GGAAAAAGGTCTTGGCTATATATAACGGCAGACAAAATATAAGTATACACGTATATATGGTGGTAAACACGCATATACTGTATGCCATGTATTTACCATTACATAGTTATTTACGCACTCTATAAAAAGTTAACATTGCATTTTAATAAATTCCTTAAATTACTCTAATTAGGATGGTAGCCCTACCTTT	Young, 2018	[1]
Tcyc1	Terminator	TCATGTAATTAGTTATGTCACGCTTACATTCACGCCCTCCCCCCACATCCGCTCTAACCGAAAAGGAAGGAGTTAGACAACCTGAAGTCTAGGTCCCTATTTATTTTTTTATAGTTATGTTAGTATTAAGAACGTTATTTATATTTCAAATTTTTCTTTTTTTTCTGTACAGACGCGTGTACGCATGTAACATTATACTGAAAACCTTGCTTGAGAAGGTTTTGGGACGCTCGAAGGCTTTAATTTGC	DiCarlo et al., Addgene, Plasmids #43802 & #43803	[9]
Taox1	Terminator	TCAAGAGGATGTCAGAATGCCATTTGCCTGAGAGATGCAGGCTTCATTTTTGATACTTTTTTATTTGTAACCTATATAGTATAGGATTTTTTTTGTCATTTTGTTTCTTCTCGTACGAGCTTGCTCCTGATCAGCCTATCTCGCAGCTGATGAATATCTTGTGGTAGGGGTTTGGGAAAATCATTCGAGTTTGATGTTTTTCTTGGTATTTCCCACTCCTCTTCAGAGTACAGAAGATTAAGTGAGA	Obst et al.	[6]
22_2	gRNA	AAGAAGAATGACTAAACAAG	This study	
gRNAscr	gRNA	TACGTATGACGTAGCTATGT	This study	
grRNA_CAN1	gRNA	GATACGTTCTCTATGGAGGA	DiCarlo et al., Addgene, Plasmids #43802 & #43803	[9]
5' int chrXV	Genome fragment for targeted integration	GACATATTCTTACCAATCCTTTCATAAGCTAATTATGCCATCCATATAGCAAGAGAATCCGGTGGGGGCGCCATGCCTATCCGGCGGCAACATTATTACTCTGGTATACGGGCGTAACTCCATAATATGCCACCACTTACCTTTAACATGTTCATGGTAGGTACCCCACCCAGCCATAAGGAAATTTTCAAAGGCGTTGGATCAAAAAATAGGCCTTTATTTCATCGCGTGATTGAGGAGCATAACATGTTTAGTGAAGGTTTCTTTTGGAAAACTTCAGTCGCTCATTATTAGAACCAGGGAGGTCCAGGCTTTGCTGGTGGGAGAGAAAGCTTATGAAGCTGGGGTTGCAGATTTGTCGATTGGTCGCCAGTACACAGTTTTAAAAAGTCAGAGAATGTAGAGAAGTATGGATCTTTGAAACCCT	Young, 2018	[1]
3' int chrXV	Genome fragment for targeted integration	ATTTTATTTTACTTTTTTTAGAATGACCTGTTCCCGACACTATGTAAGATCTAGCTTTTAACATATTATGGAAACCTGAAATGTAAAATCTGAATTTTTGTATATGTGTTTATATTTGGGTAGTTCTTTTGAGGAAAGCATGCATAGACTTGCTGTACGAACTTTATGTGACTTGTAGTGACGCTGTTTCATGAGACTTTAGCCCTTTGAACATATTATCATATCTCAGCTTGAAATACTATAGATTTACTTTTGCAGCCATTTCTTGGTGCTCCAAGGTTGTGCGTATCTATTACTTAATTTCTGTCCTTGCCAAGTTTTGCAGCAGGGCGGTCACAAGACACCTCTGCCGTCATTCCTTAGTCCTTCGGGAACACACTTATTTATGTATTTGTATTCTACAATTCTACGGTGCACAAGGGTTGGGCACTGTTGAGCTCAGCACGCAACTATTGCTGGCATGAAGATAAGATTGATTTTTGGAAGAATAAGCTTGTGA	Young, 2018	[1]
HO-L	Genome fragment for targeted integration	AATTATCCTGGGCACGAGTGAAACAAAGCTAAAACCTTTATTTAGCATGGCCATTGAATGTAACAATTATATATATCGCAAGCACAAAAAATCAAGGAGAGAGAACTACCACTTTGTTCATGTGTACAATGTTCATTATCTCCATAAGCAAAAAAAAAAAAAATAGAAAACATATGCTATAAGGTTGATATTCTCACGAGTAAGCGGCACTTGCTACTTATTGACATTGCAGATTTTTGGCTACAGAAATAGTATATTAGAGATTATAATTGCTAATCAAATCAAAATATAAAATTAGTAAACCAAACCATTTATACCCTTCCTTAGTAGTTATGGATTGTTTTTTAATGATATTTCTGCAAACCAAAGAAAGATTGTTATCCAGATAGAATTTAGTTTTGATATTCATTTTTTTGTTGAAGATTGAACGCCATATCTGGGCCTCATAATTCAAAAGACGGTGCCATTATCGGTAGCGTTTCGCATTGTACTGGATTTCAGAAATTTCACAGTTGATGAATCGAAAAGAATGGTCTCATTGCAACACGTAAGGTTAAGATGTCCCTTTTTACCATTATAGGCAATAAATGAATCATAAAACGACCGTATACTGGTGAAATAGTAGGGAGAACGAGTACCTGTAGTAAAAAGTATAAATCATAGTTAATCGGGCAATGTCCCTCGATCAAGGAGTATTGTGTCATGTTCGAGACAAACGCCAACATTTTTGTTTCTTTTGGACAAATGTTGTTTGCATTTATGATCCGTTATATTTTGATCTAATGTAGAGTTGCACGTAGTTCTTACTGGCAAAGAAATCGATGCATACCAAAAAAGAATAAAGGTGATATTTGATCTTTACCGTTTAGTTCCAACGTAAAATTGTGCCTTTGGACTTAAAATGGCGTCGT	Voth, 2001	[11]

HO-R	Genome fragment for targeted integration	CTGGGGGAACAACTTCACAGAATGTTTTGTCATATTGTCGAAGTGGTCACAAAACA<br>AGAGAAGTTCCGCCAATTATAAAAAGGGAACCCGTATATTTCAGCTTCACGGATGA<br>TTTCCAGGGTGAGAGTACTGTATATGGGCTTACGATAGAAGGCCATAAAAATTTCT<br>TGCTTGGCAACAAAATAGAAGTGAAATCATGTCGAGGCTGCTGTGTGGGAGAACA<br>GCATAAAATATCACAAAAAAAGAATCTAAAACACTGTGTTGCTTGTCCCAGAAAGG<br>GAATCAAGTATTTTTATAAAGATTGGAGTGGTAAAAATCGAGTATGTGCTAGATGCT<br>ATGGAAGATACAAATTCAGCGGTCATCACTGTATAAATTGCAAGTATGTACCAGAA<br>GCACGTGAAGTGAAAAAGGCAAAAGACAAAGGCGAAAAATTGGGCATTACGCCCG<br>AAGGTTTGCCAGTTAAAGGACCAGAGTGTATAAAATGTGGCGGAATCTTACAGTGG<br>CCTAT	Voth, 2001	[11]
Spacer 11	Connector	TCATTTGGATTGTAATTTCATACTGGAGTAAACATCTCCAGGTGTCTAAGTTCACAC<br>AGG	This study	
Spacer 12	Connector	AGGACCAAGCGACCTGTGTCGAGGTAAATACTGTTGAAAATCGTTTCTGTAGCGAT<br>TGTC	This study	
Spacer 13	Connector	TCATTTGGATTGTAATTTCATACTGGAGTAAACATCTCCAGGTGTCTAAGTTCACAC<br>AGG	This study	
Spacer 14	Connector	GTTGTGTGCTATTTATTGCTCACAGTTAAGGAGGCCACCAAGATAAAACATAGTCT<br>GATA	This study	
Spacer 15	Connector	GTTGTGTGCTATTTATTGCTCACAGTTAAGGAGGCCACCAAGATAAAACATAGTCT<br>GATA	This study	
Spacer 16	Connector	CTTAGTCAATTACGCAGTAATACTAGAAAGAATTGAGCTTCGCAGGTACTACTAAG<br>GCAT	This study	
Spacer 17	Connector	CTTAGTCAATTACGCAGTAATACTAGAAAGAATTGAGCTTCGCAGGTACTACTAAG<br>GCAT	This study	
CEN6/ARSH4	Origin of replication	GGACGGATCGCTTGCCTGTAACTTACACGCGCCTCGTATCTTTTAATGATGGAATA<br>ATTTGGGAATTTACTCTGTGTTTATTTATTTTTATGTTTTGTATTTGGATTTTAGAAA<br>GTAAATAAAGAAGGTAGAAGAGTTACGGAATGAAGAAAAAAAAATAAACAAAGGTT<br>TAAAAAATTTCAACAAAAAGCGTACTTTACATATATATTTATTAGACAAGAAAAGCAG<br>ATTAAATAGATATACATTCGATTAACGATAAGTAAAATGTAAAATCACAGGATTTTC<br>GTGTGTGGTCATCTACACAGACAAGATGAAACAATTCGGCATTAATACCTGAGAGC<br>AGGAAGAGCAAGATAAAAGGTAGTATTTGTTGGCGATCCCCCTAGAGTCTTTTACA<br>TCTTCGGAAAACAAAAACTATTTTTTCTTTAATTTCTTTTTTTACTTTCTATTTTTAAT<br>TTATATATTTATATTAAAAAATTTAAATTATAATTATTTTTATAGCACGTGATGAAAAG	Young, 2018	[1]
2µ	Origin of replication	ACGCATTTAAGCATAAACACGCACTATGCCGTTCTTCTCATGTATATATATATACAG<br>GCAACACGCAGATATAGGTGCGACGTGAACAGTGAGCTGTATGTGCGCAGCTCGC<br>GTTGCATTTTCGGAAGCGCTCGTTTTCGGAAACGCTTTGAAGTTCCTATTCCGAAG<br>TTCCTATTCTCTAGAAAGTATAGGAACTTCAGAGCGCTTTTGAAAACCAAAAGCGCT<br>CTGATGACGCACTTTCAAAAAACCAAAAACGCACCGGACTGTAACGAGCTACTAAA<br>ATATTGCGAATACCGCTTCCACAAACATTGCTCAAAAGTATCTCTTTGCTATATATC<br>TCTGTGCTATATCCCTATATAACCTACCCATCCACCTTTCGCTCCTTGAACTTGCAT<br>CTAAACTCGACCTCTACATTTTTTATGTTTATCTCTAGTATTACTCTTTAGACAAAAA<br>AATTGTAGTAAGAACTATTCATAGAGTGAATCGAAAACAATACGAAAATGTAAACAT<br>TTCCTATACGTAGTATATAGAGACAAAATAGAAGAAACCGTTCATAATTTTCTGACC<br>AATGAAGAATCATCAACGCTATCACTTTCTGTTCACAAAGTATGCGCAATCCACATC<br>GGTATAGAATATAATCGGGGATGCCTTTATCTTGAAAAAATGCACCCGCAGCTTCG<br>CTAGTAATCAGTAAACGCGGGAAGTGGTGTCAGGCTTTTTTTATGGAAGAGAAAAT<br>AGACACCAAAGTAGCCTTCTTCTAACCTTAACGGACCTACAGTGCAAAAAGTTATC<br>AAGAGACTGCATTATAGAGCGCACAAAGGAGAAAAAAAGTAATCTAAGATGCTTTG<br>TTAGAAAAATAGCGCTCTCGGGATGCATTTTTGTAGAACAAAAAAGAAGTATAGATT<br>CTTTGTTGGTAAAATAGCGCTCTCGCGTTGCATTTCTGTTCTGTAAAAATGCAGCTC<br>AGATTCTTTGTTTGAAAAATTAGCGCTCTCGCGTTGCATTTTTGTTTTACAAAAATG<br>AAGCACAGATTCTTCGTTGGTAAAATAGCGCTTTCGCGTTGCATTTCTGTTCTGTAA<br>AAATGCAGCTCAGATTCTTTGTTTGAAAAATTAGCGCTCTCGCGTTGCATTTTTGTT<br>CTACAAAATGAAGCACAGATGCTTCGT	Young, 2018	[1]
ColE1	Origin of replication	GGCCGCGTTGCTGGCGTTTTTCCATAGGCTCCGCCCCCCTGACGAGCATCACAAA<br>AATCGACGCTCAAGTCAGAGGTGGCGAAACCCGACAGGACTATAAAGATACCAGG<br>CGTTTCCCCCTGGAAGCTCCCTCGTGCGCTCTCCTGTTCCGACCCTGCCGCTTAC<br>CGGATACCTGTCCGCCTTTCTCCCTTCGGGAAGCGTGGCGCTTTCTCATAGCTCA<br>CGCTGTAGGTATCTCAGTTCGGTGTAGGTCGTTCGCTCCAAGCTGGGCTGTGTGC<br>ACGAACCCCCCGTTCAGCCCGACCGCTGCGCCTTATCCGGTAACTATCGTCTTGA<br>GCCCAACCCGGTAAGACACGACTTATCGCCACTGGCAGCAGCCACTGGTAACAGG<br>ATTAGCAGAGCGAGGTATGTAGGCGGTGCTACAGAGTTCTTGAAGTGGTGGCCTA<br>ACTACGGCTACACTAGAAGAACAGTATTTGGTATCTGCGCTCTGCTGAAGCCAGTT<br>ACCTTCGGAAAAAGAGTTGGTAGCTCTTGATCCGGCAAACAAACCACCGCTGGTA<br>GCGGTGGTTTTTTTGTTTGCAAGCAGCAGATTACGCGCAGAAAAAAAGGATCTCAA<br>GAAGATCCTTTGATCTTTTCTACGGGGTCTGACGCTCAGTGGAACGAAAACTCACG<br>TTAAGGATTTTGGTCATGA	Young, 2018	[1]

pMB1-pUC19	Origin of replication	TTTCCATAGGCTCCGCCCCCCTGACGAGCATCACAAAAATCGACGCTCAAGTCAGAGGTGGCGAAACCCGACAGGACTATAAAGATACCAGGCGTTTCCCCCTGGAAGCTCCCTCGTGCGCTCTCCTGTTCCGACCCTGCCGCTTACCGGATACCTGTCCGCCTTTCTCCCTTCGGGAAGCGTGGCGCTTTCTCATAGCTCACGCTGTAGGTATCTCAGTTCGGTGTAGGTCGTTCGCTCCAAGCTGGGCTGTGTGCACGAACCCCCCGTTCAGCCCGACCGCTGCGCCTTATCCGGTAACTATCGTCTTGAGTCCAACCCGGTAAGACACGACTTATCGCCACTGGCAGCAGCCACTGGTAACAGGATTAGCAGAGCGAGGTATGTAGGCGGTGCTACAGAGTTCTTGAAGTGGTGGCCTAACTACGGCTACACTAGAAGAACAGTATTTGGTATCTGCGCTCTGCTGAAGCCAGTTACCTTCGGAAAAAGAGTTGGTAGCTCTTGATCCGGCAAACAAACCACCGCTGGTAGCGGTGGTTTTTTTGTTTGCAAGCAGCAGATTACGCGCAGAAAAAAAGGATCTCAA	Obst et al.	[6]
EY099	Level 0 sequencing primer	CAGACAAGCCCGTCAGG	Young, 2018	[1]
EY100	Level 1 sequencing primer	GTTATCCCCTGATTCTGTGG	Young, 2018	[1]
EY101	Level 1 sequencing primer	ATTCAGCAATTTGCCCG	Young, 2018	[1]
HR1_ampF_63	Forward primer for HR1 amplification	GGCGTTATTCCTCGTATGC	This study	
HR1_ampR_63	Reverse primer for HR1 amplification	GACAATCGCTACAGAAACGAT	This study	
HR2_ampF_66	Forward primer for HR2 amplification	AGGACCAAGCGACCTGT	This study	
HR2_ampR_66	Reverse primer for HR2 amplification	CCTGTGTGAACTTAGACACCTG	This study	
HR3_ampF_62	Forward primer for HR3 amplification	TCATTTGGATTGTAATTTCATACTGG	This study	
HR3_ampR_62	Reverse primer for HR3 amplification	TAACAGGGGGACCATGT	This study	
HR4_ampF_60	Forward primer for HR4 amplification	ACACCGAACCTAGGATTAG	This study	
HR4_ampR_60	Reverse primer for HR4 amplification	TATCAGACTATGTTTTATCTTGGTG	This study	
HR5_ampF_60	Forward primer for HR5 amplification	GTTGTGTGCTATTTATTGCTC	This study	
HR5_ampR_60	Reverse primer for HR5 amplification	ATCGTAACATCTAAGAGGCTAATT	This study	

HR6_ampF_58	Forward primer for HR6 amplification	GAACCATAGTTTAAATGAATTCC	This study	
HR6_ampR_58	Reverse primer for HR6 amplification	ATGCCTTAGTAGTACCTG	This study	
SC_gen_5F	Forward primer for 5' int chrXV	GACATATTCTTACCAATCCTTTCA	This study	
SC int 5' R S11	Reverse primer for HR1 - 5' int chrXV	GATGGACACCTGGCTACTTAACCATTCGTTGTTAGTGTGTCGCATACGAGGAATAACGCCAGGGTTTCAAAGATCCATAC	This study	
SC int 3' F S16	Forward primer for HR6 - 3' int chrXV	GAACCATAGTTTAAATGAATTCCTGACAGAGTACGCAATTAGCCTCTTAGATGTTACGATTAGAATGACCTGTTCCCGACA	This study	
SC int 3' F S13	Forward primer for HR2 - 3' int chrXV	TCATTTGGATTGTAATTTCATACTGGAGTAAACATCTCCAGGTGTCTAAGTTCACACAGGTAGAATGACCTGTTCCCGACA	This study	
SC int 3' F S14	Forward primer for HR3 - 3' int chrXV	ACACCGAACCTAGGATTAGATGTGGTCTAGCACCATATTGCGGACATGGTCCCCCTGTTATAGAATGACCTGTTCCCGACA	This study	
SC_gen_3R	Reverse primer for 5' int chrXV	GTCTTGTGACCGCCCT	This study	
HO-L F	Forward primer for HO-L	AATTATCCTGGGCACGAGT	This study	
gDNA-HO-L-R S11	Reverse primer for HR1 - HO-L	GATGGACACCTGGCTACTTAACCATTCGTTGTTAGTGTGTCGCATACGAGGAATAACGCCCGTGCCACGCCATTTTAAGT	This study	
HO-R F S13	Forward primer for HR2 - HO-L	TCATTTGGATTGTAATTTCATACTGGAGTAAACATCTCCAGGTGTCTAAGTTCACACAGGCTGGGGGAACAACTTCAC	This study	
gDNA-HO-R-F S14	Forward primer for HR3 - HO-L	ACACCGAACCTAGGATTAGATGTGGTCTAGCACCATATTGCGGACATGGTCCCCCTGTTAGCTGGGGGAACAACTTCAC	This study	
gDNA-HO-R-F S15	Forward primer for HR4 - HO-L	GTTGTGTGCTATTTATTGCTCACAGTTAAGGAGGCCACCAAGATAAAACATAGTCTGATAGCTGGGGGAACAACTTCAC	This study	
gDNA-HO-R-R	Reverse primer for HO-R	ACTGTAAGATTCCGCCACAT	This study	

Supplementary Figures

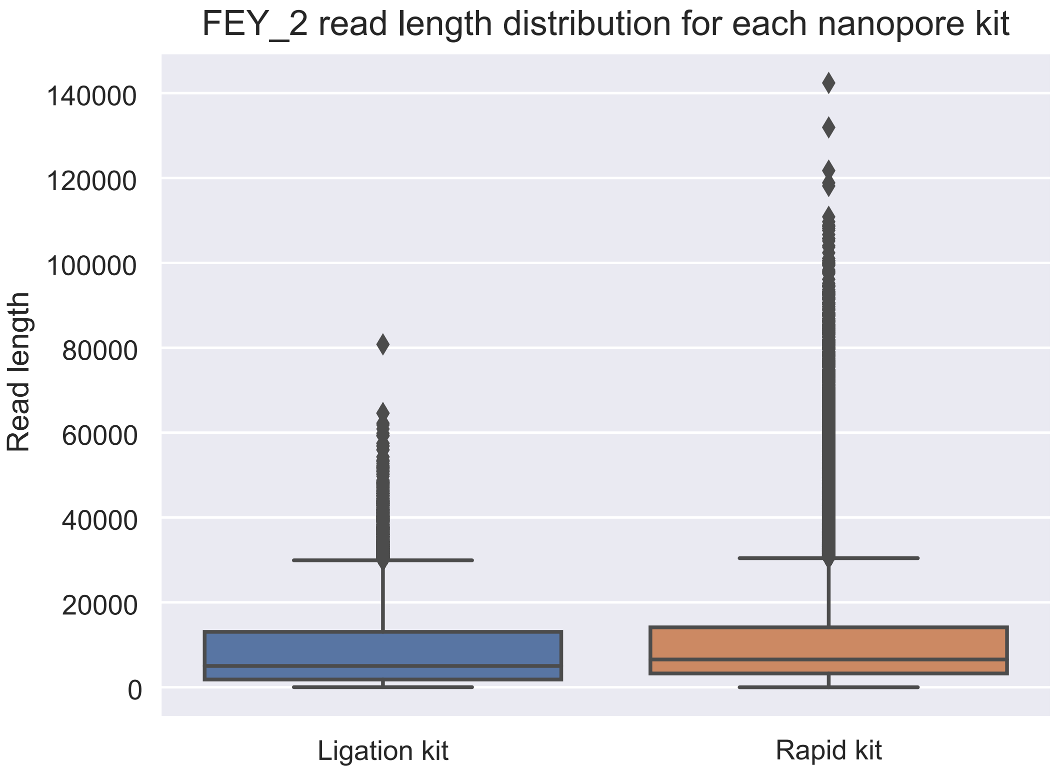

Supplementary Figure 1. Read length distribution for two Nanopore genomic DNA library preparation kits. We compared the Ligation kit (n=18,910 reads) and the Rapid kit (n=78,481 reads). The data is represented as boxplots. The middle line of the colored boxes represents the median, while the lower and upper lines of the colored boxes represent the first and third quartiles. The upper whiskers extend from the upper lines of the colored boxes to the largest value no further than $1.5 \times IQR$ from the upper lines and the lower whisker extends from the lower line to the smallest value no further than $1.5 \times IQR$ of the lower line. IQR represents the inter-quartile range. Data points beyond the whiskers are outliers that are individually plotted.

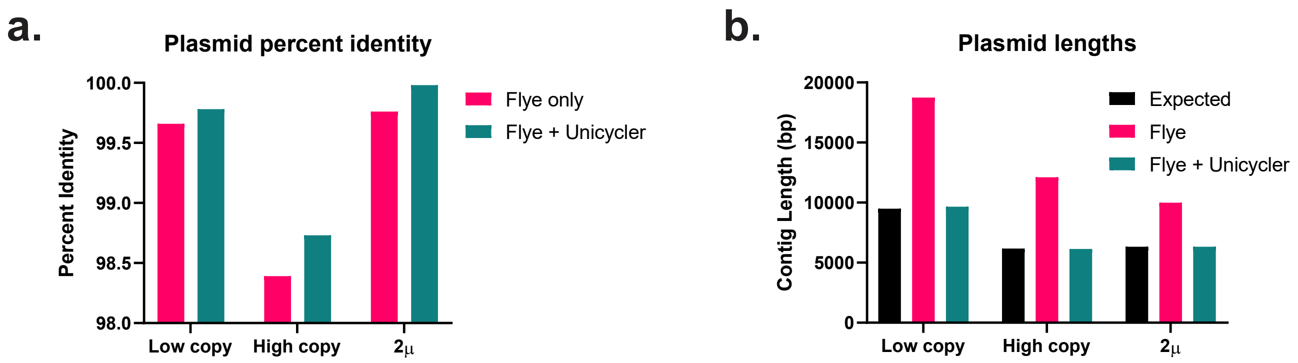

Supplementary Figure 2. Improvement to FEY_2 plasmid assembly using Unicycler. **a.** Plasmid percent identity, found using BLASTN, in the FEY_2 strain both before and after reassembly with Unicycler. **b.** Plasmid contig length in the FEY_2 strain both before after reassembly with Unicycler, compared to the expected plasmid length.

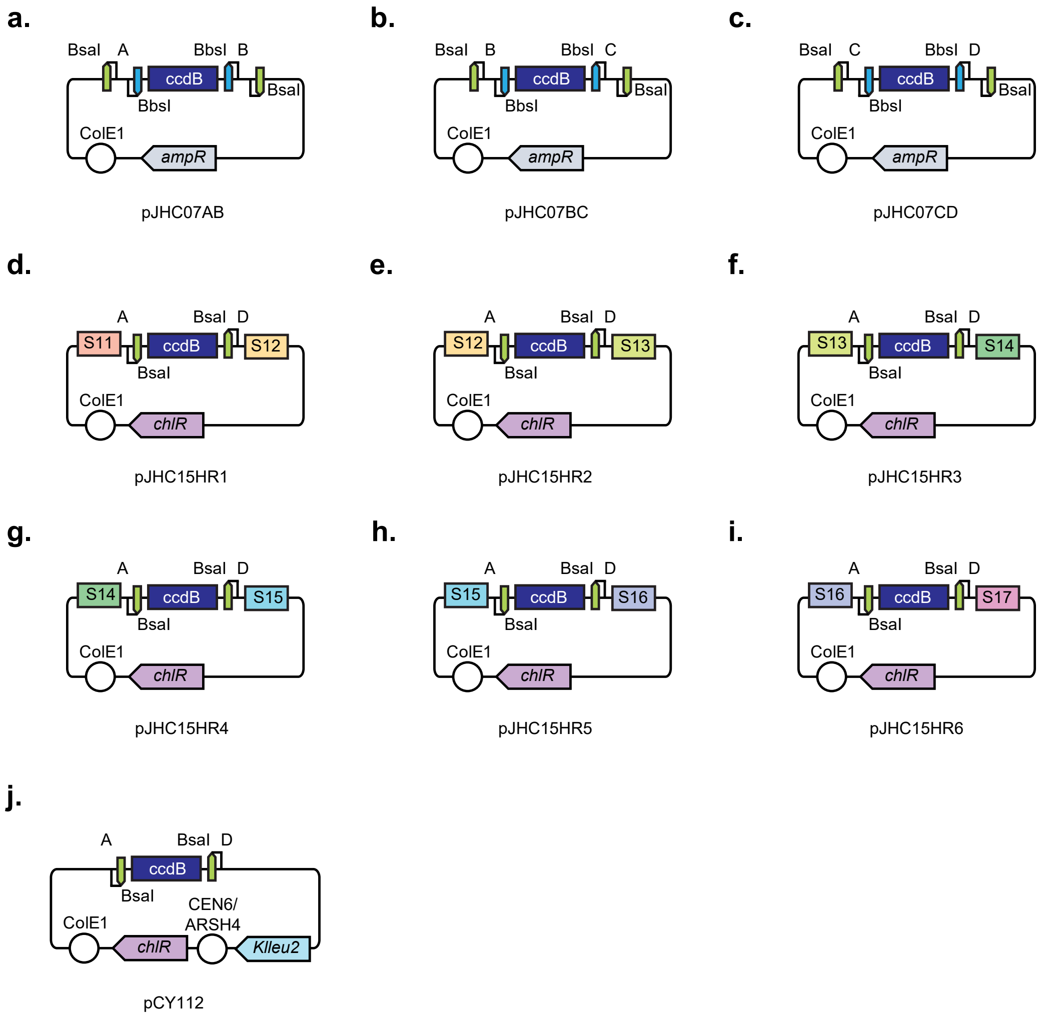

Supplementary Figure 3. Plasmid maps used for cloning in this study. **a.** The L0 cloning vector pJHC07AB designed to propagate promoter sequences. The ccdB gene is flanked by BbsI sites, 4-bp scar sites (A scar: GTGC, B scar: AATG), and BsaI sites. **b.** The L0 cloning vector pJHC07BC designed to propagate ORF sequences (B scar: AATG, C scar: TAAA). **c.** The L0 cloning vector pJHC07CD designed to propagate terminator sequences (C scar: TAAA, D scar: CCTC). **d.** The L1 cloning vector pJHC15HR1 designed for assembly of a transcription unit (promoter-ORF-terminator). The ccdB gene is flanked by BsaI sites, 4-bp scar sites (A scar: GTGC, D scar: CCTC), and 60-bp connector sequences (S11 and S12). **e.** The L1 cloning vector pJHC15HR2 with connectors S12 and S13. **f.** The L1 cloning vector pJHC15HR3 with connectors S13 and S14. **g.** The L1 cloning vector pJHC15HR4 with connectors S14 and S15. **h.** The L1 cloning vector pJHC15HR5 with connectors S15 and S16. **i.** The L1 cloning vector pJHC15HR6 with connectors S16 and S17. **j.** The low copy L1 shuttle vector pCY112. The ccdB gene is flanked by BsaI sites and 4-bp scar sites (A: GTGC, D: CCTC).

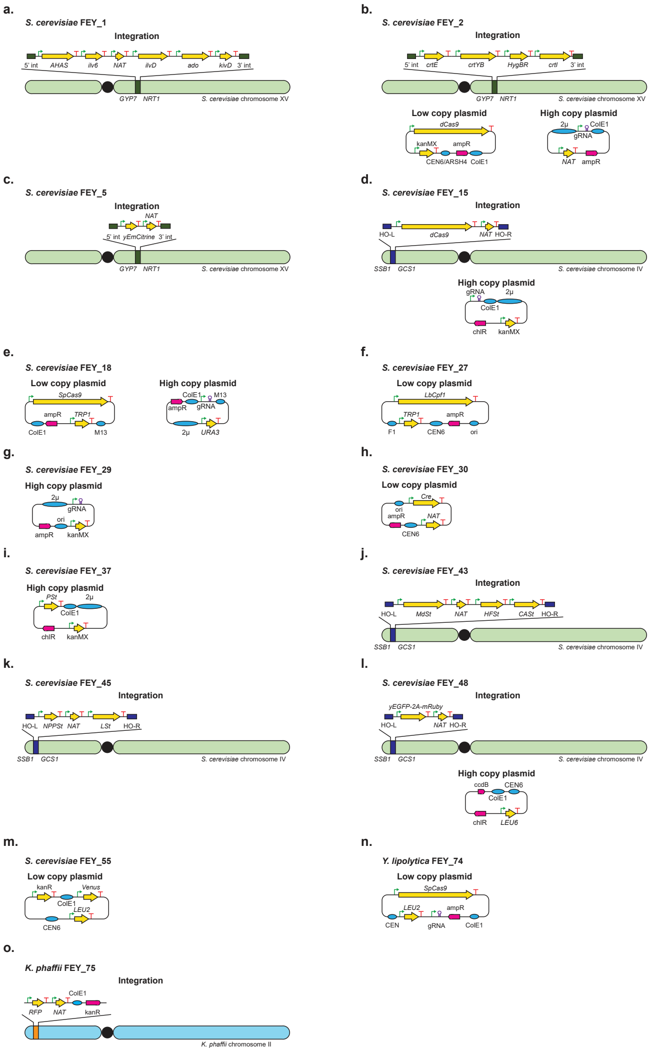

Supplementary Figure 4. Design of all engineered yeast strains. **a.** Genomic integration design for the FEY_1 strain. The six transcription unit pathway for the valine-derived compound propane was integrated into chromosome XV of the parent strain *S. cerevisiae* S288C. *b.* Genomic integration and plasmid design for the FEY_2 strain. A four transcription unit pathway for β-carotene production was integrated into chromosome XV of the parent strain *S. cerevisiae* CEN.PK113-7D. The low copy plasmid contained a dCas9 transcription unit, while the high copy plasmid expressed a gRNA. **c.** Genomic integration design for the FEY_5 strain. A two transcription unit pathway designed for expression of the yEmCitrine fluorescence protein was integrated into chromosome XV of the parent strain *S. cerevisiae* CEN.PK113-7D. **d.** Genomic integration and plasmid design for the FEY_15 strain. A two transcription unit pathway designed for expression of dCas9 was integrated into chromosome IV of the parent strain *S. cerevisiae* CEN.PK113-7D. **e.** Plasmid design for the FEY_18 strain. Two plasmids were integrated into the parent strain *S. cerevisiae* W303-α: a low copy plasmid expressing Cas9 and a high copy plasmid expressing a gRNA. **f.** Plasmid design for the FEY_27 strain. A low copy plasmid expressing LbCpf1 was transformed into the parent strain *S. cerevisiae* S288C. **g.** Plasmid design for the FEY_29 strain. A high copy plasmid expressing a gRNA was transformed into the parent strain *S. cerevisiae* S288C. **h.** Plasmid design for the FEY_30 strain. A low copy plasmid expressing the Cre recombinase was transformed into the parent strain *S. cerevisiae* S288C. **i.** Plasmid design for the FEY_37 strain. A high copy plasmid expressing a prespatane synthase was transformed into the parent strain *S. cerevisiae* S288C. **j.** Genomic integration design for the FEY_43 strain. The four transcription unit pathway designed for carnosic acid production was integrated into chromosome IV of the parent strain *S. cerevisiae* S288C. **k.** Genomic integration design for the FEY_45 strain. The three transcription unit pathway designed for limonene production was integrated into chromosome IV of the parent strain *S. cerevisiae* S288C. **l.** Genomic integration and plasmid design for the FEY_48 strain. The two transcription unit pathway designed for expression of both GFP and RFP using the self-cleaving 2A peptide was integrated into chromosome IV of the parent strain *S. cerevisiae* BY4742. The low copy yeast shuttle vector was "empty" and contained the ccdB selection gene. **m.** Plasmid design for the FEY_55 strain. A low copy plasmid expressing the Venus fluorescence protein was transformed into the parent strain *S. cerevisiae* BY4741. **n.** Plasmid design for the FEY_74 strain. A low copy plasmid expressing both Cas9 and a gRNA was transformed into the parent strain *Y. lipolytica* Po1f. **o.** Genomic integration design for the FEY_75 strain. A serine recombinase was used to integrate the two transcription unit pathway designed for expression of RFP into chromosome II of *K. phaffii* ATCC 76273. Cloning-related parts were also included as part of the integration.

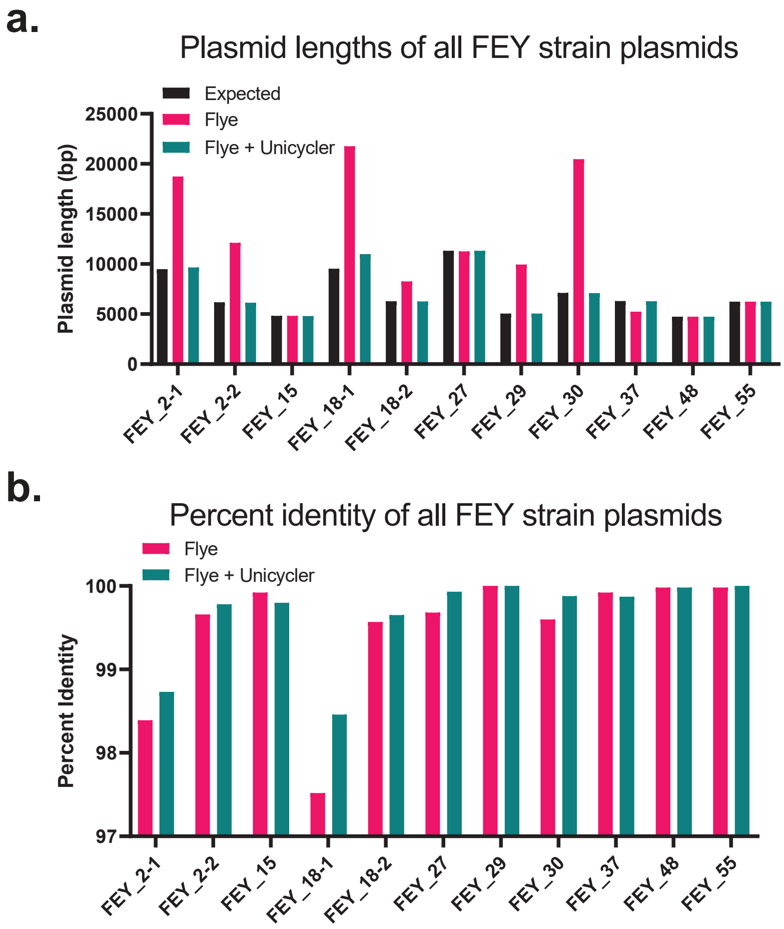

Supplementary Figure 5. Comparison of engineered strain assembly plasmids with or without Unicycler. **a.** Length of each engineered plasmid from each genome assembly strategy against the expected length. **b.** BLASTN percent identity of each engineered plasmid from each genome assembly strategy.

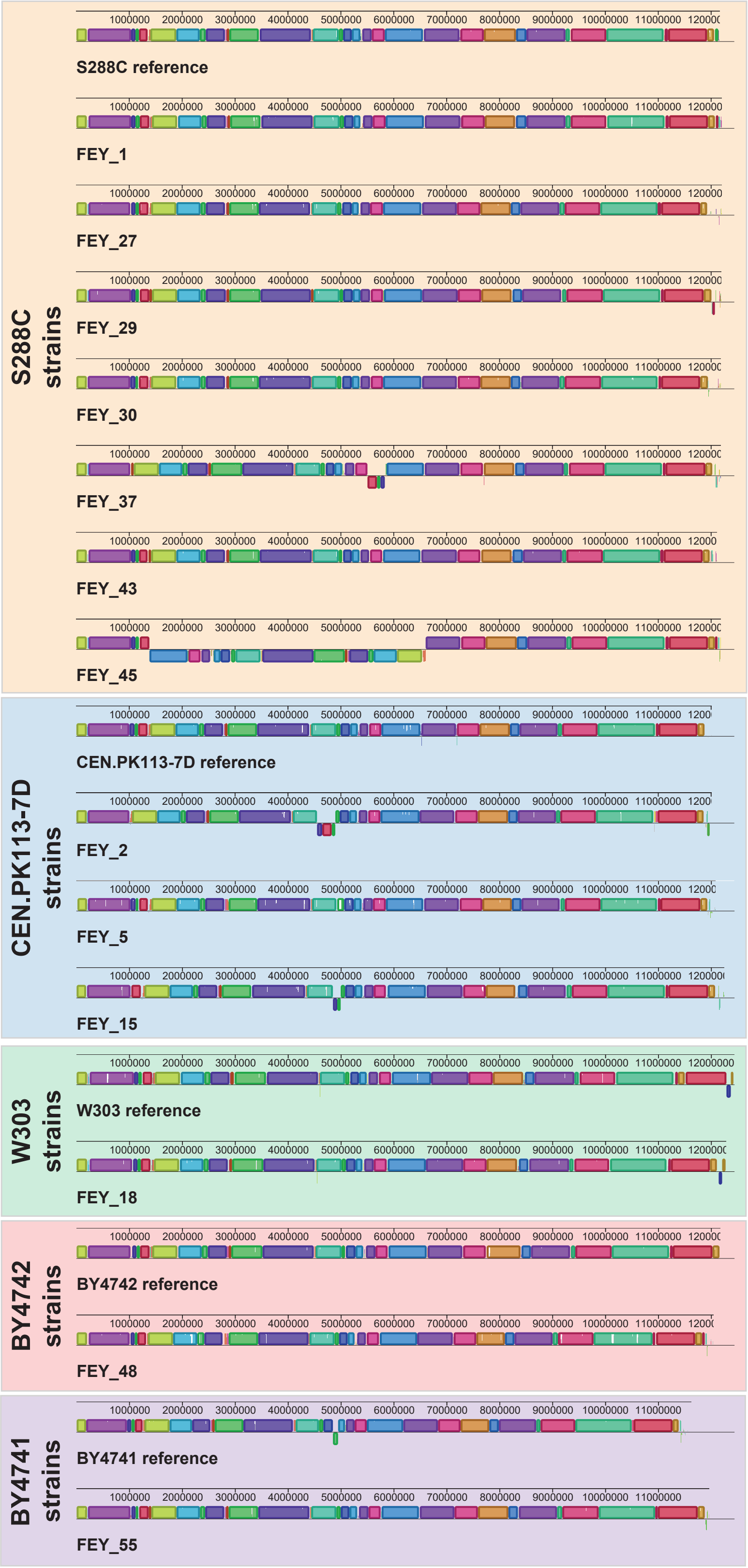

Supplementary Figure 6. Mauve whole genome alignment of all *S. cerevisiae* strains in this study. The colored blocks represent regions of the genomes that align. A block below the center line indicates the aligned sequence was in the reverse direction. The vertical red lines representing contig breaks were omitted for visual clarity.

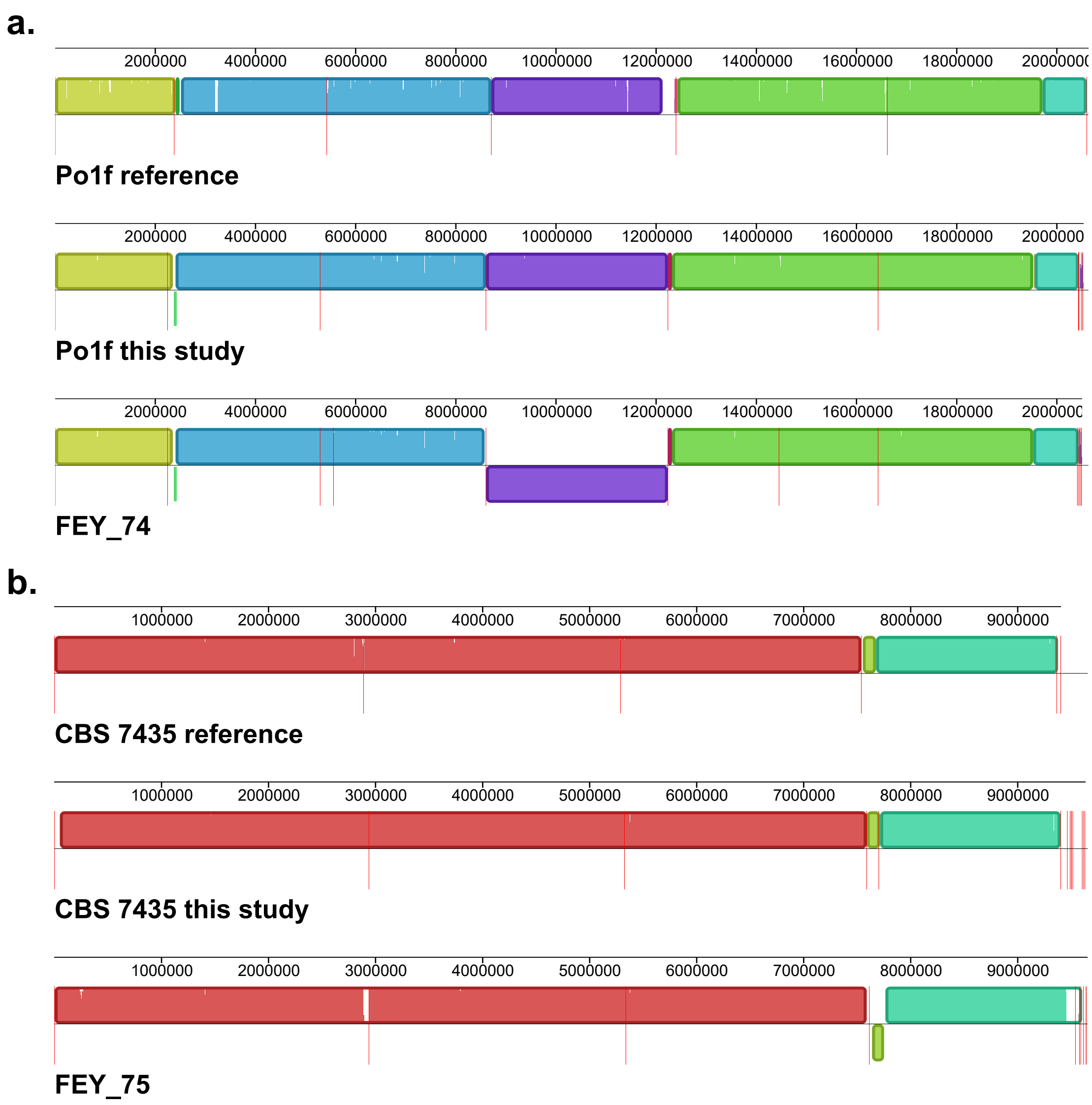

Supplementary Figure 7. Mauve whole genome alignment of all nonconventional yeasts in this study. The colored blocks represent regions of the genomes that align, while the vertical red lines indicate a new contig. A block below the center line indicates the aligned sequence was in the reverse direction. **a.** Comparison of FEY_74 against two *Y. lipolytica* PO1f reference assemblies, including one from this study. **b.** Comparison of FEY_75 against two *K. phaffii* CBS 7435 reference assemblies, including one from this study.

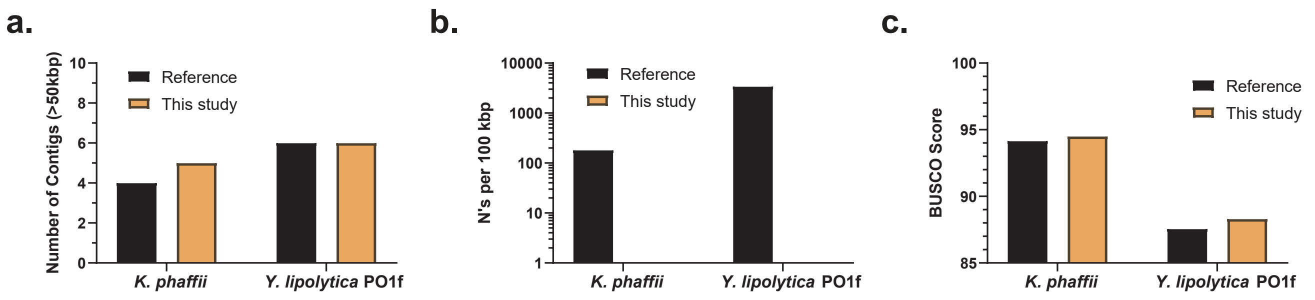

Supplementary Figure 8. Resequenced nonconventional yeast genomes compared to reference. **a.** Number of contigs.**b.** N's per 100 kbp **c.** BUSCO score.

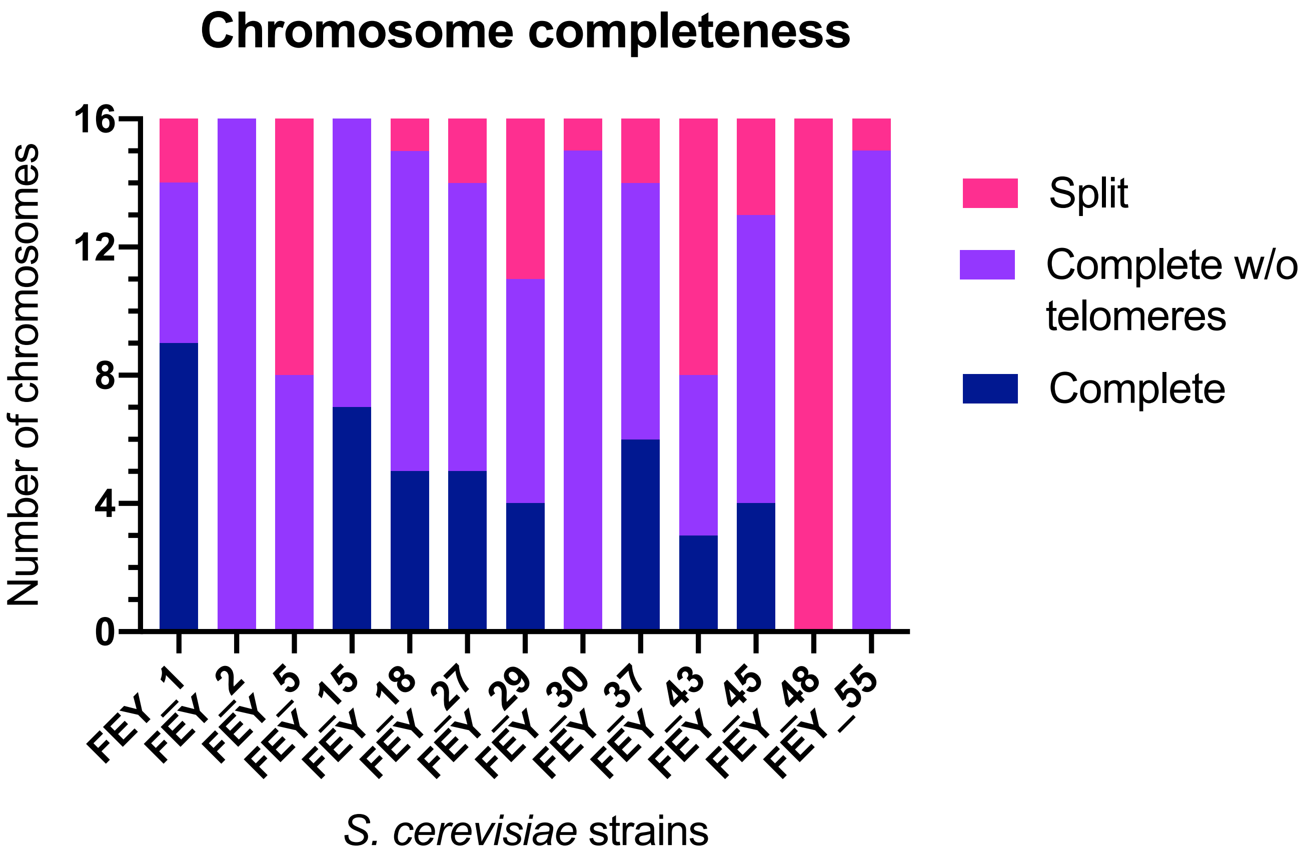

Supplementary Figure 9. Chromosome completeness assessment of the engineered *S. cerevisiae* strains. Complete chromosomes were those that contained a telomere-to-telomere alignment to their respective reference chromosomes. Complete w/o telomeres chromosomes were those that aligned to their respective reference chromosomes, save the telomeres. Split chromosomes were those where the reference chromosome was split into two or more contigs in the engineered assembly.

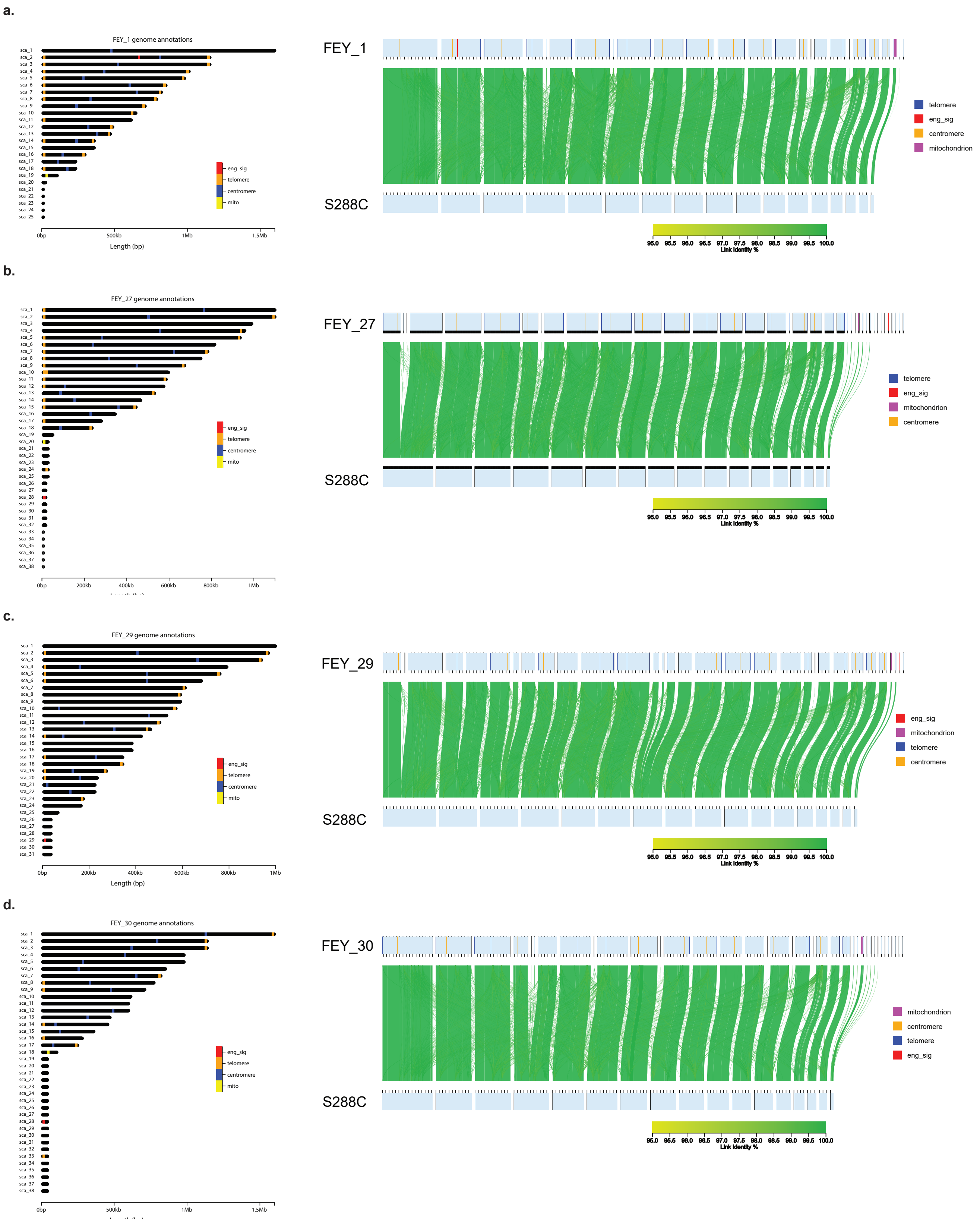

Supplementary Figure 10. Chromomap and AliTV alignments for S288C-derived engineered yeast strains, part 1.

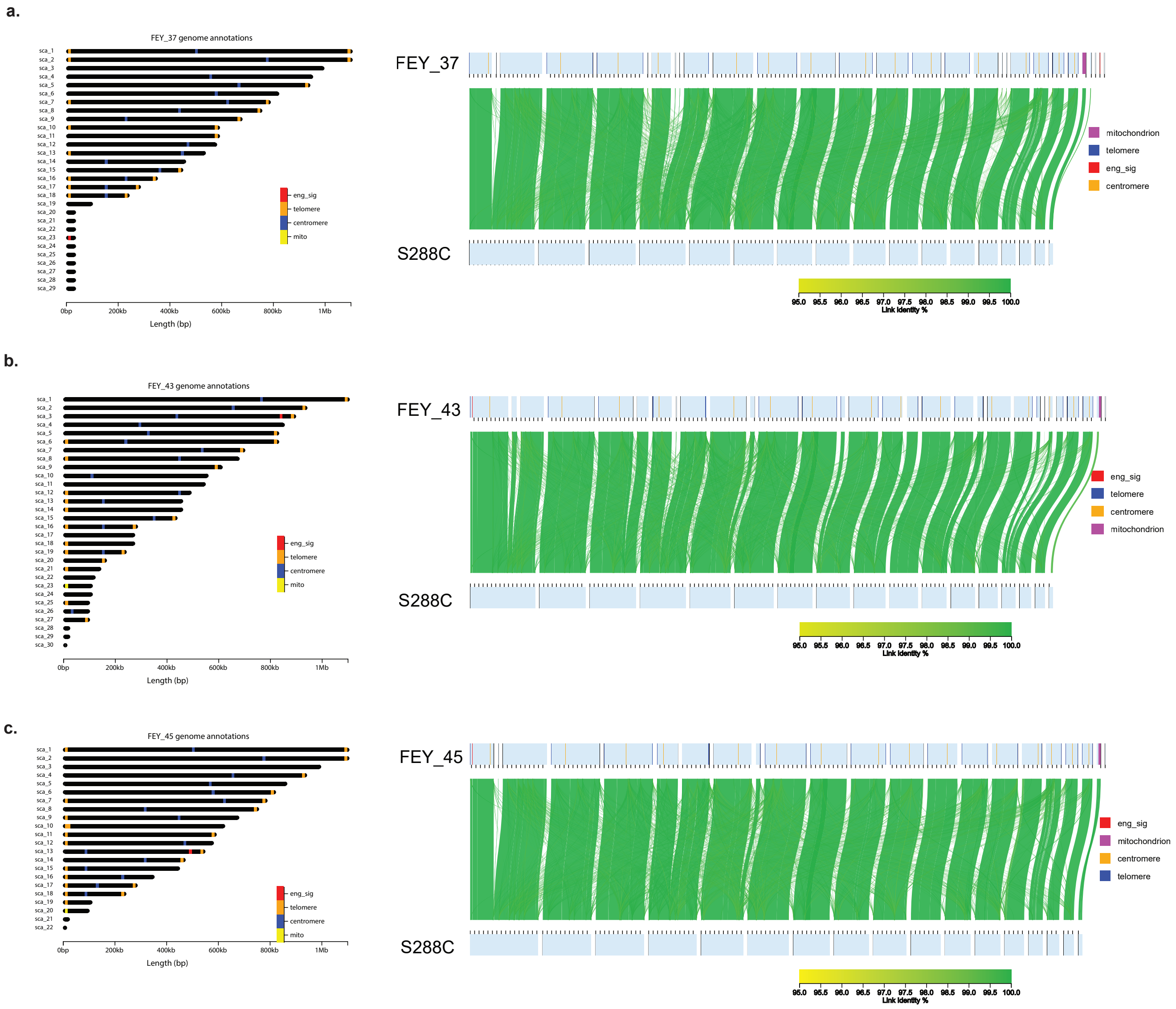

Supplementary Figure 11. Chromomap and AliTV alignments for S288C-derived engineered yeast strains, part 2.

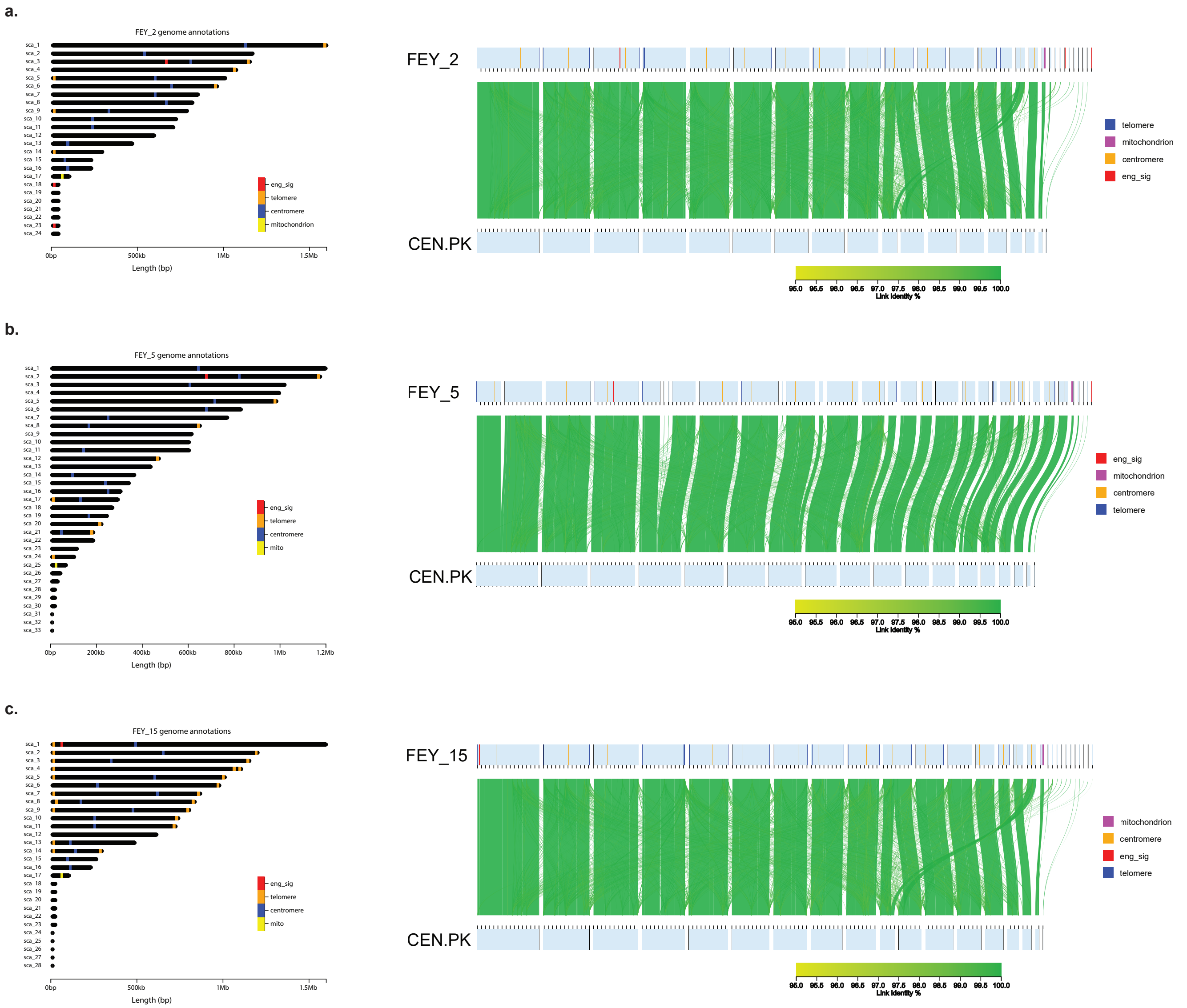

Supplementary Figure 12. Chromomap and AliTV alignments for CEN.PK-derived engineered yeast strains.

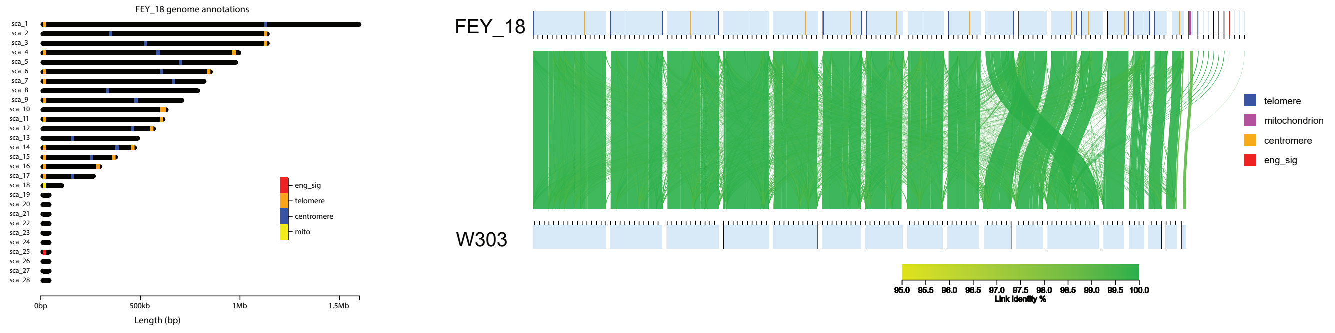

Supplementary Figure 13. Chromomap and AliTV alignments for W303α-derived engineered yeast strains.

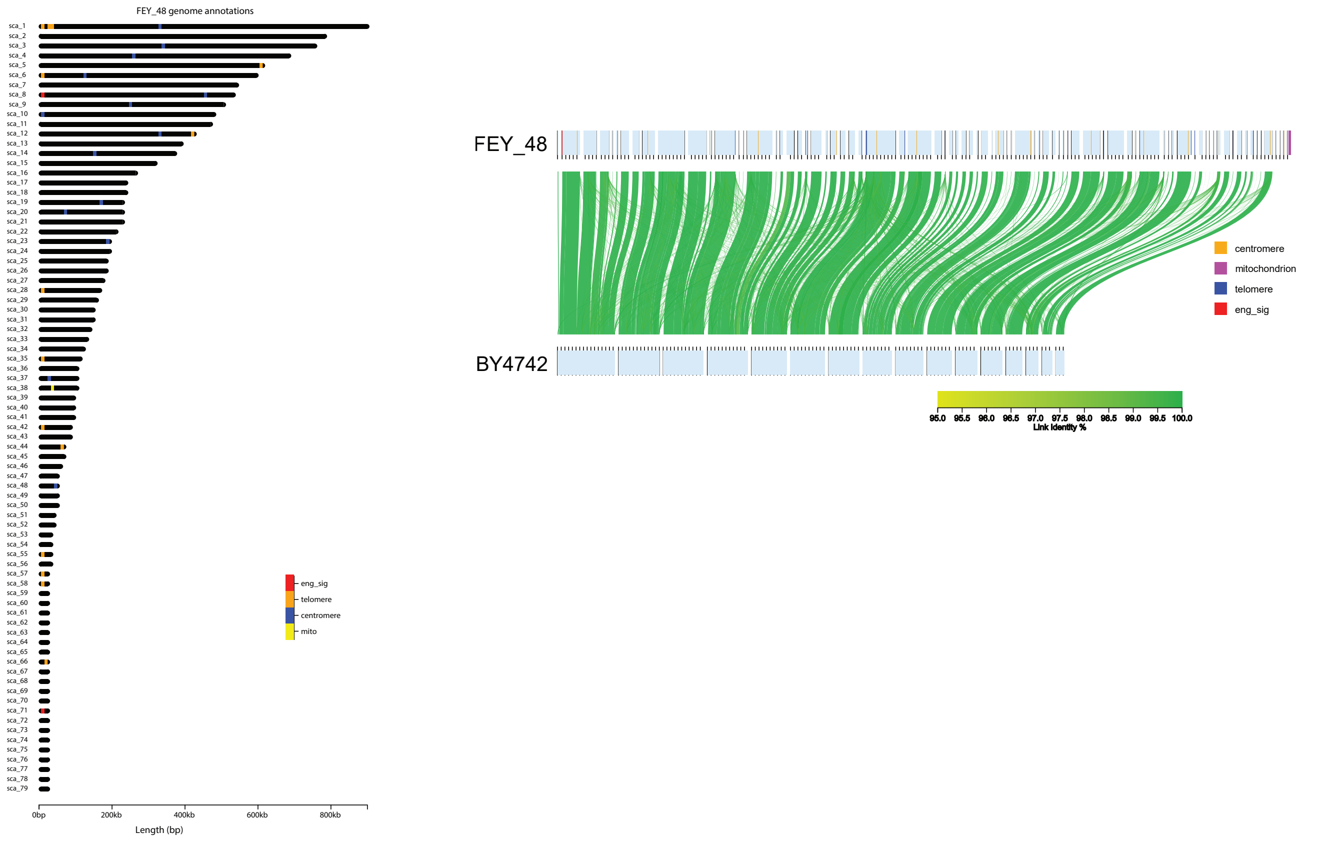

Supplementary Figure 14. Chromomap and AliTV alignments for BY4742-derived engineered yeast strains.

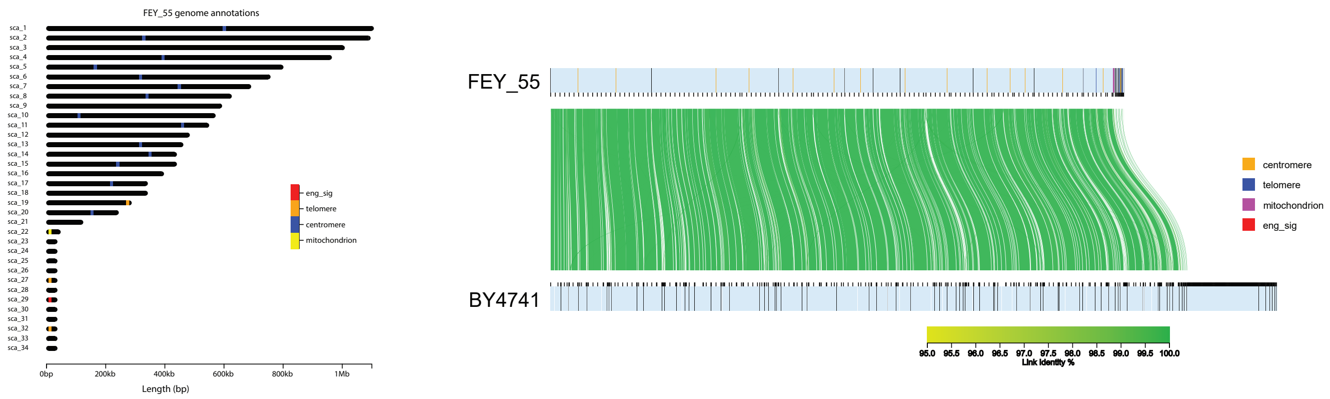

Supplementary Figure 15. Chromomap and AliTV alignments for BY4741-derived engineered yeast strains.

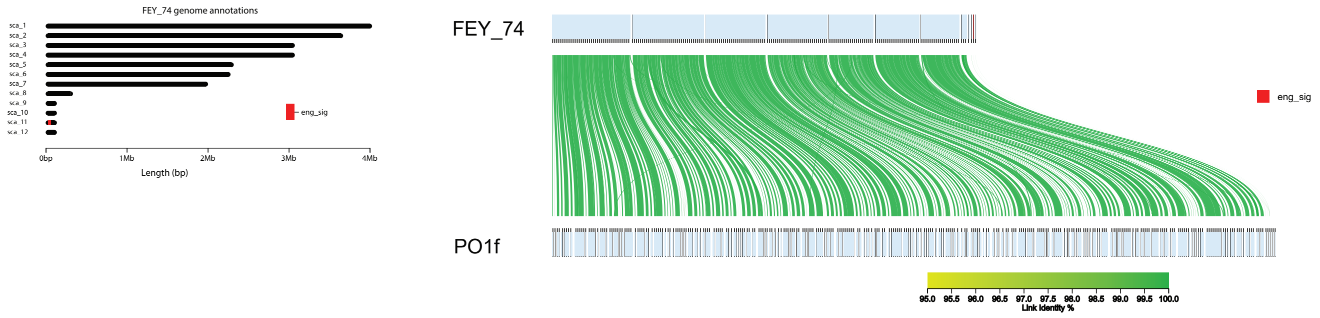

Supplementary Figure 16. Chromomap and AliTV alignments for *Y. lipolytica*-derived engineered yeast strains.

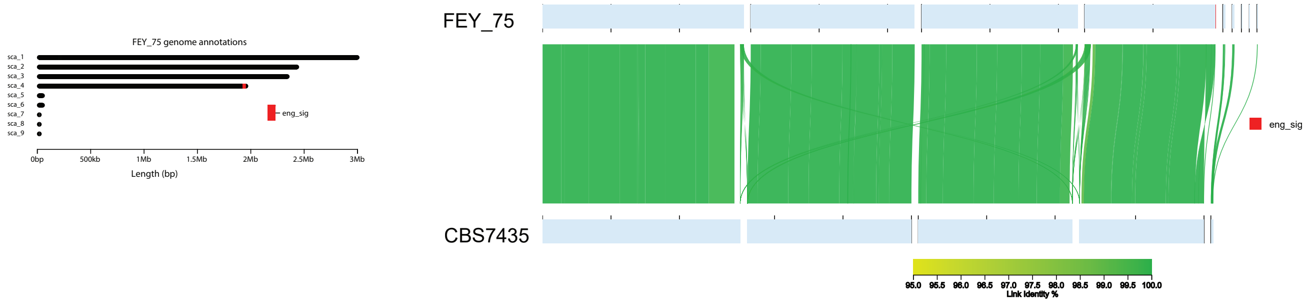

Supplementary Figure 17. Chromomap and AliTV alignments for *K. phaffii*-derived engineered yeast strains.

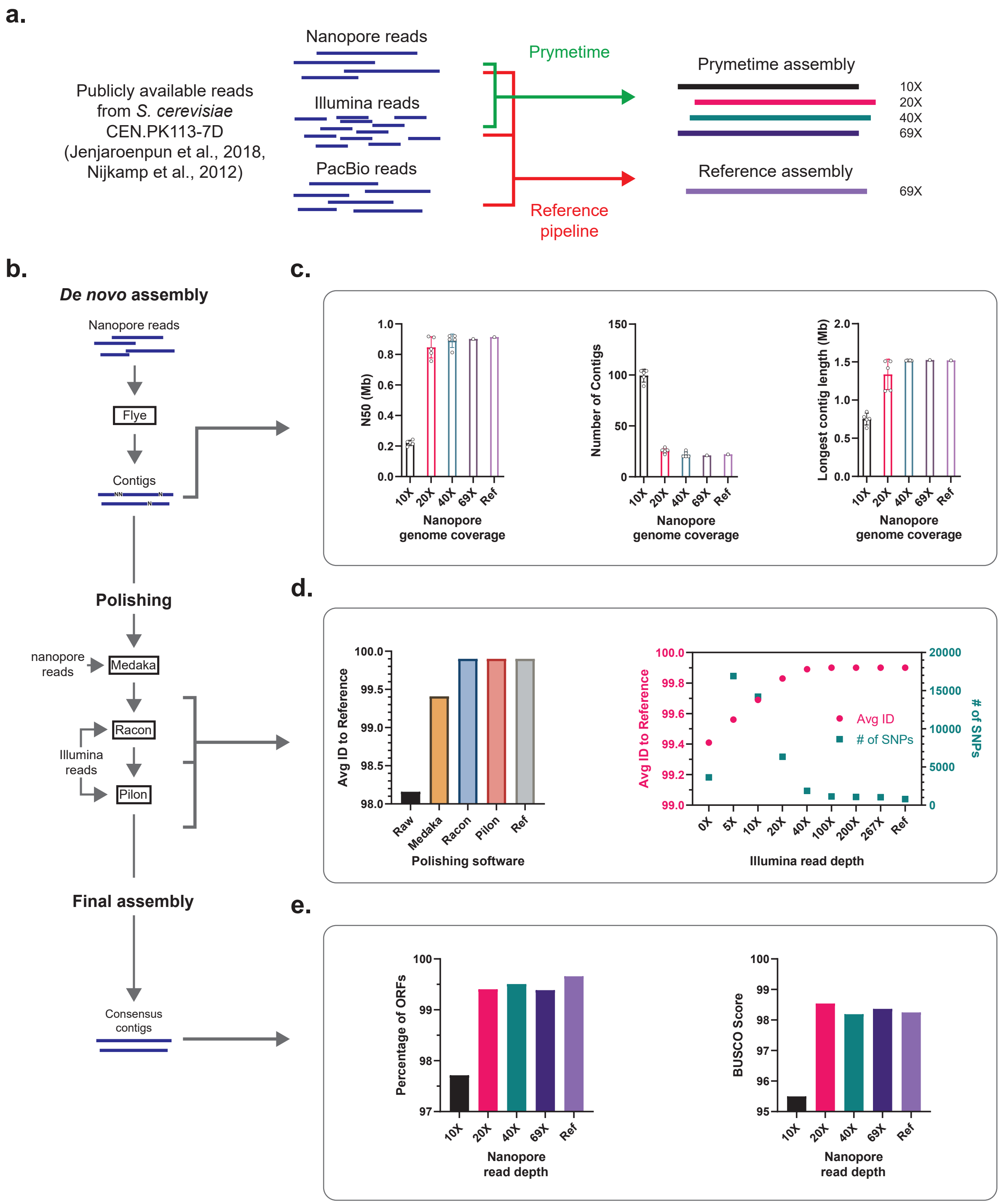

Supplementary Figure 18. Step-by-step validation of the Prymetime workflow with publicly available CEN.PK-113 reads. **a.** Use of the reads for assembly comparison. **b.** Flowchart for the workflow, indicating each step assessed. **c.** Contig comparison between Prymetime and reference. Random read libraries (n=5) were generated at each of the 10X, 20X, and 40X nanopore genome coverages. The 69x genome coverage (n=1) was simply all of the raw reads generated, while the reference (Ref, n=1) was a CEN.PK113 genome assembly from a previous study. The error bars represent the mean ± standard deviation. **d.** Improvement of sequence accuracy by successive polishing steps, as compared to reference. **e.** Whole genome quality of Prymetime whole genomes as compared to reference, evaluated by the percentage of ORFs and BUSCO score.

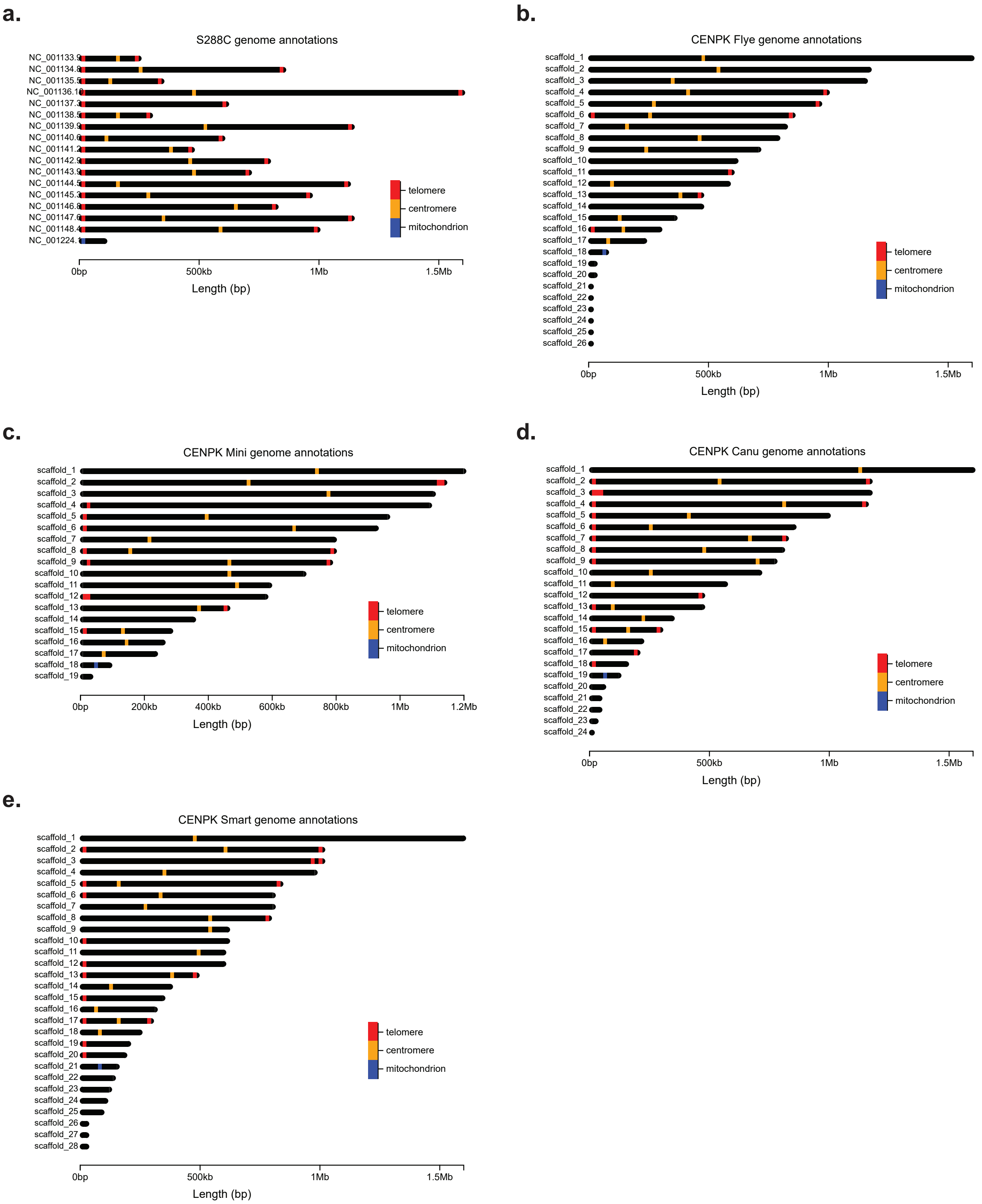

Supplementary Figure 19. chromoMap visualization of genome structure elements for four nanopore *de novo* assemblers compared to the S288C reference assembly.

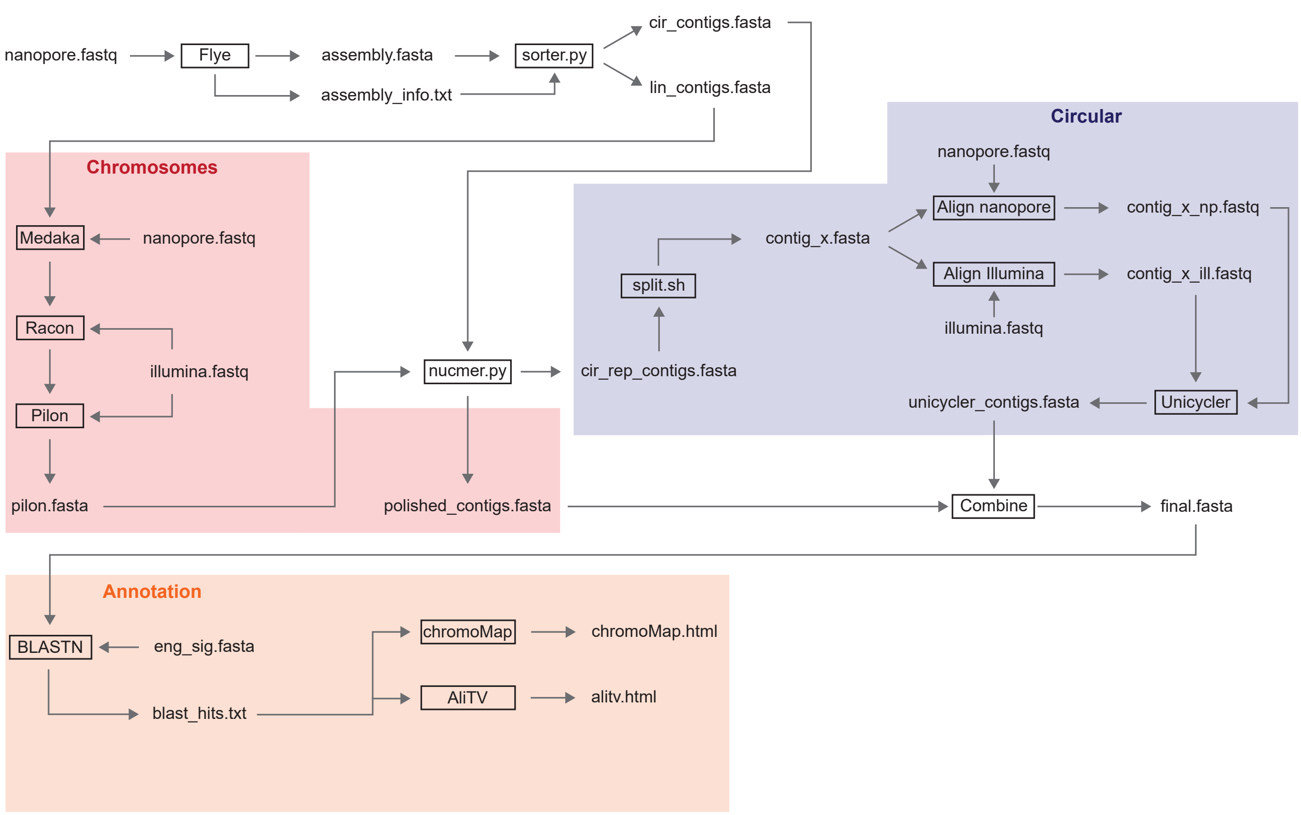

Supplementary Figure 20. Detailed illustration of the Prymetime workflow.

Supplementary References

1. Young, E. M. *et al.* Iterative algorithm-guided design of massive strain libraries, applied to itaconic acid production in yeast. *Metab. Eng.* **48**, 33–43, DOI: https://doi.org/10.1016/j.ymben.2018.05.002 (2018).
2. Lee, M. E., DeLoache, W. C., Cervantes, B. & Dueber, J. E. A highly characterized yeast toolkit for modular, multipart assembly. *ACS Synth. Biol.* **4**, 975–986 (2015).
3. Verwaal, R., Buiting-Wiessenhaan, N., Dalhuijsen, S. & Roubos, J. A. CRISPR/Cpf1 enables fast and simple genome editing of *Saccharomyces cerevisiae*. *Yeast* **35**, 201–211, DOI: 10.1002/yea.3278 (2018). 28886218[pmid].
4. Hegemann, J. H. & Heick, S. B. *Delete and Repeat: A Comprehensive Toolkit for Sequential Gene Knockout in the Budding Yeast Saccharomyces cerevisiae*, 189–206 (Humana Press, Totowa, NJ, 2011).
5. Schwartz, C. M., Hussain, M. S., Blenner, M. & Wheeldon, I. Synthetic RNA polymerase III promoters facilitate high-efficiency CRISPR-Cas9-mediated genome editing in *Yarrowia lipolytica*. *ACS Synth. Biol.* **5**, 356–359 (2016).
6. Obst, U., Lu, T. K. & Sieber, V. A modular toolkit for generating *Pichia pastoris* secretion libraries. *ACS Synth. Biol.* **6**, 1016–1025, DOI: 10.1021/acssynbio.6b00337 (2017).
7. van den Berg, M. A. *et al.* Genome sequencing and analysis of the filamentous fungus *Penicillium chrysogenum*. *Nat. Biotechnol.* **26**, 1161–1168, DOI: 10.1038/nbt.1498 (2008).
8. Farzadfard, F., Perli, S. D. & Lu, T. K. Tunable and multifunctional eukaryotic transcription factors based on CRISPR/Cas. *ACS Synth. Biol.* **2**, 604–613, DOI: 10.1021/sb400081r (2013).
9. DiCarlo, J. E. *et al.* Genome engineering in *Saccharomyces cerevisiae* using CRISPR-Cas systems. *Nucleic Acids Res.* **41**, 4336–4343, DOI: 10.1093/nar/gkt135 (2013). https://academic.oup.com/nar/article-pdf/41/7/4336/25342046/gkt135.pdf.
10. Berman, C. M. *et al.* An adaptable platform for directed evolution in human cells. *J. Am. Chem. Soc.* **140**, 18093–18103, DOI: 10.1021/jacs.8b10937 (2018).
11. Voth, W. P., Richards, J. D., Shaw, J. M. & Stillman, D. J. Yeast vectors for integration at the HO locus. *Nucleic Acids Res.* **29**, e59–e59, DOI: 10.1093/nar/29.12.e59 (2001). http://oup.prod.sis.lan/nar/article-pdf/29/12/e59/9905922/2900e59.pdf.
