# Supplementary material for: Engineered yeast genomes accurately assembled from pure and mixed samples
Source: Nat Commun. 2021 Mar 5;12:1485. doi: 10.1038/s41467-021-21656-9 (PMC7935868; doi:10.1038/s41467-021-21656-9)
Supplement: Supplementary file 2 — Reporting Summary [file 41467_2021_21656_MOESM2_ESM.pdf]

nature research

Corresponding author(s): Eric M. Young

Last updated by author(s): Jan 26, 2021

Reporting Summary

Nature Research wishes to improve the reproducibility of the work that we publish. This form provides structure for consistency and transparency in reporting. For further information on Nature Research policies, see our Editorial Policies and the Editorial Policy Checklist.

Statistics

For all statistical analyses, confirm that the following items are present in the figure legend, table legend, main text, or Methods section.

n/a	Confirmed	
[ ]	[x]	The exact sample size (n) for each experimental group/condition, given as a discrete number and unit of measurement
[ ]	[x]	A statement on whether measurements were taken from distinct samples or whether the same sample was measured repeatedly
[x]	[ ]	The statistical test(s) used AND whether they are one- or two-sided<br>*Only common tests should be described solely by name; describe more complex techniques in the Methods section.*
[ ]	[x]	A description of all covariates tested
[ ]	[x]	A description of any assumptions or corrections, such as tests of normality and adjustment for multiple comparisons
[ ]	[x]	A full description of the statistical parameters including central tendency (e.g. means) or other basic estimates (e.g. regression coefficient) AND variation (e.g. standard deviation) or associated estimates of uncertainty (e.g. confidence intervals)
[x]	[ ]	For null hypothesis testing, the test statistic (e.g. F, t, r) with confidence intervals, effect sizes, degrees of freedom and P value noted<br>*Give P values as exact values whenever suitable.*
[x]	[ ]	For Bayesian analysis, information on the choice of priors and Markov chain Monte Carlo settings
[ ]	[x]	For hierarchical and complex designs, identification of the appropriate level for tests and full reporting of outcomes
[x]	[ ]	Estimates of effect sizes (e.g. Cohen's d, Pearson's r), indicating how they were calculated

Our web collection on statistics for biologists contains articles on many of the points above.

Software and code

Policy information about availability of computer code

Data collection

The MinKNOW interface (v8.3.1) was used to generate nanopore fast5 reads. Nanopore fast5 files were basecalled using Guppy v2.3.5 (Oxford Nanopore base caller). The subsequent fastq files were demultiplexed using the EPI2ME interface v2019.12.13 (Metrichor, Oxford, UK). Illumina reads were demultiplexed using the Local Run Manager v1.0.0 on the iSeq machine. Random subsets of Illumina and Nanopore reads at a specific genome coverage were generated using a custom python script (https://github.com/aseetharam/common_scripts/blob/master/sample_fastq.py).

Data analysis

For the MiniASM assembly, reads were first mapped using minimap2 (v2.17-r941) with the parameters "-x ava-ont -t8". MiniASM (v0.3) was then subsequently run with the default parameters. Canu (v1.8) was run with the parameters "minReadLength=2500 mhapSensitivity=high corMhapSenstivity=high corOutCoverage=500". SMARTdenovo (v1.0) was run using the parameters "-c 1 -k 14 -J 2500 -e zmo". Flye (v2.4) was run with the parameters "–meta –plasmids". ABySS (v2.1.5) was run with the abyss-pe option and the parameter "k=96". Edena (v3.131028) was run with the default parameters. Velvet (v1.2.10) was run with a hashlength of 21 bp. MaSuRCA (v3.3.4) was run with the parameter "JF_SIZE = 242000000 FLYE_ASSEMBLY=1". SPAdes (v3.13.1) was run with the parameters "–sc –nanopore –pe< # >-1 –pe< # >-2".

The de novo Nanopore genome assemblies were first polished with Nanopore reads using Medaka (v0.4) with the default parameters. The assembly was then polished with Illumina reads, first with Racon (v1.3.1) followed by Pilon (v1.22). For Racon, the Illumina reads were first mapped to an assembly using minimap2 with the parameter "-ax sr". Racon was then run using the default parameters. For Pilon, assemblies were first indexed using bwa (v0.7.17-r1188). Illumina reads were then mapped to the assembly using bwa with the parameter "mem –t 14". Pilon was then run using the parameter "-Xmx160G".

To find potential circular contigs that Flye may have missed, a custom python script was used on the polished linear contigs file. The script used the Mummer (option nucmer (v3.1) with the parameters "maxmatch=True, simplify=False, mincluster=2000, min_id=99, min_length=2000, coords_header=True" on contigs that were less than 50,000 bp to identify repetitive contigs. The repetitive contigs were extracted and combined with the circular contigs from the initial Flye assembly, and sent to be re-assembled with Unicycler (v0.4.8). In order

to do this, the contigs were first separated into separate fasta files using awk (v4.0.2). Nanopore and Illumina reads were then mapped to each individual contig, with matches extracted into fastq files. The Nanopore reads were mapped using minimap2 with the parameters "-ax map-ont" followed by extraction of hits with samtools (v1.9) and the parameters "fastq -n -F 4 -". Each paired-end Illumina file was mapped using minimap2 with the parameter "-ax sr" followed by extraction of hits with samtools and the parameters "fastq -n -F 4 -". The resulting two Illumina files were paired using fastq_pair (v1.0) with the default parameters. Unicycler was then run with the mapped and paired Illumina files along with the mapped Nanopore file with the default parameters.

Non-native engineering signatures were detected in the genome assemblies using BLASTN (v2.5.0) with the parameters "-perc_identity 98 -qcov_hsp_perc 98". The query for this BLASTN search was a curated list of all non-native engineering signatures used to engineer the yeast strains in this study, and are included in the Prymetime package. The genome features telomeres, centromeres, and mitochrondrion were detected using BLASTN with the parameters "-max_target_seqs 1 -max_hsps 1". The genome feature sequences were downloaded from the Saccharomyces Genome Database, and are included in the Prymetime package. The genome plotter chromoMap (v0.2) was run with the parameters "data_based_color_map = T, data_type = "categorical" to show the engineering signatures and genome elements hits from the BLASTN search in the context of the entire genome assembly. The genome alignment and visualization software AliTV (v1.0.6) was run with the default parameters.

QUAST (v5.0.0) was run with the default parameters, yielding the metrics number of contigs, maximum contig length, and N50. For accuracy related metrics, the nucmer command was run as part of the MUMmer package. The command "dnadiff -d" was used on the resulting delta file to find the average identity to the reference and the number of SNPs. Genome assemblies were evaluated for genome completeness using BUSCO (v4.0.6) with the saccharomycetales_odb9 datasets, as well as a BLASTN search of ORFs from S. cerevisiae S288C. Engineered signatures were searched for in genome assemblies using BLASTN with the expect threshold set at 0.0001.

For manuscripts utilizing custom algorithms or software that are central to the research but not yet described in published literature, software must be made available to editors and reviewers. We strongly encourage code deposition in a community repository (e.g. GitHub). See the Nature Research guidelines for submitting code & software for further information.

Data

Policy information about availability of data

All manuscripts must include a data availability statement. This statement should provide the following information, where applicable:

- Accession codes, unique identifiers, or web links for publicly available datasets
- A list of figures that have associated raw data
- A description of any restrictions on data availability

Illumina and nanopore raw reads from all engineered yeast strains have been deposited to DDBJ/ENA/GenBank under the BioProject PRJNA650312. Illumina and nanopore raw reads from the non-engineered yeast strains have been deposited to DDBJ/ENA/GenBank under the BioProject PRJNA694170. All yeast genome assemblies from this study (engineered and non-engineered) are available in https://github.com/emyounglab/prymetime_genomes.

Field-specific reporting

Please select the one below that is the best fit for your research. If you are not sure, read the appropriate sections before making your selection.

☒ Life sciences ☐ Behavioural & social sciences ☐ Ecological, evolutionary & environmental sciences

For a reference copy of the document with all sections, see nature.com/documents/nr-reporting-summary-flat.pdf

Life sciences study design

All studies must disclose on these points even when the disclosure is negative.

Sample size	The sample size of 15 engineered yeast was determined so that multiple strains of S. cerevisiae and nonconventional yeast could be included in the set of genome assemblies, also accounting for plasmids and integrated pathways. Thus we arrived at 15 through the process of being inclusive of common genetic backgrounds and engineering types. The sample size for each sequenced engineered yeast strain was 1, only one clone of engineered yeast was sequenced. However, we achieved at least 40X coverage of the genome, and sequenced with two independent technologies, to give confidence in the accuracy of the basecalls. These coverage depths are standard in the field and also were appropriate based on our internal analysis as presented in the manuscript.<br><br>When analyzing software performance with different genome coverages, at least five random slices of the data were used. This is a common method in bioinformatics and machine learning where different random training datasets are used to eliminate bias in the data slice. Doing the same for our data showed that the assembly steps were robust to input data, and the metrics used to evaluate the step didn't vary appreciably with different input data. These sample sizes were used to calculate standard deviations in the assembly metrics.
Data exclusions	There was no data excluded from analysis. All reads were fed into the analysis software.
Replication	When analyzing software performance with different genome coverages, at least three and often five random slices of the data were used. This would eliminate any assembly failures specifically due to the data in the slice. With this approach, the results we found were replicable.
Randomization	Randomization was used to slice data, as mentioned in the above section. Also mentioned previously, we included a large number of genetic backgrounds and three different yeast species to diversify the data being fed to our software, in an effort to make the tool generalizable. This is a kind of randomization where varied genetic backgrounds are used to challenge the software.

Blinding	Blinding was not used because comparative analysis was essential to the performance of our software. That is, determination of the sequence accuracy required that we match a yeast assembly with its parent assembly and the expected assembly sequence. Only by doing this were we able to determine that the tool was performing correctly.<br><br>However, since we used the same approach to sequence each yeast, this is a kind of blinding where we did not bias any wet lab or computational procedures based on the origin of the data. This is also true of the experiment where we compared our CENPK assembly to a publicly available assembly. Our software was "blind" to the origin of the reads and the strain to be assembled.<br><br>Finally, performing de novo assembly is a type of blinding for the software as it does not have a scaffold genome on which to map the reads. Performing exclusively de novo assembly was necessary to correctly assemble engineering, but also does not bias the assembly process.

Reporting for specific materials, systems and methods

We require information from authors about some types of materials, experimental systems and methods used in many studies. Here, indicate whether each material, system or method listed is relevant to your study. If you are not sure if a list item applies to your research, read the appropriate section before selecting a response.

Materials & experimental systems

n/a	Involved in the study	
☒	☐	Antibodies
☐	☒	Eukaryotic cell lines
☒	☐	Palaeontology and archaeology
☒	☐	Animals and other organisms
☒	☐	Human research participants
☒	☐	Clinical data
☒	☐	Dual use research of concern

Methods

n/a	Involved in the study	
☒	☐	ChIP-seq
☒	☐	Flow cytometry
☒	☐	MRI-based neuroimaging

Eukaryotic cell lines

Policy information about cell lines

Cell line source(s)	The eukaryotes used in this study were yeast. S. cerevisiae S288C, S. cerevisiae W303-alpha, S. cerevisiae BY4741, S. cerevisiae BY4742, S. cerevisiae BY4743, S. cerevisiae CEN.PK-113, Y. lipolytica Po1f, and K. phaffii ATCC 76273
Authentication	Whole genome sequencing
Mycoplasma contamination	Not applicable to yeasts.
Commonly misidentified lines<br>(See ICLAC register)	Not applicable to yeasts.
